# Mild movement sequence repetition in five primate species and evidence for a taxonomic divide in cognitive mechanisms

**DOI:** 10.1038/s41598-022-18633-7

**Published:** 2022-08-25

**Authors:** L. Tamara Kumpan, Alexander Q. Vining, Megan M. Joyce, William D. Aguado, Eve A. Smeltzer, Sarah E. Turner, Julie A. Teichroeb

**Affiliations:** 1 Anthropology, University of Toronto Scarborough, 1265 Military Trail, Toronto, ON M1C 1A4 Canada; 2 School of the Environment, University of Toronto, Toronto, Canada; 3 Animal Behavior Graduate Group, University of California, Davis, Davis, USA; 4 Department for the Ecology of Animal Societies, Max Planck Institute of Animal Behavior, Konstanz, Germany; 5 Geography, Planning and Environment, Concordia University, Montréal, Canada; 6 Anthropology, Rutgers University, New Brunswick, USA

**Keywords:** Biological anthropology, Animal behaviour, Behavioural ecology

## Abstract

When animals forage, they face complex multi-destination routing problems. Traplining behaviour—the repeated use of the same route—can be used to study how spatial memory might evolve to cope with complex routing problems in ecologically distinct taxa. We analyzed experimental data from multi-destination foraging arrays for five species, two cercopithecine monkeys (vervets, *Chlorocebus pygerythrus*, and Japanese macaques, *Macaca fuscata*) and three strepsirrhines (fat-tailed dwarf lemurs, *Cheirogaleus medius*, grey mouse lemurs, *Microcebus murinus*, and aye-ayes, *Daubentonia madagascariensis*). These species all developed relatively efficient route formations within the arrays but appeared to rely on variable cognitive mechanisms. We found a strong reliance on heuristics in cercopithecoid species, with initial routes that began near optimal and did not improve with experience. In strepsirrhines, we found greater support for reinforcement learning of location-based decisions, such that routes improved with experience. Further, we found evidence of repeated sequences of site visitation in all species, supporting previous suggestions that primates form traplines. However, the recursive use of routes was weak, differing from the strategies seen in well-known traplining animals. Differences between strepsirrhine and cercopithecine strategies may be the result of either ecological or phylogenetic trends, and we discuss future possibilities for disentangling the two.

## Introduction

Foraging animals face complex multi-destination routing problems as they move between food sites. By locating routes that strategically connect biologically meaningful locations across space, animals may benefit from increased route efficiency during travel. However, finding the most efficient path connecting multiple sites requires the cognitive capacity to cope with the classic mathematical problem of the travelling salesperson (TSP), in which a set of fixed locations are each visited once before returning to the point of origin^1,2^. When returning to the start is not required, the problem is termed an optimal Hamiltonian path problem, open-TSP, or shortest path problem^3^. The number of possible routes in a TSP-like problem increases exponentially as sites are added, quickly making computation of the most efficient route intractable. Animals with differing computational abilities likely evolved alternative strategies to address these navigational challenges in the wild^4^.

One approach to solving TSP-like foraging problems is to learn, through trial and error, efficient sequences of location transitions within an array; a method sometimes called iterative learning. In bumblebees, an individual-based decision model of this type has been used to explain traplining behaviour^5,6^—a foraging strategy that involves the repeated use of a circuit to feeding sites in a stable and predictable sequence^7^. In simulations and foraging experiments, bumblebees eventually converge on an optimal or near-optimal route through simple arrays via trial and error with iterative improvement, where the likelihood of a given transition between locations increases if that transition was used in a trial that resulted in a final path length decrease, relative to previous trials^6^.

Alternatively, animals may utilize a different cognitive process, using pre-existing heuristics^8^, which are simple “rules of thumb,” either learned or innate, for solving multi-destination routes. For example, animals may prefer to always move to the nearest location that has not yet been visited—a strategy called the nearest-neighbour rule (NNR)^9^. This appears to be a solution used by a variety of taxa to solve navigational challenges due to the low cognitive effort it requires (bees^9^; rats^10^; non-human primates^11–13^). Animals might also rely on other heuristics such as the “convex hull,” where a mental loop is placed around the targets and visits occur in order based on distance from the outer edge^13–15^. Importantly, though many heuristic rules are possible^4^, it can be expected that those that are most generalizable to the greatest number of resource distributions should be the most adaptive. The full suite of heuristics that humans use to solve TSP-like problems is still unknown and new possibilities continue to be generated^16^. One approach to understanding human heuristics and how such heuristics emerge is to study how our closest relatives, primates, solve similar challenges within ecological contexts.

Several cognitive processes have been proposed as drivers of trapline foraging, including iterative learning and nearest-neighbour heuristics^17^. Thus, traplining behaviour can inform how spatial memory might evolve to cope with complex routing problems in ecologically distinct taxa^18^. Further, primate use of traplining has been suggested in the literature^19–23^; however, these studies did not explicitly test the hypothesis that primates form traplines.

Investigating whether primates use traplines, how quickly they develop traplines, and how closely their routes resemble standard traplines, would reveal how they manage potentially complex trade-offs between reinforcement learning, cognitively simple heuristics, and the development of more complex, but flexible heuristics.

To examine primate strategies for approximating solutions to multi-destination routes, we analyzed movement data from multi-destination foraging arrays for five species, two cercopithecine monkeys and three strepsirrhines: vervet monkeys (*Chlorocebus pygerythrus*), Japanese macaques (*Macaca fuscata*), fat-tailed dwarf lemurs (*Cheirogaleus medius*), grey mouse lemurs (*Microcebus murinus*), and aye-ayes (*Daubentonia madagascariensis*). We measured the recursive movement characteristic of traplining behavior by quantifying 1) the repetitiveness of foraging sequences in which individuals completed experimental arrays over multiple trials, and 2) the distance travelled in the arrays fitted to simple learning curves. Foraging arrays were originally designed for previous studies that examined different theoretical questions^12,13,15,24–26^ and thus they vary for some species, however these data are useful to compare these five primate species in their navigational strategies. Each array required decisions in small-scale space where all sites were visible to one another and exploration between platforms was not required.

We predicted differences in movement patterns based on known dietary and ecological differences between our study species (Table 1)^27^. Fat-tailed dwarf lemurs are small nocturnal cheirogaleid strepsirrhines that are frugivorous^28^. Since they exhibit a strong reliance on stationary replenishing food items and have been found to improve their accuracy and speed with experience in a multi-destination array^13^, we predicted that they would show a decrease in distance traveled with experience consistent with an iterative improvement model. Grey mouse lemurs are also small nocturnal cheirogaleid strepsirrhines but exhibit pronounced feeding plasticity, being omnivorous and consuming fruit, gum, insect secretions, and small vertebrates^29^. Aye-ayes are larger nocturnal strepsirrhines and are also known to exhibit diverse diets depending on where they live, relying on insects in primary forest^30,31^ and consuming fruits, coconut, flowers, and flower nectar in secondary forests^32,33^. For these two species that exhibit a stronger reliance on ephemeral and mobile food items, we predicted a minor decrease in distance traveled with experience, and minimally repetitive route sequences. This prediction is supported by results showing more randomness (i.e., rarely repeating foraging paths in experimental set-ups) in the navigation patterns of strepsirrhines relying on ephemeral resources^13^. In addition, while vervet monkeys are medium-bodied African monkeys that rely mainly on fruits and flowers and opportunistically prey on insects^34,35^, Japanese macaques are medium-bodied Asian monkeys with a diet that varies as a function of forest type but generally includes new and mature leaves, flowers, fruits, insects, and fungi^36,37^. Since vervets have previously been shown to rely strongly on navigational heuristics^11,12,15,24^ and Japanese macaques have similar diets, we predicted that these species would show patterns consistent with heuristic use—distance traveled and route-repetition would both stay consistent throughout trials, but distance traveled would be significantly lower and route-repetition significantly higher when compared to our simulations of other navigational strategies.

**Table 1:** Socio-ecological traits of the five primate species included in our sample.

Species	Activity pattern	Social structure	Levels of food consumed				
			Leaves	Insects	Fruit	Gum	Flowers/nectar
**Strepsirrhines**							
Fat-tailed dwarf lemurs (*C. medius*)^38,39^	Nocturnal	Small groups	None	Low	High	None	High
Grey mouse lemur (*M. murinus*)^40,41^	Nocturnal	Solitary foragers	Low	High	Mod	High	Mod
Aye-ayes (*D. madagascariensis*)^42^	Nocturnal	Solitary	None	High	High	Low	Mod
**Cercopithecines**							
Vervet monkeys (*C. pygerythrus*)^43^	Diurnal	Large groups	Mod	Low	High	None	High
Japanese macaques (*M. fuscata*)^44^	Diurnal	Large groups	High	Low	High	None	Mod

We tested two hypotheses focused on two different cognitive processes to understand how primates approximate solutions to multi-destination routes. H1—Primates use reinforcement learning of location-based decisions that are more likely to lead to shorter and more consistent routes and less travel with experience. This is a slow and cognitively costly process of information acquisition and alteration of behaviour that may require spatial rehearsal within visuo-spatial working memory and mental ‘chunking’ of locations together^45^. H2—Primates could use a less cognitively costly process, heuristics, that can be generalized to approximate solutions to multi-destination routes in many different arrays and if the best heuristics are applied, lead to both distance traveled and route repetition remaining relatively consistent throughout trials. Additionally, under this hypothesis, we would expect animals to immediately use more efficient paths through resource arrays than expected by random decision making. Under the null hypotheses of random decision-making, we would expect to see no decrease in distance traveled with experience and minimally repetitive route sequences. Since traplining is beneficial for renewable and spatially predictable resources, ecological variables may also predict traplining in primates. Specifically, frugivorous species or species that rely on renewable, predictable resources should be more likely to trapline than species relying on insects or other mobile, non-renewable resources^46^.

We also anticipated that our results might be impacted by living conditions because some species were tested in captivity whereas others were wild. This could lead to many variables altering experimental outcomes, such as internal factors influencing motivation, unknown variables such as diet, health status, distractions faced by wild but not captive animals, and unnatural territory sizes experienced in captivity. Coincidentally, all strepsirrhines were captive and both cercopithecoids were wild, so these potentially confounding factors may lead to findings of similar navigational strategies for strepsirrhines versus catarrhines.

## Methods

### Study subjects

We conducted foraging experiments on strepsirrhines (*N*_*individuals*_ = 18) at the Duke Lemur Center (DLC), North Carolina, from February to November 2015^13^. Our sample includes six fat-tailed dwarf lemurs (3–16 years of age, 3 males, 3 females), six gray mouse lemurs (3–7 years of age, all female), and six aye-ayes (17–32 years of age, 2 males, 4 females). Because these species are solitary and nocturnal, most animals were housed singly and were kept on a reversed light cycle such that they were active and could be tested during the day. Housing conditions were similar for all individuals, and they were all fed daily in a similar manner with a diet that included fruits, vegetables, meal worms, and monkey chow (details in^13^).

All vervet data were collected on wild animals (*N*_*individuals*_ = 12) at Lake Nabugabo, Uganda (0°22′–12° S and 31°54′ E) during four separate field seasons (April-June 2013, Double Trapezoid array, M group^15^; June–September 2013, Pentagon array, M group^24^; August–September 2015, Z-array, M group^12^; July–August 2017, Pentagon array, KS group^25^). M group was composed of between 21–28 individuals, containing 2–3 adult males, 7–9 adult females, 2 subadult males, 1–3 subadult females, and 9–12 juveniles and infants. KS group was composed of 39–40 individuals including 5 adult males, 11 adult females, 3 sub-adult males, 5 sub-adult females, and 15–16 juveniles and infants. All individuals were reliably identified based on natural features (details in^12,15,24,25^). Outside of foraging experiments, wild vervets were not provision fed.

All Japanese macaque data (*N*_individuals_ = 10) were collected at the Awajishima Monkey Centre (AMC), Awaji Island, Japan (34°14′43.6″ N and 134°52′59.9″ E) between July and August 2019 (Z-array^26^). AMC is a privately-run tourist and conservation center visited by a large group of free-ranging Japanese macaques (~ 400 individuals) called the “Awajishima group”^47^. The group is composed of different-aged individuals of both sexes, with bachelor males and bachelor male groups living around the periphery^48^. The Awajishima group forages on wild foods for much of their dietary requirements but is also provision-fed a combination of wheat and soybeans, supplemented with peanuts, fruits, and vegetables twice daily for ~ 10 months of the year (details in^47,49,50^).

### Study design

#### Navigation arrays

**Figure 1:**
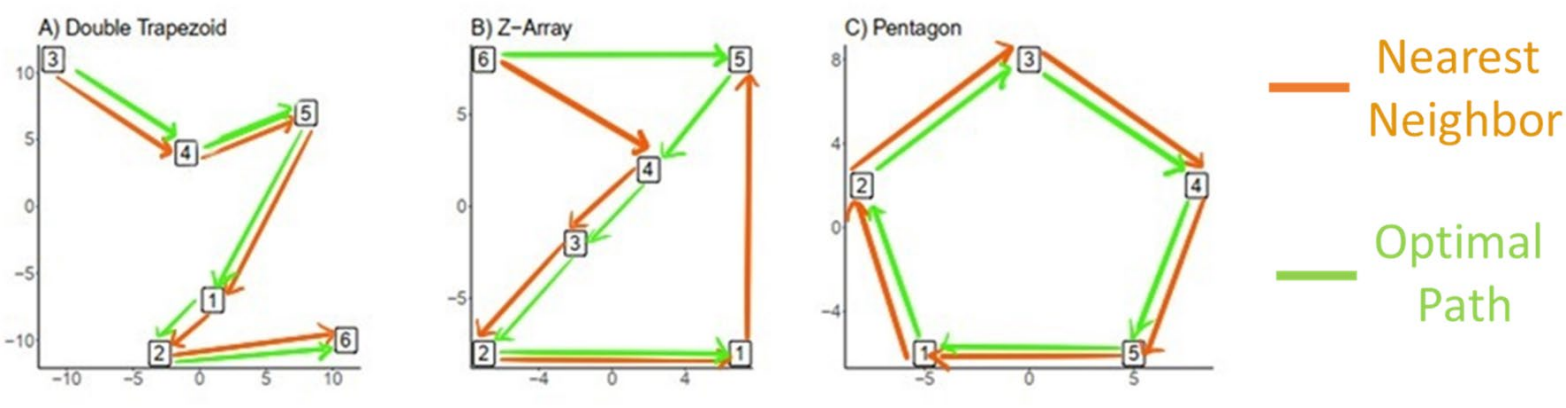
Design of the navigational arrays used, with (**a**) the Double Trapezoid array used for *Cheirogaleus medius*, *Microcebus murinus*, *Daubentonia madagascariensis*, and *Chlorocebus pygerythrus*. Three different arrays were built and scaled to the body size of animals (see “Methods”). (**b**) The Z-array used for *C. pygerythrus* and *Macaca fuscata*. The same size array was used for both species because they are similar in adult body lengths (vervet mean range from four sites: 34.5–42.6 cm^51^, Japanese macaque mean range from six sites: 48.9–59.7 cm^52^. (**c**) The Pentagon used for *C. pygerythrus.* Distances here are unitless but roughly proportional to the body size of each species tested. Created in R version 4.0.4 and ProCreate.

The strepsirrhines and vervets were tested on a “double-trapezoid” shaped multi-destination array with six feeding platforms^13,15^, modified from^17^ (Fig. 1a), where there were 720 possible routes (6!). Three different double-trapezoid arrays were built to account for differences in body size: one for the smaller dwarf and mouse lemurs, one for the mid-sized aye-ayes, and one for the larger, wild vervets. Arrays were scaled such that the distance from platform 1–2 (the shortest distance between targets) was approximately twice the body length of the subject species. Vervets were additionally tested on a Z-shaped array with six feeding platforms (720 possible routes, Fig. 1b^12^), and a pentagon-shaped array with five feeding platforms (120 possible routes, Fig. 1c^24,25,46^). Japanese macaques were tested on an identically sized Z-array^26^.

For strepsirrhine trials, DLC staff captured individuals in their enclosures and transported them in padded crates to the testing room. The dwarf and mouse lemur array was set up in a specially designed box (0.91 × 1.83 m) with a small compartment to contain strepsirrhines for rebaiting between trials. The aye-aye array was set up on the ground in a room measuring 2.44 × 4.27 m, where subjects stayed during the duration of their daily trials^13^. Vervet and macaque trials occurred when individual monkeys voluntarily left their group to participate in foraging experiments alone. Vervet arrays were set up using wooden feeding platforms (0.75 m long, 0.75 m wide × 0.75 m high) placed in an outdoor clearing measuring roughly 10 × 14 m in the home range of the study group. Japanese macaque arrays were also set up using small wooden feeding tables (0.40 m long, 0.30 m wide, 0.21 m high), covered in green plastic labeled with the platform number. Two identical arrays were built in neighbouring provision-feeding fields at the AMC (Near Lower Field: ~ 10 × 35 m, and Far Lower Field: ~ 15 × 45 m).

In these studies, all platforms were baited with a single food item. The reward used varied by species (strepsirrhines: grape piece, apple piece, honey, agave nectar, or nut butters, vervets: slice of banana, piece of popcorn; macaques: single peanut or piece of sweet potato). Strepsirrhines have sensory adaptations for using olfaction to locate food^53^, while the cercopithecoids are heavily reliant on vision to locate resources^54^, so we ensured that each platform was baited with identical food items within a trial that smelled and looked the same to avoid biasing where the animals chose to go. Platforms for the wild monkeys were not rebaited between trials until all animals were ≥ 20 m away and the entire sequence could be rebaited before their return^15,24–26^.

For all species, we started a trial when the tested individual entered the array and took the reward at a platform. We then recorded each successive platform visit (including revisits to empty platforms) until all rewards had been collected ending the trial. In our analyses, we included a total of 852 trials collected over six navigational experiments, completed by 40 unique individuals (18 lemurs, 12 vervets, 10 macaques) (Table 2).

**Table 2:** Individuals and trial sample size included in the analysis.

Species	Array	# Individuals	# Trials
*C. pygerythrus*	Pentagon	4	110
	Z-Array	9	269
	Double-Trapezoid	3	70
*C. medius*	Double-Trapezoid	6	158
*M. murinus*	Double-Trapezoid	6	88
*D. madagascariensis*	Double-Trapezoid	6	42
*M. fuscata*	Z-Array	10	115
Totals		44	852

#### Data simulations

In addition to empirically collected data, we simulated agents learning to travel efficiently in the same set of arrays using a simple iterative-reinforcement learning model based on the one used by Reynolds et al.^6^ to test for traplining behavior in bumblebees. In this model, agents move randomly between locations in an array until they visit all locations, then reset for another trial. If the agent completed a trial by travelling less distance than on previous trials, the probability of the agent repeating location-to-location transitions that occurred in that trial increased for future trials by a reinforcement factor. Initial transition probabilities were inversely proportional to the distance between two locations. Unlike Reynolds et al.^6^ our simulated agents started at a random location and were not required to return to that location to complete the trial. This matches the trial structure used in our experiments (open-TSP), and reflects multiple central place foraging patterns in primates^55^. Finally, agents could not return to the location they had just come from, using an “avoid the last location” behavioral heuristic observed in nectivores^56,57^, which prevented agents from getting stuck in “loops” between two locations (S1 Simulation Validation).

Within each of the arrays used to collect empirical data, we ran simulations with reinforcement factors of 1 (no reinforcement), 1.2 (mild reinforcement), and 2 (strong reinforcement). For each array and reinforcement factor combination, we ran 100 agents that each completed 120 trials, where there was an equal probability of starting each trial at any location. Then, for each array and reinforcement factor combination, we ran 100 additional simulations per species tested in the given array, where the probability of starting a trial at any location was equal to the empirically observed location-starting probabilities of the respective species.

These simulations were designed to help us test predictions of our two hypotheses regarding primate learning and decision making within the arrays. If primates learn to solve navigational arrays efficiently by reinforcing movements between platform pairs, they should exhibit overall greater receptiveness in their sequences of location visits than reinforcement factor 1 simulations, and a greater decrease over time in total distance travelled to complete the arrays. If primates are pre-disposed to navigate arrays using heuristics, they should exhibit shorter distances travelled on initial trials than in simulations.

### Data analysis

From the raw sequences of locations visited in each trial, we calculated two metrics: minimum distance traveled, and the proportion of platform revisits that occurred within identical 3-platform visit sequences (determinism-DET)^18^. All calculations were done using R version 4.0.4^58^ and packages rstan^59^ and tidyverse^60^. A fully reproducible data notebook containing this work, as well as all analyzed data, is available at https://github.com/aqvining/Do-Primates-Trapline. All figures were created by AQV in R version 4.0.4 and ProCreate.

#### Distance traveled

To calculate minimum distance traveled, we created a distance matrix for each resource array containing the relative linear distance between any two resource locations. These minimum linear distances approximate the distances traveled by the animals, which may not necessarily be linear. We then summed the linear distances for all transitions made in a trial. Because resource arrays were scaled to the subject species’ body size, these relative distances were standardized.

#### Determinism

Given a sequence of observations, Ayers et al.^63^ defines determinism (DET) as the proportion of all matching observation-pairs (recurrences) that occur within matching sub-sequences of observations (repeats) of a given length (minL). This metric has been previously used to distinguish sequences of resource visitation generated by traplining behaviour from sequences generated by known processes of random movement within a given resource array^18,61,62^. It has several advantages in the analysis of foraging patterns, including the ability to detect repeated sequences between non-consecutive foraging bouts, imperfect repeats in sequences (i.e., omission or addition of a particular site), and distinguishing between forward- and reverse-order sequence repeats^63^.

We adapted the methods of^63^ to calculate the number of recurrences and repeats generated by the sequence of location visits in each trial of our experiments and simulations. Based on an analysis of the sensitivity of DET scores to the parameterization of minL, we set minL to three for our calculations (S2 Sensitivity Analysis).

### Statistical analyses

#### Learning rates

We modelled distance travelled as a function of trial number, species, and individual. Metrics of animal performance on learned tasks are known to follow power functions over time and experience^64^, so we a priori applied log transformations to distance travelled and trial number, then fit a linear model. Thus, in the resulting model, the intercept can be interpreted as an estimated distance travelled on the first trial and the slope can be interpreted as the exponent of a learning curve. We modelled species and individual effects on the intercept by summing an estimated grand mean (*µ*_0_), species level deviation (*µ*_sp,j_), and individual level deviation (*µ*_id,i_). We treated species and individual level effects on the learning rate parameter (slope) the same way, summing a grand mean (*b*_0_), species level deviation (*b*_sp,j_), and individual level deviation (*b*_id,i_). We estimated additional parameters for the variance of individual level deviations in intercept and slope (*σ*_µID_ and *σ*_bID_, respectively). Finally, after finding residuals in an initial analysis to have variances predicted by trial number and species, we estimated a separate error variance for each species (*σ*_ε,sp_) and weighted the standard deviations of the resulting error distributions by dividing them by the square root of one plus the trial number.

We set regularizing priors on the model parameters, assuming distances travelled would remain within one order of magnitude of the most efficient route, but not setting any strict boundaries. For the grand mean of the intercept, we used a normal distribution centered around twice the minimum possible distance required to visit all platforms in the array, with a variance of one. For the grand mean of the slope and all species and individual level deviations to the slope and intercept, we used normal distributions centered at zero with variance of one. For all error terms, we used half-cauchy priors with a location parameter of zero and a scale parameter of one. The full, hierarchical definition of the model is given in Eq. (1).

$$Distance \sim {\mu }_{0}+ {\mu }_{sp,j}+ {\mu }_{id, i}+\left({b}_{0}+ {b}_{sp, j}+ {b}_{id,i}\right)Trial+ \epsilon$$

$${\mu }_{0} \sim \mathrm{N}(4.78, 1)$$

$${\mu }_{sp}, {b}_{0}, {b}_{sp} \sim \mathrm{N}(\mathrm{0,1})$$

$${\mu }_{id} \sim \mathrm{N}(0, {\sigma }_{\mu ID})$$

$${b}_{id} \sim \mathrm{N}(0, {\sigma }_{bID})$$

$$\epsilon \sim \mathrm{N}(0, {\sigma }_{\epsilon ,sp}/\sqrt[2]{1+Trial})$$

$${\sigma }_{\mu ID}, {\sigma }_{bID}, {\sigma }_{\epsilon } \sim \mathrm{Half Cauchy}(\mathrm{0,1})$$

#### Determinism

To compare DET between species, and between empirical and simulated data, we created a binomial model of expected repeats generated in a trial given the number of recurrences (Eq. 2).

$$Repeats \sim binom(Recursions, DET)$$

$$DET= {logit}^{-1}(alpha)$$

$$alpha={a}_{0}+Sp+Src+ Int+ID$$

$${a}_{0}, Sp, Src, Int \sim \mathrm{N}(0, 1)$$

$$ID \sim \mathrm{N}(0, {\sigma }_{ID})$$

$${\sigma }_{ID}\sim \mathrm{Half Cauchy}(\mathrm{0,1})$$

where *a*_*0*_ is the mean intercept, *Sp* is one of four coefficients determined by the species (simulations are of the “species” which was used to assign its starting-location probabilities), *Src* is one of four coefficients determined by the source (empirical data and each level of reinforcement factor), Int is one of 16 interaction coefficients (each possible combination of *Sp* and *Src*), and *ID* is a varying effect of the individual. Because the length of a sequence affects DET, we limit our analysis of DET to the sequences generated by a subject’s or an agent’s first ten trials. Subjects that completed fewer than ten trials were excluded from this portion of the analysis.

## Results

### Distance travelled and learning rates

#### Double trapezoid

**Figure 2:**
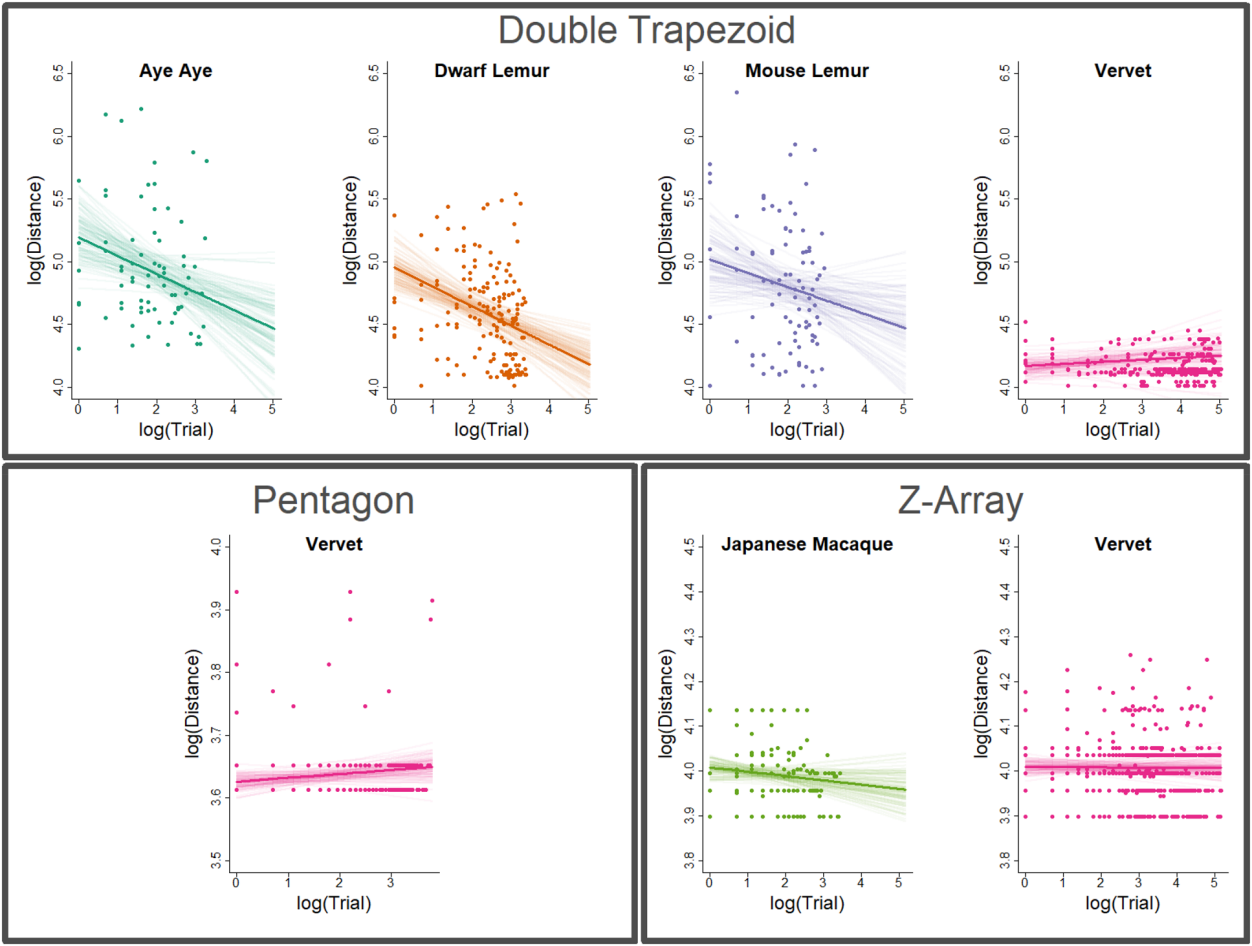
Raw data and model regressions for empirical analyses. The mean linear regression of log minimum distance traveled over log trial number for each species (thick lines), plotted through raw data (points) for all empirical observations. Thin lines represent linear regressions of 100 randomly selected samples from the posterior. All regressions are determined by adding the species-specific deviations for the intercept and slope in a given sample to the estimates of the grand mean for the intercept and slope, respectively. Note that axes are fixed within arrays, but not between.

**Figure 3:**
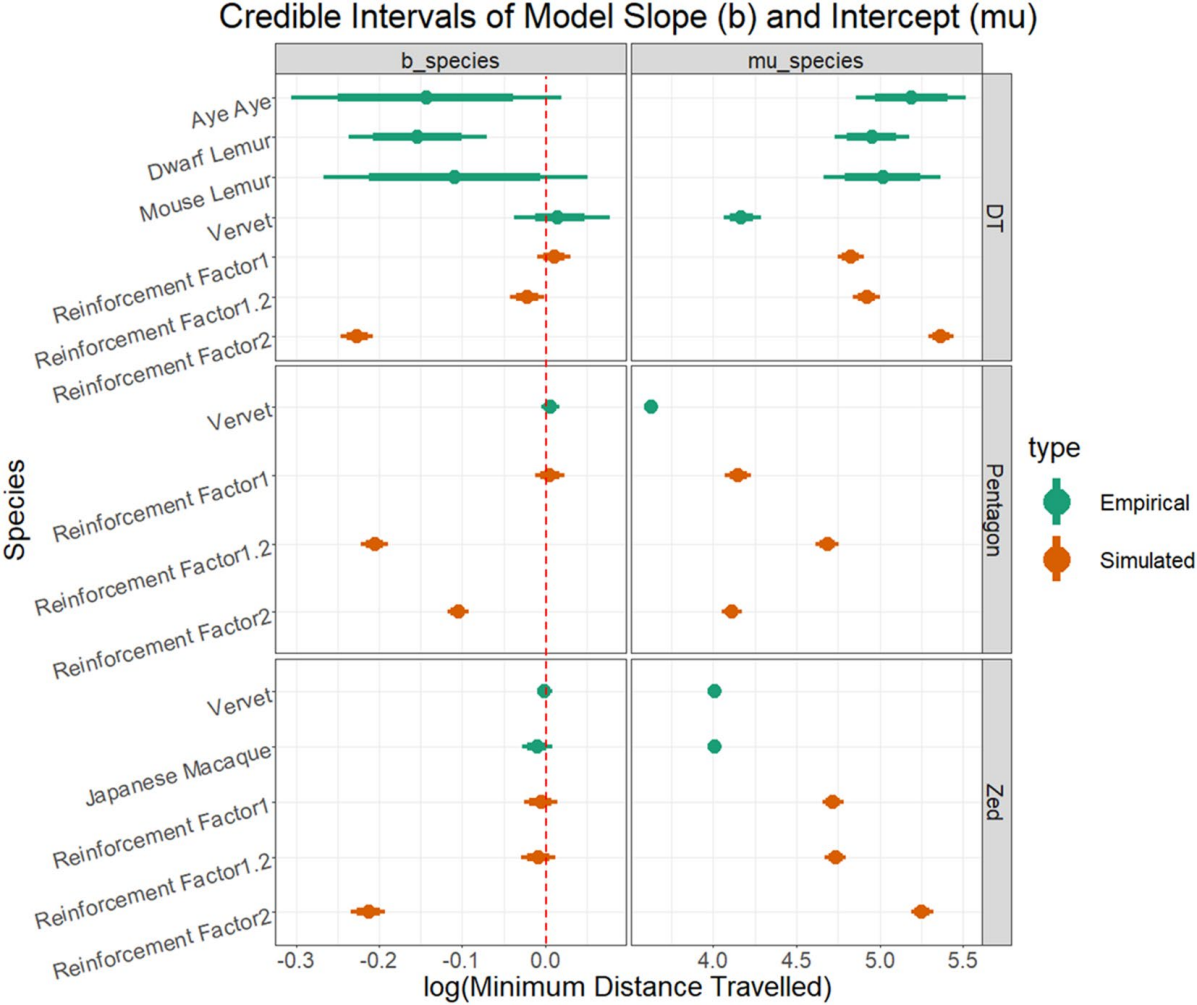
Posterior credible intervals for species-level slope and intercept parameters. 80% intervals (wide bars) and 95% intervals (thin bars) of posterior distributions of the model intercept for all species and simulated agents. The only species that show clear improvement with experience are dwarf lemurs in the Double Trapezoid (credible interval does not contain 0, dashed red line), though mouse lemurs and aye ayes also show some evidence of improvement in this array. Across arrays, vervets and Japanese macaques travel significantly less on initial trials than other species, and less than expected given the distance weighted location transitions of the Reinforcement Factor 1 agents. Created in R version 4.0.4.

In the Double Trapezoid array, the estimated minimum distance travelled on first trial (model intercept) was far lower for vervets (95% Credible Interval (CI): 4.06–4.29) than for the strepsirrhine species. Between the strepsirrhines, the estimated intercept was lowest for dwarf lemurs, then mouse lemurs, and finally aye-ayes (95% CIs: dwarf lemur: 4.73–5.18, mouse lemur: 4.67–5.37, aye-aye: 4.86–5.52). The mean regression and sample regressions from the posterior are plotted through the data for each species and all arrays in Fig. 2. Because there is considerable overlap in the posterior distributions of intercept parameters for strepsirrhine species, the hypothesis that dwarf lemurs travel the least in initial trials is only weakly supported. More than 80% of the posterior distribution for the slope parameter for mouse lemurs falls below zero. This rises to > 90% for aye-ayes, and 100% for dwarf lemurs (Fig. 3). The mean estimated slope parameter for vervets is positive, though 20% of the posterior sample falls below zero. Thus, there is strong evidence that dwarf lemurs and aye-ayes improve their performance with experience and moderate evidence of the same for mouse lemurs, but no evidence for vervets. However, even with improvement over experience, none of the strepsirrhine species reach the initial performance of vervets, nor does our model predict they would until at least 120 trials.

When the same statistical model used to analyze the empirical data was applied to simulated agents using a reinforcement learning algorithm to transition between locations in the same array, the model did not fit as well. Model residuals were highly structured, containing a strong negative relationship with predicted distance travelled and starkly different variances dependant on trial number, suggesting our statistical model poorly describes the improvement dynamics of the iterative reinforcement algorithm. Nonetheless, estimated learning rates for agents of each level of reinforcement factor showed predicted patterns; the “species” level posterior for agents with reinforcement factor of 1 contains 0 (95% CI − 0.009 to 0.031), it is slightly (but entirely) below zero for those with a reinforcement factor of 1.2 (95% CI − 0.042 to − 0.0003), and well below 0 for individuals with a reinforcement factor of 2 (95% CI − 0.246 to − 0.207). Unexpectedly, the estimated intercept (initial performance) is much higher for agents with a reinforcement factor of 2 (95% CI 5.29–5.44) than for those with 1.2 (95% CI 4.84–5.00) or 1 (95% CI 4.75–4.90). Given that all agents (regardless of reinforcement factor) are known to behave identically on the first trial, this result is certainly an artifact of the mismatch between the statistical learning model and the actual dynamics of the reinforcement algorithm. Plotting the regressions through the data reveals a floor effect, where agents lock into one of a few short routes well before trials end, which may contribute to this poor fit. Inspection of individual improvement curves over trial number also point to a cause of this mismatch; individuals with reinforcement factors of 2 show initially flat or even increasing distances travelled, followed by sharp decreases. The timing of this drop-off varies greatly by individual (never even appearing for some) and explains both the high inter-individual variance in estimated model parameters for reinforcement factor 2 agents and the inaccurate intercept parameter estimates. Further analysis and visualization of data from simulations can be found in S3: Data Notebook.

#### Pentagon

Vervets in the Pentagon Array showed results comparable to their performance in the Double Trapezoid. Initial performance started very close to optimal (Fig. 2), with individuals circumnavigating the perimeter of the array on the majority of early trials. This pattern is consistent with a predisposition for a number of route selection heuristics, including nearest-neighbor and convex-hull. In later trials, there was a tendency to select slightly less optimal routes, as indicated by the positive value of ${b}_{0}+ {b}_{vervet}$ in the majority of our posterior samples (Fig. 3). However, with support from only 89% of the posterior sample, this positive trend is insufficient to convincingly rule out the possibility that vervets do not alter their route selection heuristics with experience; instead, less efficient routes in later trials may have occurred by chance.

Simulations in the Pentagon showed a different pattern than in the other arrays. Agents with a reinforcement factor of 1.2 exhibited a strong learning effect, with a posterior distribution of the slope greater in magnitude than agents with reinforcement factor of 2. This can likely be attributed to a highly overestimated intercept, as reinforcement factor 1.2 agents did not achieve short routes as often as reinforcement factor 2 agents.

#### Z-Array

Japanese macaques and vervets in the Z-Array performed similarly on initial trials (species intercept 95% CIs: vervets 3.99–4.03, macaques 3.97–4.04; Fig. 3). While vervets remained consistent in their performance across trials (as in other arrays), Japanese macaques showed slight improvement over trial number, with 86% percent of posterior samples estimating a negative slope. This is not sufficient evidence to rule out the hypothesis that decreased distance travelled in later trials occurred by chance, but is notable given how close the initial performance of Japanese macaques was to optimal. Simulations in the Z-array yielded similar results to the Double Trapezoid, though reinforcement factor 1.2 agents appear slightly less able to find efficient routes in this array.

### Traplining in primates: recursion relative to random transitions in the arrays

**Figure 4:**
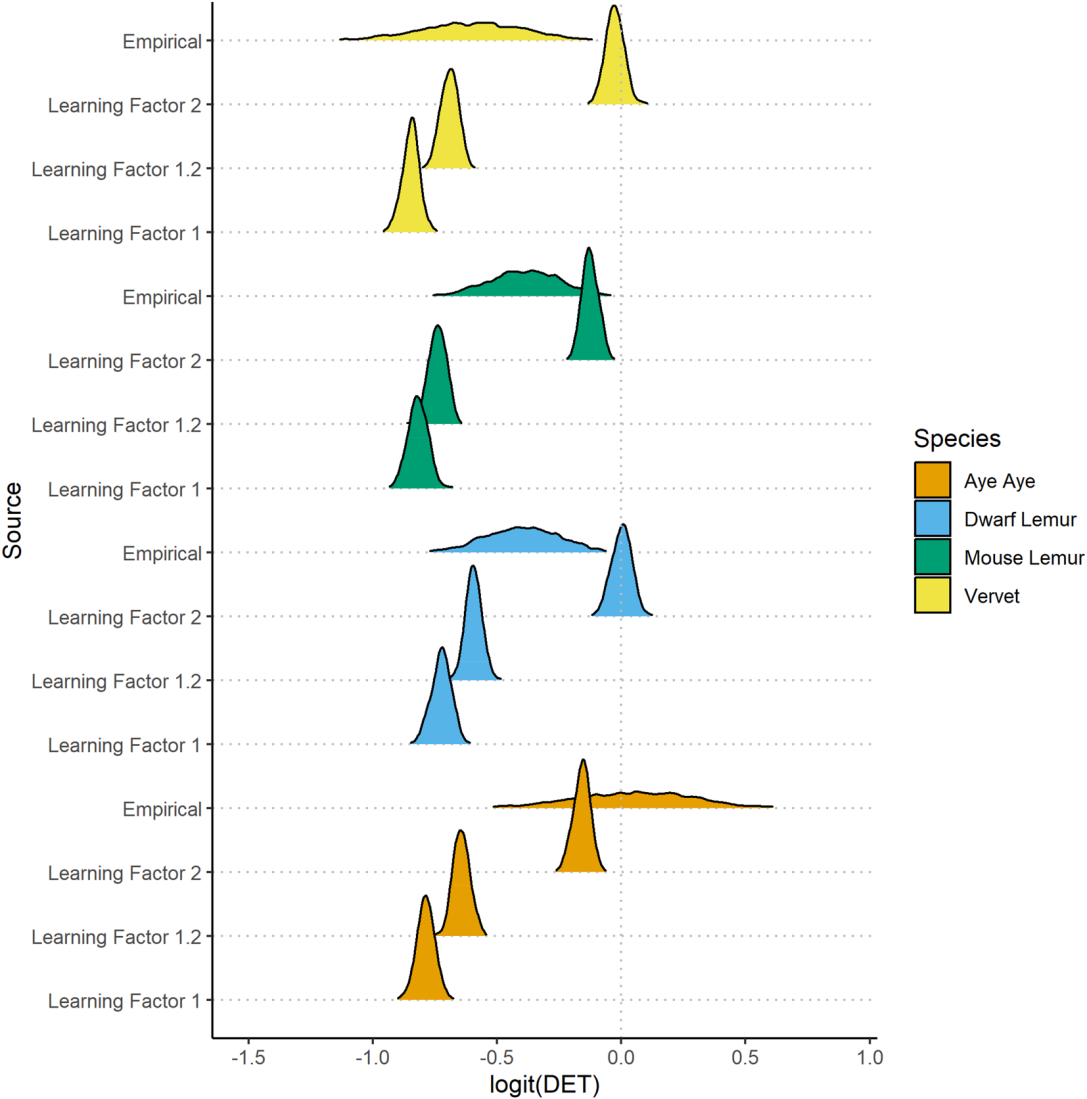
Posterior distributions of alpha estimates. Estimates of alpha (related to DET through a logit link-function) are calculated by summing the mean intercept (*α*_*0*_) with relevant coefficients for species, source, and interaction. Colored plots represent the density of alpha estimates in the posterior for each possible combination of these coefficients, arrayed along the y-axis. The y-axis labels denote the source and plot color denotes the species. The scale of the density axis (height) is not shown, but consistent across all plots.

Posterior estimates of parameters in the binomial model of DET were well mixed (S4 DET Analysis). In our empirical data, estimates of DET in the Double-Trapezoid array were highest for aye ayes and lowest for vervets, but overlap between the posterior distributions of these estimates (Fig. 4) are too large to draw conclusions. As expected, estimates of DET show clear distinctions between simulations with different reinforcement factors, with higher reinforcement factors resulting in higher DET. For the most part, lemurs exhibited higher DET than species-relevant simulations with a reinforcement factor of one, but less DET than species-relevant simulations with a reinforcement factor of 2. The exceptions are vervets, for which about 50% of posterior DET estimates fall within or below the posterior distribution of Learning Factor 1 estimates, and aye ayes, for which more than 50% of the posterior DET estimates fall above the posterior distribution of Learning Factor 2 estimates.

#### Pentagon and Z-array

The posterior distributions of estimated DET scores for vervets in both the pentagon array and the Z-array were entirely above those for simulated reinforcement learning agents (S4 DET Analysis). Japanese macaques, which were only tested in the Z-array, exhibited DET scores in this array lower than those of the vervets. The posterior distribution of estimated DET scores for Japanese macaques in the Z-array fully contained that of reinforcement learning agents with a reinforcement factor of 2 (S4 DET Analysis).

## Discussion

Our results show several key takeaways: (1) Primate movement decisions in our multi-destination arrays were more consistent than would be expected given random transitions with probabilities proportional to distance between targets (i.e. Learning Factor 1); (2) Wild vervets and Japanese macaques, with little to no experience, navigated our arrays with far less travel than captive strepsirrhines and chose paths close to optimal; (3) Captive strepsirrhines exhibit reduced travel distance with more experience, showing improvement rates that are well fit by traditional statistical models of learning rates; (4) These improvement rates correlate with the degree of frugivory in strepsirrhines—being greatest in dwarf lemurs, followed by aye-ayes, and then mouse lemurs. However there is too much uncertainty in our estimates of learning rates to make definitive conclusions; (5) The iterative-reinforcement algorithm proposed by Reynolds et al.^6^ to explain traplining patterns in bumblebees is not sufficient to explain route-finding patterns in the primates we tested, which are generally not central place foragers.

Our hypothesis that primates’ approximate solutions to multi-destination routes by reinforcement learning of location-based decisions was supported most strongly in our strepsirrhine sample. We found strong evidence for improvement with experience in dwarf lemurs and aye-ayes, and notable improvement in mouse lemurs. Vervets finished the Double Trapezoid array faster than the strepsirrhines but the credible intervals for their learning rates contained zero and trended positive (increased distance travelled with experience). Initial performance by vervets was so close to optimal that we may not have observed reinforcement-learning of location-based decisions even if it did occur. Thus, we can rule out the null hypothesis that strepsirrhines do not improve their performance with experience but cannot do the same for vervets. Our data suggest that the improved performance in strepsirrhines results from reinforcement learning of location-based decisions. There are, however, alternative explanations, such as strepsirrhines becoming more motivated as they grew comfortable with the testing environment, thus exploring less and reducing travel distances. Additionally, because the strepsirrhines were raised in captivity, they may simply not have learned spatial heuristics potentially used by their wild counterparts.

Analysis of our simulations also suggests that if strepsirrhines are improving performance through reinforcement learning, this process is not well modeled by the iterative-reinforcement algorithm of Reynolds et al.^6^. At reinforcement factors that produced learning rates comparable to strepsirrhines, this algorithm also yielded very high inter-individual variation in learning rates and learning curves that were not well fit by traditionally used power law functions. Strepsirrhine learning curves were comparably consistent between individuals and well-fit by power law function. Thus, strepsirrhines may use search strategies that balance exploration with the identification and exploitation of efficient navigation strategies. The simulations, conversely, depend on stochastic transitions to find an efficient route and then quickly exploit that route, to the exclusion of other possibilities.

Our second alternative hypothesis—that primates have either innate or previously learned heuristics that can be generalized to approximate solutions to multi-destination routes in many different arrays—was most strongly supported by our cercopithecoid sample. We found that vervets and Japanese macaques did not significantly reduce their distance traveled over time in any array. Their observed distance traveled was significantly lower than in simulations of the other strategies; they immediately started with efficient routes and maintained them. This evidence suggests that vervets and Japanese macaques depended on previously learned or innate spatial heuristics from the beginning of their experience within the arrays and did not deviate from these. This is reinforced by the finding that vervets in the Z-array strongly relied on the nearest-neighbour heuristic, even though it was not consistent with the shortest possible path^12^. Japanese macaques also showed a reliance on heuristics in the Z-array that did not lead to the shortest paths^26^. These findings are not surprising—previous work has found that other primates frequently choose routes that are more efficient than chance but less efficient than optimal^3^. Optimal routes were frequently observed only in the relatively simple Pentagon array, but less frequently in the more complex Z-array or Double Trapezoid array.

Though our learning simulations eventually reached an optimal path in the Pentagon array (within 70–100 trials), the vervets achieved this optimal path often on the first trial—much quicker than our model simulations. Use of either the convex-hull or the nearest-neighbour rule leads to the shortest path. Thus, the simple heuristics that vervets appear to apply in this array are far more efficient than a reinforcement-learning based approach. This suggests that under certain conditions, these “fast and frugal” heuristics are more adaptive and efficient than the cognitively more demanding learning-based alternative^65^.

### Traplining in primates

Within the first 10 trials in the Double Trapezoid Array, individuals from all species exhibited DET values^63^ that were greater than those calculated via simulations with random transitions between targets (Learning Factor 1). This evidence of repeated sequences of site visitation supports previous suggestions that primate food site visitation sequences were reminiscent of traplining^19–23^, though these sequences occur in a less stereotyped manner than well-established traplining animals (e.g., bees). The wide posterior distributions of empirical DET estimates likely reflect both our small sample sizes and the likely possibility that primates vary between trials in their tendency to repeat paths. Additional studies with more animals could effectively model the effects of experience on DET and potentially find narrow estimates that can be more usefully compared to simulations with different learning factors.

The performance of our iterative reinforcement models were also array-dependent. In all arrays, a reinforcement factor of 2 produced strong decreases in distance over trial number, while a lower learning factor of 1.2 produced a strong decrease only in the Pentagon and a minor decrease in the Double Trapezoid. This again reflects the simplicity of the Pentagon array relative to the Double Trapezoid and Z-arrays. Reynolds et al.^6^ also utilized an iterative-reinforcement algorithm to analyze traplining behavior across a variety of arrays, finding fast and convergent learning rates between their algorithm and bumblebees in a pentagon array, and noting the failure of the algorithm in other arrays. By adding a no-backtracking rule to this algorithm, we were able to successfully simulate improvements in array navigation, but these improvements were not well modeled by a power-law learning curve, as often seen in animal learning. In future work, more complex learning models may be able to more accurately reflect the patterns of exploration and improvement that animals use to efficiently exploit resources in environments with complex structure. New ways of measuring patterns of exploration and improvement may complement DET metrics by revealing different aspects of animal decision making.

### Evidence of a phylogenetic signal in navigational behaviour, ecological determinants, or a wild vs. captive difference?

It is intriguing that the navigational behaviours of the strepsirrhines in our dataset were closely aligned, while the cercopithecoids were also similar to one another. This suggests that the cognitive skills underlying navigational strategies in primates have some unknown level of phylogenetic signal, consistent with other behavioural traits (e.g., social organization^66^; activity pattern^67^). However, despite any potential constraints in navigational abilities introduced via evolutionary history, some behavioural variability may still be introduced via specialized adaptations for ecological niches.

The strepsirrhine species we studied all share a distinct behavioural trait that separates them from vervets and macaques—they are primarily solitary in contrast to the highly social cercopithecoids. This is notable because recent work suggests that spatial cognition brain regions are expanded in strepsirrhines and solitary primates, potentially because animals ranging alone require greater spatial memory to find food and mates^68^. Conversely, social species rely on each other for increased accessibility to food, including detection and defense. Social species may benefit more from improved sensory perception instead of enhanced spatial abilities, which would be more useful for distinguishing between resources. Our findings may provide some preliminary support for a trade-off between visual processing centers and spatial abilities in social primates, with reduced spatial learning in cercopithecoids and enhanced spatial learning in strepsirrhines. Future research with wild animals under natural conditions is needed to provide further support for a trade-off between spatial abilities and sensory perceptions in social versus solitary species. However, although the strepsirrhines aligned more closely when compared to the cercopithecoids, we found differences between the strepsirrhines which may be caused by their different ecological niches.

Ecological and dietary variability is great within primates, so it is highly unlikely that most species will rely on the same or similar cognitive mechanisms during navigation. Among the strepsirrhines in our dataset, the most frugivorous species (i.e., dwarf lemurs) had the lowest overall credible intervals for initial travel paths and learning curves. While this pattern fits our prediction that more frugivorous animals would adopt strategies to efficiently navigate arrays, the degree of overlap in parameter estimates for the strepsirrhines prevents us from making any strong conclusions. Although dwarf lemurs align more closely with the other strepsirrhines with regards to initial performance and faster learning, we know from previous work that they do appear to utilize some navigational heuristics more often than either aye-ayes or mouse lemurs^13^, similar to cercopithecoids. Heuristic use in dwarf lemurs is also weakly supported by our finding that they traveled shorter distances initially relative to other strepsirrhines in the same array. Dwarf lemurs thus show some evidence for both reinforcement-based learning of navigational paths and use of heuristics and may be utilizing both strategies. This could be because dwarf lemurs face the added constraint of spending up to seven months at a time in torpor, where they rely on accumulated fat for subsistence^38^. Thus, they have limited time out of torpor to take in resources and build up their reserves, and likely need to do this as efficiently as possible. Other species heavily reliant on replenishing resources have also been reported to use multiple navigational strategies, for example pollinating bees show iterative learning while also linking nearest-neighbour flowers^5^. Our findings for dwarf lemurs suggest that the tendency to combine navigation strategies may be motivated by a diet of spatially stable, replenishing food sources. These findings support our prediction that dwarf lemurs would show shorter distances with experience and increased determinism.

Our findings of similar navigational strategies for strepsirrhines versus cercopithecoids also conform to the different conditions experienced by our sample (i.e., the strepsirrhines were captive and the monkeys were wild). Many variables could potentially alter experimental outcomes, such as internal factors that influence motivation^69^. Wild monkeys were tested opportunistically at different times during the day and some individuals ran more trials consecutively than others, which may have led to satiation and performance fatigue. We also cannot consider unknown variables such as complete diet and health status. For example, macaques were provision fed whereas vervets were not. Provision feeding in particular may encourage repeated sequences of visits to baited sites. Further, wild animals experienced distractions including alarm calls, vigilance for predators, and changes in weather conditions. The strepsirrhines in our sample were all raised in captivity; environments much smaller than their natural territories that lack the ecological pressures they evolved with. This may have kept them from learning heuristics they might otherwise use^70^. These species are also all nocturnal and may face different perceptual constraints. We cannot be certain of the effect that these or other variables may have had on our results. Exploring whether captive cercopithecoids also exhibit first-trial heuristic use in multi-destination routes is an important future direction. Likewise, investigating a larger sample that includes species with overlapping behavioural traits, such as social strepsirrhines (e.g., ring-tailed lemurs), or strepsirrhines with varying activity patterns (e.g., diurnal or cathemeral), and comparing with our results would be useful future studies.

### Ethics

This work was approved by the Uganda Wildlife Authority (UWA/COD/96/02), the Uganda National Council for Science and Technology (NS 537), the University of Toronto Animal Care Committee (#20011416), the Duke Institution Animal Care and Use Committee (A290-14-12), and the Concordia University Animal Research Ethics Committee (#30009663). These experiments were conducted according to ARRIVE guidelines and were performed in accordance with all institutional and/or national guidelines and regulations.

## Supplementary Information

Supplementary Information 1.

Supplementary Information 2.

Supplementary Information 3.

Supplementary Information 4.

## Data Availability

The raw data are included in the manuscript via a public link: https://github.com/aqvining/Do-Primates-Trapline.
