# Supplementary material for: Mild movement sequence repetition in five primate species and evidence for a taxonomic divide in cognitive mechanisms
Source: Sci Rep. 2022 Aug 25;12:14503. doi: 10.1038/s41598-022-18633-7 (PMC9411198; doi:10.1038/s41598-022-18633-7)
Supplement: Supplementary file 1 — Supplementary Information 1. [file 41598_2022_18633_MOESM1_ESM.html]

Simulation Validation


# Simulation Validation

#### Alexander Vining

#### 2022-06-11

# Additional Simulations

In our initial simulations of transitions through the three platform
arrays using a reinforcement learning algorithm, we made some
modifications from the algorithm used by Reynolds et al. 2013. This was
done because in our lemur experiments, there was no central nest from
which lemurs started and to which they had to return. This created a
challenge for the original algorithm, whereby transitions back and forth
between two locations could become reinforced. We thus implemented a no
backtracking rule, but even so, efficient routes did not emerge in all
arrays. Though this is consistent with the findings of Reynolds et
al. 2013, here we more precisely replicate the algorithm and context of
Reynolds et al. to ensure our results are truly due to the different
context on the environment, and not differences between our alogirthm
and the one used by Reynolds et al.

To do this, we first add a central start-end location to the pentagon
array (akin to Lihoureau et all 2011, simulation results described in
Reynolds et al. 2013) and to the double trapezoid array (akin to to
Saleh and Chitka 2007, simulations results not described by Reynolds et
al 2013). In our initial simulations without a central “nest”, our
reinforcement algorithm often failed to find a stable, efficient
route.

```
Pentagon_central <- rbind(Pentagon, st_sf(NAME = "N", geom = st_sfc(st_point(c(0,-10)))))
Double_Trapezoid_central <- rbind(Double_Trapezoid, st_sf(NAME = "N", geom = st_sfc(st_point(c(-1,12)))))
resource_arrays <- list(Pentagon_central, Double_Trapezoid_central)
names(resource_arrays) <- c("Pentagon_central", "Double_Trapezoid_central")
#starts = rep("N", times = 120) #120 trials is hard-coded into the exective funtion for running learning simulations
```

We then make minor changes to the functions used to simulate the
learning algorithm to force all simulations to start and end at the
added central (nest) locations and to allow backtracking.

```
runIterativeLearningSimulations <- function(emperical_data, resource_arrays, starts, exclude_backtrack = TRUE, runs = 1000){
  #purpose: Parameterize and run all iterative learning simulations
  #input: emperical_data; data_frame with empirical observations
  #       resource_arrays: named list containing spatial features dataframes
  simulated_data <- data.frame()
  for(array in names(resource_arrays)) {
    for (learning_factor in c(1,1.2,2)) {
      print(paste("running ", array, " with learning factor", learning_factor))
      for (i in 1:runs) {
        if (i %% 50 == 0) print(paste("run ",i, " of ", runs))
        run_data <- iterativeLearningSimulation(resource_arrays[array], learning_factor, trials = 120, starts = NA, exclude_backtrack = exclude_backtrack)
        run_data$ID <- paste(i)
        simulated_data <- rbind(simulated_data, run_data)
      }
    }
  }
  return(simulated_data)
}

#in the learner agent, we set the start to be at "N"and we change the end condition to be a return to "N", as in Reynolds et al. 2013
Learner <- setRefClass("Learner", 
                       field = list(sequence = "character", transition_matrix = "matrix", learning_factor = "numeric", min_distance_trial = "numeric"),
                       method = list(initialize = function(..., sequence = c(), transition_matrix = array(data = NA, dim = 2),learning_factor = 1.2, min_distance_trial = NA) {
                         callSuper(..., sequence = sequence, transition_matrix = transition_matrix, learning_factor = learning_factor, min_distance_trial = min_distance_trial)
                         },
                         runTrial = function(exclude_backtrack = TRUE) { #generates a sequence of visits from transition matrix, ending when all locations have been visited
                           sequence <<- "N" #set starting location
                           while(length(sequence) <= 2 | sequence[length(sequence)] != "N") { #change from original, see description in comment above
                             if(exclude_backtrack & length(sequence) >= 2){ previous_location <- sequence[length(sequence)-1]
                                new_transition_matrix <- transition_matrix[,!colnames(transition_matrix) == previous_location]
                                sequence <<- c(sequence, sample(colnames(new_transition_matrix), 1, prob = new_transition_matrix[sequence[length(sequence)],]))
                             } else sequence <<- c(sequence, sample(colnames(transition_matrix), 1, prob = transition_matrix[sequence[length(sequence)],])) #get new location by sampling row of transition matrix determined by last location visited, add new location
                             if(length(sequence) > 50) return()
                           }
                         },
                         updateTransitions = function(sequence_path_length) {
                           min_distance_trial <<- sequence_path_length
                           transitions <- unique(paste0(head(sequence, -1), sequence[-1])) #gets transitions as a vector of ordered pair characters
                           for (transition in transitions) {
                             transition <- strsplit(transition, NULL)[[1]]
                             transition_matrix[transition[1], transition[2]] <<- transition_matrix[transition[1],transition[2]] * learning_factor #increase likelihood of transitions made
                             transition_matrix <<- t(apply(transition_matrix, MARGIN = 1, function(X) X/(sum(X)))) #normalize
                           }
                         })
                       )

#we remove the addition of a "start" position from the distance transition matrix
getDistanceWeightedTransitions <- function(resource_array, starts) {
  #purpose: generate a transition matrix based on distances between resource arrays, with likelihood proportional to 1/d^2
  #input: resource_array; a spatial features data frame with spatial points in the geometry column and NAME feature OR a list of such (only the first element will be used)
  #output: numeric array
  if("list" %in% class(resource_array)) resource_array <- resource_array[[1]]
  distanceMatrix <- getLabelledDistanceMatrix(resource_array) #from Array_Processing.R
  distanceMatrix <- apply(distanceMatrix, MARGIN = c(1,2), FUN = function(X) ifelse(X==0, 0, 1/(X^2))) #transitions proportional to 1/distance^2
  transition_matrix <- t(apply(distanceMatrix, MARGIN = 1, function(X) X/(sum(X)))) #normalize, transposition necessary as apply output is by column"
  return(transition_matrix)
}

#finally, we add a condition to the reinforcement step that all stations must be visited
iterativeLearningSimulation <- function(resource_array, learning_factor, trials, exclude_backtrack = FALSE, starts = "NA") {
  #purpose: Run a set of trials in which a foraging agent moves between locations in a resource array, minimizing total length traveled by increasing the probability of transitions in a trial that resulted in the shortest path traveled
  #input: resource_arrays; a named list of length one containing a spatial features dataframe with points in the geometry column and a feature NAME
  #       learning_factor; a numeric giving the factor to multiply transition probabilities by upon completion of a shortest-length-traveled trial
  #       trials: the number of trials (full array visits) to simulate
  #       starts: a named vector containing the probability that each element name will be the first location visited. if NULL, starting locations will be selected at random from resource_array$NAME
  #output: a dataframe with colums for Array (character), Species (character, "itLearner_"), Sequence (character), Session (numeric, NA), Trial (numeric), and Distance (Numeric)
  learner1 <- Learner(sequence = "N", transition_matrix = getDistanceWeightedTransitions(resource_array, starts), learning_factor = learning_factor, min_distance_trial = 1000)
  data <- data.frame(Array = names(resource_array), Species = paste("itLearner_", learning_factor), Sequence = NA, Session = NA, Trial = 1:trials, Distance = NA)
  for(i in 1:trials) {
    learner1$runTrial(exclude_backtrack)
    data$Sequence[i] <- paste0(learner1$sequence, collapse = "")
    data$Distance[i] <- getSequenceDistance(learner1$sequence, resource_array)
    #if(learner1$min_distance_trial == 0) learner1$updateTransitions(data$Distance[i])
    if(all(colnames(learner1$transition_matrix) %in% learner1$sequence) & data$Distance[i] <= learner1$min_distance_trial) learner1$updateTransitions(data$Distance[i])
  }
  return(data)
}
```

With setup complete, we can run all of the simulations

```
central_nest_simulations <- runIterativeLearningSimulations(emperical_data = NA, resource_arrays = resource_arrays, exclude_backtrack = FALSE, starts = NA)
#unlike in our main analysis, these simulations complete when the agent returns to N, not when all locations are visited. We add a column to the dataframe to indicate when all resources are visited

checkComplete <- function(Sequence, Array){
  stations = list(as.character(1:5), as.character(1:6))
  names(stations) <- c("Pentagon_central", "Double_Trapezoid_central")
  complete = vector(length = length(Sequence))
  for (i in seq_along(Sequence)) {
    complete[i] <- all(stations[[Array[i]]] %in%  strsplit(Sequence[i], split = '')[[1]])
  }
  return(complete)
}
central_nest_simulations <- central_nest_simulations %>% mutate(complete = checkComplete(Sequence, Array))

write.csv(central_nest_simulations, "../Results/central_nest_simulations.csv")
```

## Simulation Analysis

```
## `summarise()` has grouped output by 'Array', 'Species'. You can override using the `.groups` argument.
```

We can see from these plots that at a reinforcement factor of 2,
agents quickly learn to to complete and optimize travel paths in the
pentagon array, as seen in Reynolds 2013. In the Double Trapezoid array,
reinforcement factor 2 agents do begin completed arrays more often over
time, but they actually increase the distance travelled in these
completed trials. We scanned through some of the sequences in the data,
and under these conditions there appears to be many sequences where the
agent travels back and forth between two locations. This indicates too
high a reinforcement factor without backtracking controls can lead to
transition matrices that reinforce both directions of travel and get
stuck with sub-obtimal
