# Supplementary material for: Mild movement sequence repetition in five primate species and evidence for a taxonomic divide in cognitive mechanisms
Source: Sci Rep. 2022 Aug 25;12:14503. doi: 10.1038/s41598-022-18633-7 (PMC9411198; doi:10.1038/s41598-022-18633-7)
Supplement: Supplementary file 2 — Supplementary Information 2. [file 41598_2022_18633_MOESM2_ESM.html]

Primate DET Report


# Primate DET Report

#### Alexander Vining

#### 8/10/2020

The report details two analyses. The first is an attempt to reproduce the results of Ayers et al. 2018 simulations in NetLogo using my own simulation in R. The reason for this is that by creating my own code, I am able to produce outputs that are easier to use and more flexibly manipulate parameters that the NetLogo simulation was not designed for. My model can also serve as the basis for many additional models that may be outside the scope of NetLogo. However, I need to be sure mine works the same as Ayer’s 2020 before I use it draw conclusions. I describe the simulations conducted below, but have not included the raw simulation code in this report, only the statistical analysis.

Second, I used the model I created and validated to explore how resource selection type affects DET at different levels of sight range when patches are arrayed in the “Double Trapezoid” configuration.

```
setwd("~/Manuscripts/Traplining Vervets/Simulations")
sensitivityResults <- read.csv("AyersSensitivityReplication.csv")
load("sensitivityAnalysisSequences.RData")
```

The data loaded above were generated by running 100 simulations for each combination of parameter levels described below:

abundance (10, 20, 30): Describes the total number of patch locations in the simulation. -Patch locations were determined by sampling from random uniform distributions between -10 and 10 in both the x and y dimensions

sight (10,5,1): The distance at which foragers can detect patches. -If a forager does not already have a target, it will select a target from all patches within its sight distance and move directly toward that target until it arrives.

CRW (0,0.3,0.9): The concentration parameter of the wrapped cauchy distribution from which turning deviations are drawn if a forager is not moving toward a target. -At 0, a foragers bearing is independant of its bearing in the previous time-step -at 1, a foragers bearing is identical to its bearing in the previous time step

distanceForagers (TRUE, FALSE): When TRUE, foragers are less likely to select visible patches that are farther away. When FALSE, foragers are equally likely to target any visible patch. -distance discounting in the TRUE condition is cubic

As in Ayers et al 2018, Foragers would not return to the last two patches visited. The data is checked for format consistency below. Some sample plots of simulated enviornments and movements are in the same folder as this document. DET of each simulated sequences of patch visitations is calclulated using minimum length of repeated sequences as 6 (as reported in Ayers et al.)

```
#Investigate NAs
sensitivityResults[which(is.na(sensitivityResults$DET)),] #initially a check I put in the determinism function was creating NA's. I removed this line of code and no NAs were produced
```

```
## [1] X                DET              abundance        sight           
## [5] CRW              distanceForagers
## <0 rows> (or 0-length row.names)
```

```
#check sequence lengths
summary(sapply(sequences, length)) #should all be 80, the length of sequences analyzed in Ayers et al 2018
```

```
##    Min. 1st Qu.  Median    Mean 3rd Qu.    Max. 
##      80      80      80      80      80      80
```

Data Visualization: Main Effects

Some important things to note here. First, these values are very low. Ayers et al report using a minL of 6, but this creates a significan floor effect and would seem a poor choice. Later plots in their supplementary materials suggest this is in error, and reported values were actually calculated at miniumum length of 3 or 4.

I also wanted to see why there were outliers in the data, so I looked at plots from simulations with high DET.

```
#Explore Outliers
which(sensitivityResults$DET > 0.4) #look at saved plots
```

```
##  [1]  206  663  924 1090 1112 1167 1169 1170 1183 1186 2665 2668 2863 2971
```

```
sensitivityResults[which(sensitivityResults$DET > 0.4),] #parameters at which outlier simulations were created
```

```
##         X       DET abundance sight CRW distanceForagers
## 206   206 0.4016393        10    10 0.3                1
## 663   663 0.5594595        10     5 0.0                1
## 924   924 0.5364238        10     5 0.3                0
## 1090 1090 0.6842105        10     5 0.9                1
## 1112 1112 0.4044586        10     5 0.9                0
## 1167 1167 0.4812500        10     5 0.9                0
## 1169 1169 0.7023411        10     5 0.9                0
## 1170 1170 0.5882353        10     5 0.9                0
## 1183 1183 0.4807692        10     5 0.9                0
## 1186 1186 0.4761905        10     5 0.9                0
## 2665 2665 0.4026549        20     5 0.3                1
## 2668 2668 0.5355191        20     5 0.3                1
## 2863 2863 0.4129794        20     5 0.9                1
## 2971 2971 0.4444444        20     5 0.9                0
```

These plots suggest high DET values are the result of patch arrangements in which foragers essentially get stuck in a subset of patches, reducing the overall set of possible options. it happens most often at low abundance and middling sight range, when random clusters may all be within sight range of each other, but out of sight of any other patches.

Given how low most DET scores are, I tried recalculating DET at a lower minL

```
#Try DET at different minL
sensitivityResults$DET_minl3 <- sapply(sequences, determinism, minl = 3)
ggSensitivity <- ggplot(sensitivityResults %>% filter(distanceForagers == FALSE))
ggSensitivity + geom_boxplot(aes(x = factor(abundance), y = DET_minl3)) + theme_classic()
```

```
ggSensitivity + geom_boxplot(aes(x = factor(sight), y = DET_minl3)) + theme_classic()
```

```
ggSensitivity + geom_boxplot(aes(x = factor(CRW), y = DET_minl3)) + theme_classic()
```

These values look much easier to work with, and much closer to the DET’s seen in Ayers et al’s supp material 1

I used the DETs calculated at both minimum length values to look at the parameters interactions described in Ayers et al supp material 1

```
#visualize means and interactions
sensitivitySummary <- sensitivityResults %>% filter(distanceForagers == FALSE) %>% group_by(abundance, sight) %>% summarize(mean = mean(DET, na.rm = TRUE), Error = sqrt(var(DET, na.rm = TRUE)/length(DET)))
ggplot(sensitivitySummary, aes(x = abundance, y = mean, color = factor(sight))) + geom_line(aes(group = sight)) + 
  geom_point() + geom_errorbar(aes(ymin = mean - Error, ymax = mean + Error), width = 0.1) + theme_classic()
```

```
CRWSummary <- sensitivityResults %>% filter(distanceForagers == FALSE) %>% group_by(abundance, sight, CRW) %>% summarize(mean = mean(DET, na.rm = TRUE), Error = sqrt(var(DET, na.rm = TRUE)/length(DET)))
ggplot(CRWSummary, aes(x = abundance, y = mean, color = factor(CRW))) + geom_line(aes(group = factor(CRW))) + facet_wrap(~sight) +
  geom_point() + geom_errorbar(aes(ymin = mean - Error, ymax = mean + Error), width = 0.5) + theme_classic() + scale_color_brewer(type = "seq", palette = "RdPu")
```

As you can see, the DET values are MUCH lower than those seen in the same figure in the Ayers supplement. With values this low, there is likely to be serious floor effect, where potential effects are missed because everything is just too close to 0. The figure for DET with minL three looks much more like the one in Ayers, and makes for analyzable data.

```
sensitivitySummaryMinL3 <- sensitivityResults %>% filter(distanceForagers == FALSE) %>% group_by(abundance, sight) %>% summarize(mean = mean(DET_minl3, na.rm = TRUE), Error = sqrt(var(DET_minl3, na.rm = TRUE)/length(DET_minl3)))
ggplot(sensitivitySummaryMinL3, aes(x = abundance, y = mean, color = factor(sight))) + geom_line(aes(group = sight)) + 
  geom_point() + geom_errorbar(aes(ymin = mean - Error, ymax = mean + Error), width = 0.1) + theme_classic()
```

```
CRWSummaryMinL3 <- sensitivityResults %>% filter(distanceForagers == FALSE) %>% group_by(abundance, sight, CRW) %>% summarize(mean = mean(DET_minl3, na.rm = TRUE), Error = sqrt(var(DET_minl3, na.rm = TRUE)/length(DET_minl3)))
ggplot(CRWSummaryMinL3, aes(x = abundance, y = mean, color = factor(CRW))) + geom_line(aes(group = factor(CRW))) + facet_wrap(~sight) +
  geom_point() + geom_errorbar(aes(ymin = mean - Error, ymax = mean + Error), width = 0.5) + theme_classic() + scale_color_brewer(type = "seq", palette = "RdPu")
```

These still don’t look exactly the same as the Ayers data, but they are much closer. The only noticeable difference is the higer DET scores with a sight level of 1 in my simulations. I think the most likely explanation for this is field size. I was unable to determine exaclty how big the environment was for the Ayers simulations. Because resource abundance is given as an absolute value, but it is really the resource density that is affecting DET with respect to sight range, we would expect the different environment sizes to effectively shift the x axis. It is probably worth reaching out to Ayers to clarify this.

Next, I checked the linear models tested in Ayers to see if the results were the same.

```
modelminL6 <- glm(DET ~ abundance * sight * factor(CRW), data = filter(sensitivityResults, distanceForagers == FALSE, !CRW == 0.3))
summary(modelminL6)
```

```
## 
## Call:
## glm(formula = DET ~ abundance * sight * factor(CRW), data = filter(sensitivityResults, 
##     distanceForagers == FALSE, !CRW == 0.3))
## 
## Deviance Residuals: 
##      Min        1Q    Median        3Q       Max  
## -0.03676  -0.01734  -0.00871  -0.00108   0.66871  
## 
## Coefficients:
##                                  Estimate Std. Error t value Pr(>|t|)  
## (Intercept)                     1.848e-02  7.598e-03   2.432   0.0151 *
## abundance                      -3.794e-04  3.517e-04  -1.079   0.2809  
## sight                           5.488e-04  1.172e-03   0.468   0.6398  
## factor(CRW)0.9                  2.508e-02  1.075e-02   2.335   0.0197 *
## abundance:sight                -3.834e-05  5.427e-05  -0.706   0.4801  
## abundance:factor(CRW)0.9       -9.273e-04  4.974e-04  -1.864   0.0625 .
## sight:factor(CRW)0.9            7.139e-04  1.658e-03   0.431   0.6668  
## abundance:sight:factor(CRW)0.9 -2.524e-05  7.675e-05  -0.329   0.7423  
## ---
## Signif. codes:  0 '***' 0.001 '**' 0.01 '*' 0.05 '.' 0.1 ' ' 1
## 
## (Dispersion parameter for gaussian family taken to be 0.002395563)
## 
##     Null deviance: 4.5072  on 1799  degrees of freedom
## Residual deviance: 4.2928  on 1792  degrees of freedom
## AIC: -5743.3
## 
## Number of Fisher Scoring iterations: 2
```

```
modelminL3 <- glm(DET_minl3 ~ abundance * sight * factor(CRW), data = filter(sensitivityResults, distanceForagers == FALSE, !CRW == 0.3))
summary(modelminL3)
```

```
## 
## Call:
## glm(formula = DET_minl3 ~ abundance * sight * factor(CRW), data = filter(sensitivityResults, 
##     distanceForagers == FALSE, !CRW == 0.3))
## 
## Deviance Residuals: 
##      Min        1Q    Median        3Q       Max  
## -0.39753  -0.17615  -0.04025   0.09030   0.81184  
## 
## Coefficients:
##                                  Estimate Std. Error t value Pr(>|t|)    
## (Intercept)                     0.5622706  0.0363337  15.475  < 2e-16 ***
## abundance                      -0.0018775  0.0016819  -1.116  0.26446    
## sight                           0.0032819  0.0056064   0.585  0.55836    
## factor(CRW)0.9                 -0.2093629  0.0513837  -4.075 4.81e-05 ***
## abundance:sight                -0.0017750  0.0002595  -6.840 1.09e-11 ***
## abundance:factor(CRW)0.9        0.0014913  0.0023786   0.627  0.53075    
## sight:factor(CRW)0.9            0.0260422  0.0079287   3.285  0.00104 ** 
## abundance:sight:factor(CRW)0.9 -0.0002235  0.0003670  -0.609  0.54261    
## ---
## Signif. codes:  0 '***' 0.001 '**' 0.01 '*' 0.05 '.' 0.1 ' ' 1
## 
## (Dispersion parameter for gaussian family taken to be 0.05478133)
## 
##     Null deviance: 134.939  on 1799  degrees of freedom
## Residual deviance:  98.168  on 1792  degrees of freedom
## AIC: -109.77
## 
## Number of Fisher Scoring iterations: 2
```

Unlike Ayers et al., I did not find significant main effects of abundance or sight level. This is somewhat surprsing given the visualization of those data, perhaps the effect was obscured by the non-linear trend (ie DET goes up in the middle level of abundance). Ayers et al also did more simulations than I did, and so would have more power in their tests. I did find the same significant interactions. I would like to know more about how Ayers fit their model; they report using an error distribution I am not familiar and other model-fitting decisions may further shape these differences.

I also checked whether the patch selection methods I implemented had the same effects on DET as those by Ayers et al. Recall, One method selects from patchs within sight with equal probability, while the other discounts each probability relative to the cubed distances to each patch

```
choiceSummary <- sensitivityResults %>% group_by(abundance, sight, distanceForagers) %>% summarize(mean = mean(DET, na.rm = TRUE), Error = sqrt(var(DET, na.rm = TRUE)/length(DET)))
ggplot(choiceSummary, aes(x = abundance, y = mean, color = factor(sight))) + geom_line(aes(group = factor(sight))) + facet_wrap(~distanceForagers) +
  geom_point() + geom_errorbar(aes(ymin = mean - Error, ymax = mean + Error), width = 0.5) + theme_classic()
```

```
choiceSummaryMinl3 <- sensitivityResults %>% group_by(abundance, sight, distanceForagers) %>% summarize(mean = mean(DET_minl3, na.rm = TRUE), Error = sqrt(var(DET_minl3, na.rm = TRUE)/length(DET_minl3)))
ggplot(choiceSummaryMinl3, aes(x = abundance, y = mean, color = factor(sight))) + geom_line(aes(group = factor(sight))) + facet_wrap(~distanceForagers) +
  geom_point() + geom_errorbar(aes(ymin = mean - Error, ymax = mean + Error), width = 0.5) + theme_classic()
```

These generally matched (again for minl of 3), so I went on to explore how sight distance and patch selection method affected DET of simulated paths in the Double Trapezoid Array. I also wanted to look at how the avoidance of recently visited platforms shapes DET, hypothesizing that a simple rule of “avoid recently visited patlforms” that extends beyond the last two would increase DET

```
setwd("~/Manuscripts/Traplining Vervets/Simulations/DT")
setwd("~/Manuscripts/Traplining Vervets/Simulations/DT")
load("DTsimulationSequences.RData")
DTSimulationResults <- read.csv("DTsimulationResults.csv")
ggplot(DTSimulationResults, aes(x = sight, y = DET3, color = factor(AvoidLength))) + geom_point(aes(group = AvoidLength)) + facet_wrap(~distanceForager) + theme_classic()
```

```
ggplot(DTSimulationResults, aes(x = sight, y = DET6, color = factor(AvoidLength))) + geom_point(aes(group = AvoidLength)) + facet_wrap(~distanceForager) + theme_classic()
```

```
modelDT <- glm(DET3 ~ sight * distanceForager * AvoidLength, data = DTSimulationResults)
summary(modelDT)
```

```
## 
## Call:
## glm(formula = DET3 ~ sight * distanceForager * AvoidLength, data = DTSimulationResults)
## 
## Deviance Residuals: 
##      Min        1Q    Median        3Q       Max  
## -0.29336  -0.07189  -0.01187   0.06176   0.43923  
## 
## Coefficients:
##                                     Estimate Std. Error t value Pr(>|t|)    
## (Intercept)                        0.7333537  0.0277967  26.383  < 2e-16 ***
## sight                             -0.0138642  0.0017283  -8.022 1.76e-15 ***
## distanceForager                   -0.0807373  0.0393105  -2.054   0.0401 *  
## AvoidLength                       -0.0433591  0.0065518  -6.618 4.66e-11 ***
## sight:distanceForager              0.0141739  0.0024442   5.799 7.74e-09 ***
## sight:AvoidLength                  0.0007912  0.0004074   1.942   0.0522 .  
## distanceForager:AvoidLength        0.0054293  0.0092656   0.586   0.5580    
## sight:distanceForager:AvoidLength -0.0012220  0.0005761  -2.121   0.0340 *  
## ---
## Signif. codes:  0 '***' 0.001 '**' 0.01 '*' 0.05 '.' 0.1 ' ' 1
## 
## (Dispersion parameter for gaussian family taken to be 0.01122976)
## 
##     Null deviance: 35.669  on 2009  degrees of freedom
## Residual deviance: 22.482  on 2002  degrees of freedom
## AIC: -3309.2
## 
## Number of Fisher Scoring iterations: 2
```

Using minl of 3, we see significant main effects of all three parameters as well as the interaction of sight by selection method and the three way interaction. These effects are further visualized below.

```
ggplot(DTSimulationResults, aes(x = factor(AvoidLength), y = DET3)) + geom_boxplot() + theme_classic()
```

```
ggplot(DTSimulationResults, aes(x = factor(distanceForager), y = DET3)) + geom_boxplot() + theme_classic()
```

```
ggplot(DTSimulationResults, aes(x = sight, y = DET3)) + geom_point() + theme_classic() + scale_x_continuous(breaks = seq(5, 25, by = 5))
```

```
ggplot(DTSimulationResults, aes(x = sight, y = DET3, color = factor(distanceForager))) + geom_point() + theme_classic()
```

Two interesting things jump out at me here.

First, I noticed the DET actually goes down if the foragers are avoiding more of the recently visited platforms. This was not intuitive to me at first, but I think the effect may be caused because longer “memory” for visited platforms means where you have been is more likely to influence your decision. Thus, the decision an animal makes from a platform if it is the second platform visited will be different than if it is the sixth. If this is the case, we would expect this effect to dissapear at higher DET.

```
ggplot(DTSimulationResults, aes(x = factor(AvoidLength), y = DET6)) + geom_boxplot() + theme_classic()
```

Indeed, at DET 6, the effect is opposite!! This is a very interesting interaction, and one that may be worth going into a bit on the paper.

The second interesting result from these simulations is the difference in DET between random selection foragers and distance weighting foragers at high sight ranges. I think this effect is quite intuitive: random foragers with high sight are going to be equally likely to pick from many disant patches, while distance discounting foragers may still see those patches, but rarely select them. This does, however, reflect an interesting property of these simulations that should be of concern to us. By expanding the sight range to include all options, we are removing random movement element that the simulations were designed for. Essentially, we could replicate these results by simply generating sequences from a transition matrix (one in which all transitions are equally likely, and the other in which they are equal to the proportion of cubed distances.) The point of the Ayers simulations was to show that the bees patch sequences are more predictable than expected from random movement; because the patches are too far apart to detect, they must be using memory to do this. Hence, traplining. Under the assumption that a forager CAN see all the options, the random movement properties are obviated, and what we show instead is that primates are more consistent in their choices than expected by random selection (or as I will show in a moment, distance weighted random selection). That is interesting, but I don’t think it is traplining. I discuss further in my summary (email).

This last point aside, since our interest is primarily at high sight levels, I ran a final set of 1000 simulations that held all parameters constant (sight = 25, repeat avoid = 2, speed = 4) except the patch selection method (500 simulations each)

```
setwd("~/Manuscripts/Traplining Vervets/Simulations/DT High Sight")
DTSimHighSight <- read.csv("DTSimHighSight.csv")
ggplot(DTSimHighSight, aes(x = distanceForager, y = DET3)) + geom_boxplot() + theme_classic()
```

```
ggplot(DTSimHighSight, aes(x = distanceForager, y = DET6)) + geom_boxplot() + theme_classic()
```

```
modelDT3 <- glm(DET3 ~ distanceForager, data = DTSimHighSight)
summary(modelDT3)
```

```
## 
## Call:
## glm(formula = DET3 ~ distanceForager, data = DTSimHighSight)
## 
## Deviance Residuals: 
##      Min        1Q    Median        3Q       Max  
## -0.14862  -0.02494  -0.00151   0.02530   0.17941  
## 
## Coefficients:
##                     Estimate Std. Error t value Pr(>|t|)    
## (Intercept)         0.258555   0.001900  136.06   <2e-16 ***
## distanceForagerTRUE 0.182532   0.002687   67.92   <2e-16 ***
## ---
## Signif. codes:  0 '***' 0.001 '**' 0.01 '*' 0.05 '.' 0.1 ' ' 1
## 
## (Dispersion parameter for gaussian family taken to be 0.001805488)
## 
##     Null deviance: 10.1314  on 999  degrees of freedom
## Residual deviance:  1.8019  on 998  degrees of freedom
## AIC: -3475
## 
## Number of Fisher Scoring iterations: 2
```

```
modelDT6 <- glm(DET6 ~ distanceForager, data = DTSimHighSight)
summary(modelDT6)
```

```
## 
## Call:
## glm(formula = DET6 ~ distanceForager, data = DTSimHighSight)
## 
## Deviance Residuals: 
##       Min         1Q     Median         3Q        Max  
## -0.007071  -0.007071  -0.001158  -0.000303   0.084287  
## 
## Coefficients:
##                      Estimate Std. Error t value Pr(>|t|)    
## (Intercept)         0.0011580  0.0003629   3.191  0.00146 ** 
## distanceForagerTRUE 0.0059129  0.0005132  11.522  < 2e-16 ***
## ---
## Signif. codes:  0 '***' 0.001 '**' 0.01 '*' 0.05 '.' 0.1 ' ' 1
## 
## (Dispersion parameter for gaussian family taken to be 6.583791e-05)
## 
##     Null deviance: 0.074447  on 999  degrees of freedom
## Residual deviance: 0.065706  on 998  degrees of freedom
## AIC: -6786.4
## 
## Number of Fisher Scoring iterations: 2
```

It is these simulations to which I compare the empirical data, which I recalculated DETs at minl = 3 to double check.

```
setwd("~/Manuscripts/Traplining Vervets")
PrimateDET_Data <- read.csv("DET data.csv") #this is the DET data tab saved as a csv
PrimateDET_Data$Seq <- strsplit(as.character(PrimateDET_Data$Seq), split = ",") #turn vector of factors into list of character vectors
PrimateDET_Data <- PrimateDET_Data[1:183, 1:8] #remove extraneous rows and colums(note stray value in row 73)
DET3_compare <- sapply(PrimateDET_Data$Seq, determinism, minl = 3)
summary(PrimateDET_Data$DET..3. - DET3_compare) # results mostly the same, check few differences
```

```
##      Min.   1st Qu.    Median      Mean   3rd Qu.      Max.      NA's 
## -0.009269  0.000000  0.000000  0.001025  0.000000  0.150000        12
```

```
PrimateDET_Data$DET6 <- sapply(PrimateDET_Data$Seq, determinism, minl = 6)
PrimateDETs_cleaned <- PrimateDET_Data[sapply(PrimateDET_Data$Bin.of.10, function(X) X %in% c(1:20)),] #removes incomplete bins. Including partial sets requires some thought - probably simulating sequences with each possible number of trials. Shouldn't be too hard
colnames(PrimateDETs_cleaned) <- c("Array", "Species", "Ind", "Ind.total.trials", "binNum", "DET3", "DET.all.trials", "Seq", "DET6") #clean up and standardize column names
```

There were a few small difference in my results from yours, which may be worth investigating, but overall things look the same. Next I set up a dataframe to compare simulated and empiracal DET values . . .

```
analysis_df <- DTSimHighSight[,-c(1,2)]
names(analysis_df) <- c("Species", "DET3", "DET6")
analysis_df$Array = "DT"
analysis_df$Species[analysis_df$Species] <- "distanceWeightedSim" #turns dForager logical into generating model name
analysis_df$Species[analysis_df$Species == "FALSE"] <- "randomSim" #turns dForager logical into name
analysis_df$Ind <- as.character(1:nrow(analysis_df)) #is this the right way to handle random effect of Individual in simulation data?
analysis_df$Ind.total.trials <- NA
analysis_df$binNum <- NA #may need to add if want to include sims in an interaction model with binNum*Species
analysis_df$DET.all.trials <- NA
analysis_df$Seq <- NA #accidently removed sequences variable! be sure to save for next simulation!
analysis_df <- rbind(analysis_df, PrimateDETs_cleaned)
analysis_df$Species = factor(analysis_df$Species, c("distanceWeightedSim", "randomSim", "Aye-aye", "Dwarf lemur", "Mouse lemur", "Vervet")) # sets order of factor levels for easier comparison to distanceWeightedSim in model output
```

. . . Visualized the resulting data . . .

```
ggplot(analysis_df, aes(x = Species, y = DET3)) + geom_boxplot() + theme_classic() #primates appear to be higher than simulations!
```

```
ggplot(analysis_df, aes(x = Species, y = DET6)) + geom_boxplot() + theme_classic() #Vertvets do show up pretty high here. Worth looking into the data to see if we can figure out why (do they start in the same location more? develop preferred decisions? Choose NN more often? I think you did this and decided on the last one)
```

. . . and built a linear model

```
#linearDETModel <- lmer(DET3 ~ Species + 1|Ind, data = analysis_df) #doesn't like random effects, this should be figured out
linearDETModel <- glm(DET3 ~ Species, data = analysis_df) #this should maybe just be an ANOVA?
summary(linearDETModel) #Significant effects for all but mouse lemurs
```

```
## 
## Call:
## glm(formula = DET3 ~ Species, data = analysis_df)
## 
## Deviance Residuals: 
##       Min         1Q     Median         3Q        Max  
## -0.297367  -0.028005  -0.000959   0.029065   0.260424  
## 
## Coefficients:
##                     Estimate Std. Error t value Pr(>|t|)    
## (Intercept)         0.441088   0.002776 158.909  < 2e-16 ***
## SpeciesrandomSim   -0.182532   0.003925 -46.500  < 2e-16 ***
## SpeciesAye-aye      0.115256   0.035942   3.207  0.00138 ** 
## SpeciesDwarf lemur  0.117604   0.017437   6.745 2.46e-11 ***
## SpeciesMouse lemur  0.034292   0.025490   1.345  0.17880    
## SpeciesVervet       0.289229   0.006857  42.182  < 2e-16 ***
## ---
## Signif. codes:  0 '***' 0.001 '**' 0.01 '*' 0.05 '.' 0.1 ' ' 1
## 
## (Dispersion parameter for gaussian family taken to be 0.003852316)
## 
##     Null deviance: 26.0702  on 1119  degrees of freedom
## Residual deviance:  4.2915  on 1114  degrees of freedom
## AIC: -3039.8
## 
## Number of Fisher Scoring iterations: 2
```

This is a very simple model, and I think it will be important to figure out how to add random effects of indivdual to this, which I was not able to figure out (it is tricky to consider what an “individual” is in the simulated data). As is, it is essentially just an ANOVA, which comes with the advantage that it is easy to explain. When looking at DET with minL of six, the floor effect I mentioned earlier is apparent, with many 0s. At DET with minl = 3, however, the data have a nicer, more normal distribution, with significant differences in ALL species from the random choice model and all but the mouse lemurs in the distance discounting model.

So, here are my major takeaways:

1. Simulating movement with high sight is the correct choice because it reflects the reality of our experiments. However, doing so obviates the need for a spatially explicit simulation. We could just as well have built transition matrices to generate our null sequences.
2. Because animals can see all of the platforms/patches, I am of the opinion that this is NOT traplining. Traplining, as it seems to be used throughout the literature, implies spatial memory: the formation of routes from learned responses to visual cues. Our primates are not doing this.
3. Our data DO show that primates choices (except mouse lemurs) are more consistent than expected even given strong distance discounting. This suggests either a very strong nearest neighbor heuristic, or some other consistent decision making bias/preference. You have already gone a bit into what that might be, and this analysis could be a valuable way of approaching that.
4. The ability for foragers to avoid an increasing number of recently visited patches also has an effect on DET, and the effect changes with minL. This IS an aspect of memory at play in the platform experiments, a property of DET not discussed by Ayers, and potentially a really interesting angle to take.
