# Supplementary material for: Mild movement sequence repetition in five primate species and evidence for a taxonomic divide in cognitive mechanisms
Source: Sci Rep. 2022 Aug 25;12:14503. doi: 10.1038/s41598-022-18633-7 (PMC9411198; doi:10.1038/s41598-022-18633-7)
Supplement: Supplementary file 3 — Supplementary Information 3. [file 41598_2022_18633_MOESM3_ESM.pdf]

Array Navigation Learning Model

10/14/2021

In the following code, we investigate species level differences in learning rates of four species of primates. The task the primates are learning is to visit all locations within a local array shaped like a double trapezoid, a pentagon, and a Z. We model fit a model of the total distance an individual animal travels before visiting all locations relative to the number of trials that individual has undergone, the species of the individual, and the individuals own initial performance and learning rate. Because we expect learning curves to follow a power-law function, we log-log transform the distance traveled and trial number. Prior to running this analysis, the script "Traplining_Data_Processing_Script.R" was run to prepare the data. Not all of the descriptive statistics calculated in this script were ultimately used. This script is also where simulations were run.

First, we load the data and conduct log transformations

```
knitr::opts_chunk$set(echo = TRUE)
setwd("C:/Users/avining/Documents/Manuscripts/Do Primates Trapline")

#enable easy changes in text size, for tidy LaTeX in pdf
def.chunk.hook  <- knitr::knit_hooks$get("chunk")
knitr::knit_hooks$set(chunk = function(x, options) {
  x <- def.chunk.hook(x, options)
  ifelse(options$size != "normalsize",
         paste0("\n \\", options$size,"\n\n", x, "\n\n \\normalsize"),
         x)
})

library(tidyverse)
library(rstan)
library(bayesplot)
library(RColorBrewer)

data_cleaned <- read_csv("./Results/data_cleaned.csv",col_types = "dccccdddddc")

#log/log transform data to model a power function using linear fit
data_cleaned$Distance_scaled <- log(data_cleaned$Distance)
data_cleaned$Trial_scaled <- log(data_cleaned$Trial)
data_cleaned$Source <- gsub("Learning", "Reinforcement", data_cleaned$Source)
```

Then, we analyze the results from each array separately, starting with the Double Trapezoid. Trials were conducted in the this way with aye ayes, dwarf lemurs, mouse lemurs, and vervets.

Double Trapezoid (DT) Analysis

We begin by visualizing the data and perparing for analysis in STAN

```
#remove simulation data, data from arrays other than Double Trapezoid, and any individuals that complet
DT_data <- data_cleaned %>% filter(Source == "Experimental", Array == "DT", max(Trial) >= 5)
DT_data$Species <- factor(DT_data$Species)
DT_data$ID <- factor(DT_data$ID)

#plot raw data
ggplot(DT_data) + geom_point(aes(x = Trial_scaled, y = Distance_scaled), alpha = 0.4) +
  facet_wrap(~Species)
```

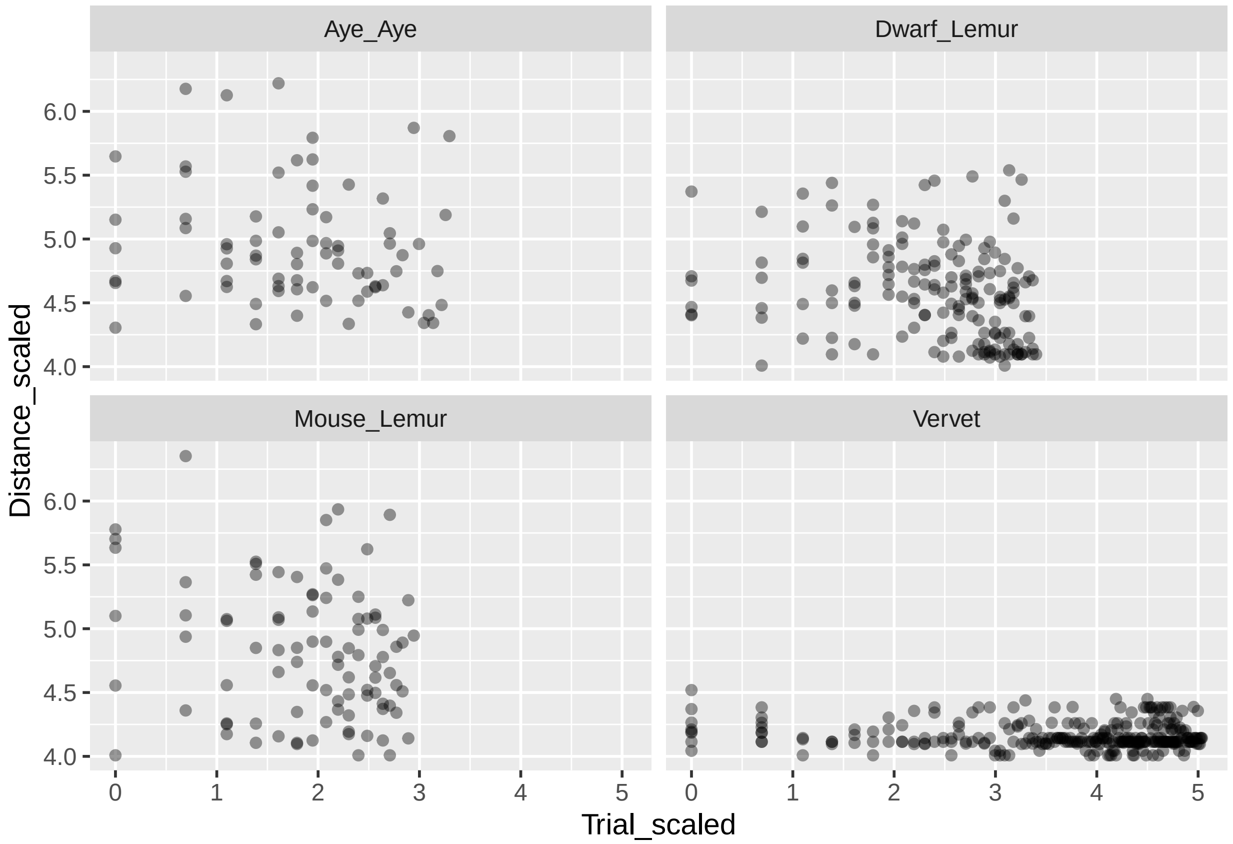


```
#Restructure data for STAN analysis.
stan_data <- list(N = nrow(DT_data),
                  J = length(unique(DT_data$ID)),
                  K = length(unique(DT_data$Species)),
                  y = DT_data$Distance_scaled,
                  x = DT_data$Trial_scaled,
                  id = as.numeric(DT_data$ID),
                  sp = as.numeric(DT_data$Species))
```

In the above plot we see the log-log scaled data through which we will attempt a linear fit (analogous to a power function fit through the raw data). They do appear to show decreasing trends in all species but the vervet, which exhibits shorter distances traveled on initial trials. To investigate these trends, we fit the data to our model described in the manuscript "Do primates really trapline? Mild recursion and evidence for variable cognitive mechanisms in five species".

```r
options(mc.cores = parallel::detectCores())
fit0 <- stan(file = "../CODE/DT_power_model.stan",
             data = stan_data,
             chains = 4,
             iter = 14000,
             warmup = 7000,
             control = list(max_treedepth = 15))

save(fit0, file = "../Results/power_model_fit0.Rdata")
```

Model Diagnostics

Before interpreting the posterior distributions of the parameters in this model, we assess the MCMC chains for convergence, the posterior distributions for normality, and the residuals of the model for normality and constant variance.

```r
load("../Results/power_model_fit0.Rdata")
print(fit0, pars=c("mu_0", "mu_species", "b_0", "b_species", "sigma_e", "lp__"), probs = c(.1, .5,.9))
```

```
## Inference for Stan model: DT_power_model.
## 4 chains, each with iter=14000; warmup=7000; thin=1;
## post-warmup draws per chain=7000, total post-warmup draws=28000.
##
##                  mean se_mean    sd    10%    50%    90% n_eff Rhat
## mu_0             4.83    0.00  0.45   4.24   4.83   5.40  9915 1.00
## mu_species[1]    0.37    0.00  0.47  -0.23   0.36   0.97 10555 1.00
## mu_species[2]    0.13    0.00  0.46  -0.46   0.13   0.72 10441 1.00
## mu_species[3]    0.19    0.00  0.47  -0.41   0.19   0.79 10194 1.00
## mu_species[4]   -0.66    0.00  0.45  -1.23  -0.66  -0.07  9744 1.00
## b_0             -0.08    0.00  0.44  -0.65  -0.08   0.49  9411 1.00
## b_species[1]    -0.07    0.00  0.45  -0.64  -0.07   0.51  9579 1.00
## b_species[2]    -0.08    0.00  0.45  -0.65  -0.07   0.49  9411 1.00
## b_species[3]    -0.03    0.00  0.45  -0.61  -0.03   0.54  9566 1.00
## b_species[4]     0.09    0.00  0.44  -0.48   0.10   0.66  9398 1.00
## sigma_e[1]       0.76    0.00  0.07   0.68   0.75   0.84 40460 1.00
## sigma_e[2]       0.60    0.00  0.03   0.55   0.59   0.64 45397 1.00
## sigma_e[3]       0.79    0.00  0.06   0.71   0.79   0.87 40206 1.00
## sigma_e[4]       0.21    0.00  0.01   0.19   0.20   0.22 47928 1.00
## lp__           792.07    2.25 28.08 764.88 785.08 829.02   156 1.03
##
## Samples were drawn using NUTS(diag_e) at Wed Aug 18 17:44:35 2021.
## For each parameter, n_eff is a crude measure of effective sample size,
## and Rhat is the potential scale reduction factor on split chains (at
## convergence, Rhat=1).
```

```r
traceplot(fit0, pars = c("mu_0", "mu_species", "b_0", "b_species", "lp__"), inc_warmup = TRUE) #check c

#look for estimation anomalies: all posteriors are normally distributed, with appropriate levels of cor
pairs(fit0, pars = c("mu_0", "mu_species", "sigma_e"), las = 1)
pairs(fit0, pars = c("b_0", "b_species", "sigma_e"), las = 1)
pairs(fit0, pars = c("mu_0", "mu_species", "b_0", "b_species", "lp__"), las = 1)
```

```r
#residual analysis
r_bar <- apply(extract(fit0, "pearson_residual")[[1]], MARGIN = 2, mean)
y_pred <- apply(extract(fit0, "y_pred")[[1]], MARGIN = 2, mean)
plot(x = y_pred, y = r_bar) #residuals against prediction
plot(x = stan_data$x, y = r_bar)
plot(x = stan_data$sp, y = r_bar)
qqnorm(r_bar)
```

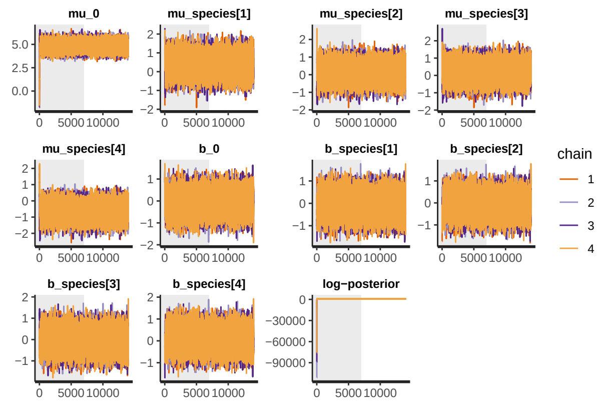

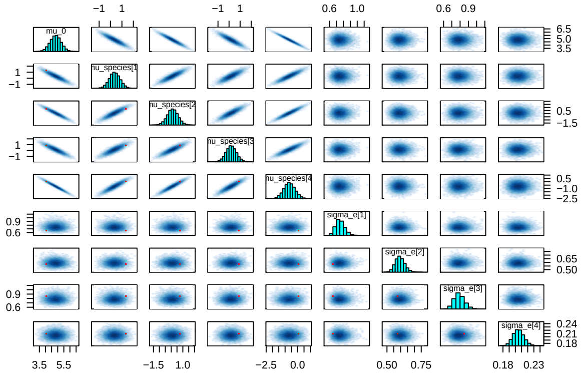

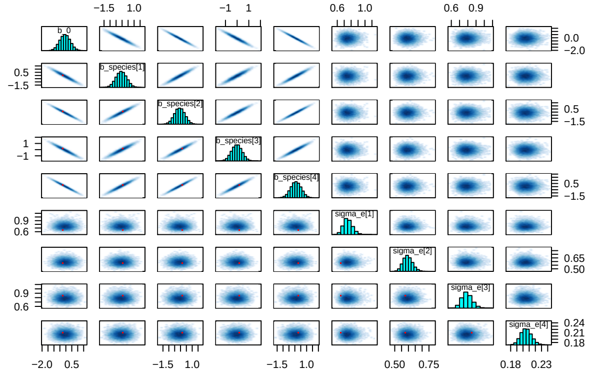

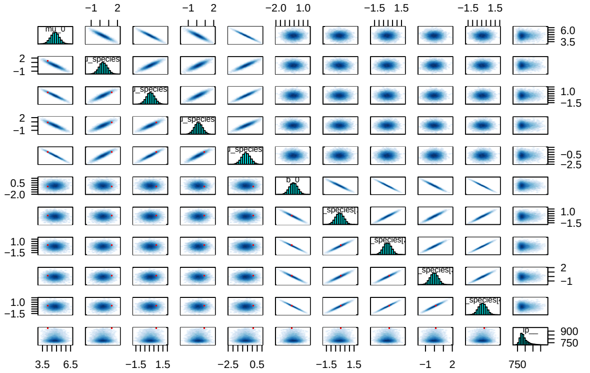

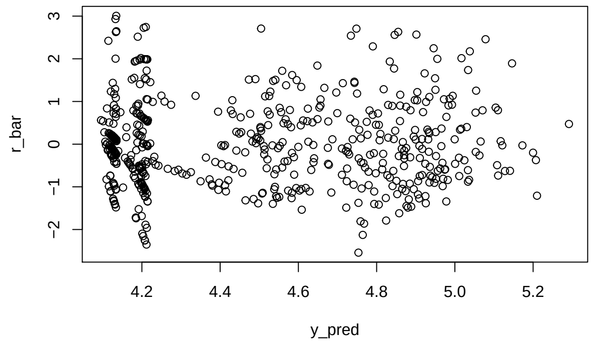

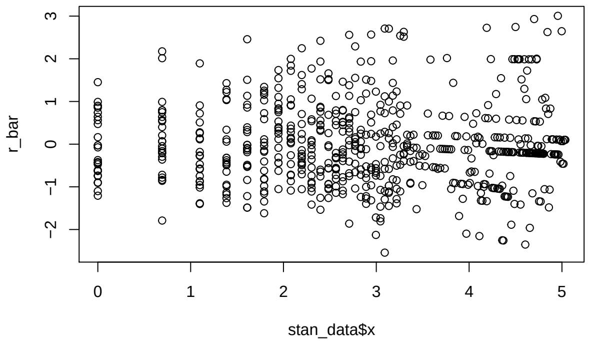

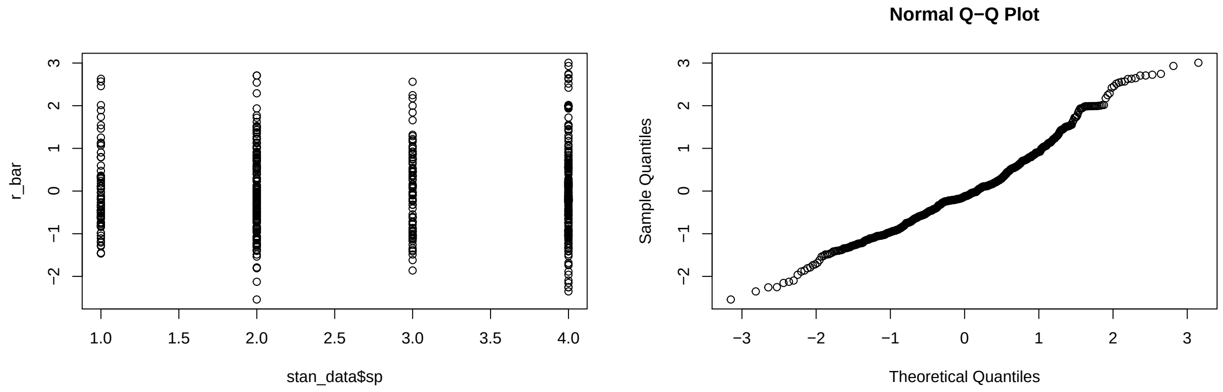

From the R-hats and effective sample sizes (n_effs) in the model summary (and from the visualization of parameter estimates across each MCMC chain) we can see that the posterior distribution of models converged across the four chains, and that the parameter space was well mixed in all chains. In pair plots of the model parameters, we can see that posterior distributions of all parameters appear normal, and there are no patterns to suggest the model was not identifiable. We do see high correlation among parameter estimates of the intercept and among parameter estimates of the slope. This is to be expected as we estimate both a grand mean for both parameter, and species level adjustments (normaly distributed around zero); this means that as the estimated grand mean moves around in the posterior, species level adjustments will change accordingly to bring the species level intercept estimate (grand mean plus species adjustment) close to the the true value.

Plots of the residuals attest to the good fit between our statistical model and the structure of the data. Across species, trial nunmber, and outcomes (distance travelled), residuals show no visible trend and remain evenly distributed.

Model Interpretation

Before assessing our posterior distributions, we need to add the estimated species and individual deviations from each sample of the posterior to the grand means from the same sample to find the actual posterior estimates of the intercept and slope for each species/individual.

```
posteriors_DT <- extract(fit0)

#find the estimated intercepts by species for each sample. Used for plotting estimated learning curves.
posteriors_DT$mu_species <- apply(posteriors_DT[["mu_species"]],
                                  MARGIN = 2,
                                  FUN = function(X) X + posteriors_DT[["mu_0"]])

posteriors_DT$b_species <- apply(posteriors_DT[["b_species"]],
                                 MARGIN = 2,
                                 FUN = function(X) X + posteriors_DT[["b_0"]])

for(i in 1:ncol(posteriors_DT[["mu_id"]])){
  #get species of id factor level i
  sp <- DT_data$Species[match(levels(DT_data$ID), DT_data$ID)][i]
  #add individual deviation to species level intercept
  posteriors_DT[["mu_id"]][,i] <- posteriors_DT[["mu_species"]][,sp] + posteriors_DT[["mu_id"]][,i]
  #add individual deviation to species level slope
  posteriors_DT[["b_id"]][,i] <- posteriors_DT[["b_species"]][,sp] + posteriors_DT[["b_id"]][,i]
}
```

To visualize the resulting posteriors, we determine quantiles of each posterior distribution and plot the 80% (thick bars) and 90% (thin bars) confidence intervals for each species and individual level parameter.

```r
#function to get quantiles from each column of a matrix (corresponding to the posterior distribution of a variable)
apply_quant <- function(x) apply(as.matrix(x), MARGIN = 2, quantile, c(0.025,.05, .10, .5, .90, .95, 0.975))

#get quantiles for the first 8 parameters in "posteriors" list
posteriors_DT[1:8] %>% lapply(FUN = apply_quant) %>%
  #bind list of quanitile vectors into a matrix
  do.call(args = ., what = cbind) %>%
  #transpose and save as dataframe
  t() %>% as.data.frame() -> posterior_quantiles_DT

#remove % and start all names with a character to avoid trouble in ggplot
colnames(posterior_quantiles_DT) <- paste("X", gsub("%", "", colnames(posterior_quantiles_DT)), sep = "")

#add metadata to describe each parameter with quantiles
posterior_quantiles_DT$parameter <- names(unlist(sapply(posteriors_DT[1:8],
                                                   function(X) seq(to = ncol(as.matrix(X))))))
#gets a vector named with parameter names for each type of parameter, then extracts and concatenates those names.

posterior_quantiles_DT$param_type <- rep(names(posteriors_DT[1:8]),
                                    times = sapply(posteriors_DT[1:8],
                                                   function(X) ncol(as.matrix(X))))

#~~give species metadata for parameters where relevant
posterior_quantiles_DT$species <- NA
posterior_quantiles_DT$species[posterior_quantiles_DT$param_type == "mu_id"] <-
  as.character(DT_data$Species[match(levels(DT_data$ID), DT_data$ID)])
posterior_quantiles_DT$species[posterior_quantiles_DT$param_type == "b_id"] <-
  as.character(DT_data$Species[match(levels(DT_data$ID), DT_data$ID)])
posterior_quantiles_DT$species[posterior_quantiles_DT$param_type == "mu_species"] <-
  levels(DT_data$Species)
posterior_quantiles_DT$species[posterior_quantiles_DT$param_type == "b_species"] <-
  levels(DT_data$Species)
#~~~~~~~~~~
```

```r
#plot intercept(mu) parameters
mu_posterior_plot_DT <- posterior_quantiles_DT %>%
  filter(param_type %in% c("mu_species", "mu_id")) %>%
  arrange(species) %>%
  mutate(parameter = factor(parameter, levels = parameter[length(parameter):1])) %>%
  ggplot(aes(y = parameter)) +
  geom_pointrange(aes(x = X50,
                      xmin = X10,
                      xmax = X90,
                      color = species,
                      alpha = param_type),
                  orientation = "y", size = 1.5, fatten = 1.5) +
  geom_linerange(aes(xmin = X5,
                     xmax = X95,
                     color = species,
                     alpha = param_type),
                 orientation = "y", size = 0.8) +
  scale_alpha_discrete(range = c(.4, 1), name = "Parameter Level", labels = c("Individual", "Species")) +
  scale_color_brewer(palette = "Dark2") +
  theme_bw() +
  theme(legend.key.size = unit(0.75, "cm")) +
  labs(x = "log(Minimum Distance Travelled)",
       y = "Parameter Name",
       title = "Estimated Distance Travelled on First Trial for Species and Individuals")

print(mu_posterior_plot_DT)
```

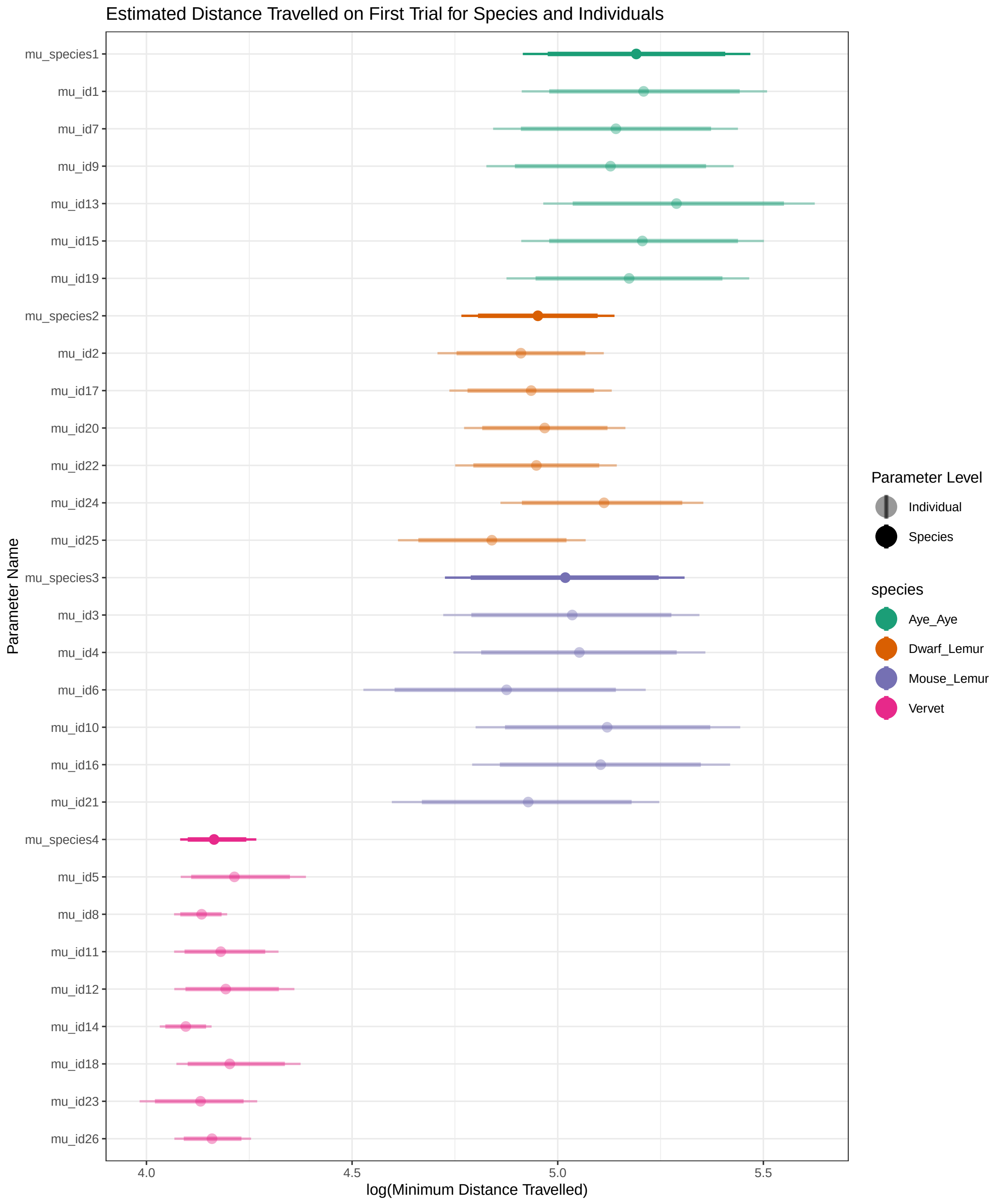


```
#plot slope (b) paramters
b_posterior_plot_DT <- posterior_quantiles_DT %>%
  filter(param_type %in% c("b_species", "b_id")) %>%
  arrange(species) %>%
  mutate(parameter = factor(parameter, levels = parameter[length(parameter):1])) %>%
  ggplot(aes(y = parameter)) +
  geom_pointrange(aes(x = X50,
                      xmin = X10,
```

```r
                      xmax = X90,
                      alpha = param_type,
                      color = species),
                  orientation = "y", size = 1.5, fatten = 1.5) +
  geom_linerange(aes(xmin = X5,
                     xmax = X95,
                     alpha = param_type,
                     color = species),
                 orientation = "y", size = 0.8) +
  scale_alpha_discrete(range = c(.4, 1), name = "Parameter Level", labels = c("Individual", "Species")) +
  scale_color_brewer(palette = "Dark2") +
  geom_vline(xintercept = 0, color = "red", linetype = "dashed") +
  theme_bw() +
  theme(legend.key.size = unit(0.75, "cm")) +
  labs(x = "log(Distance)/log(Trial)",
       y = "Slope Parameter",
       title = "Estimated Learning Rates for Species and Individuals")

print(b_posterior_plot_DT)
```

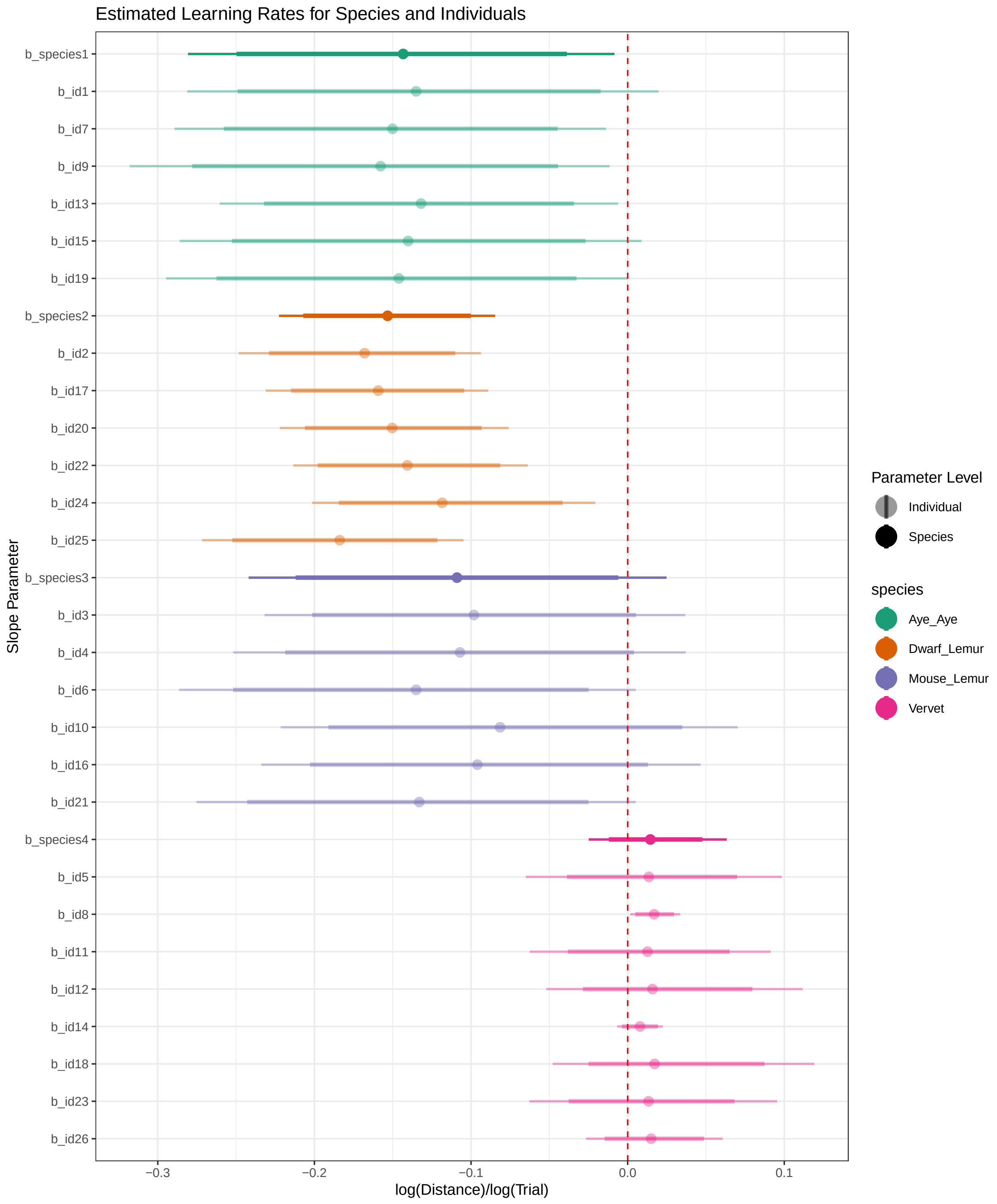
Estimated Learning Rates for Species and Individuals
b_species1
b_id1
b_id7
b_id9
b_id13
b_id15
b_id19
b_species2
b_id2
b_id17
b_id20
b_id22
b_id24
b_id25
b_species3
b_id3
b_id4
b_id6
b_id10
b_id16
b_id21
b_species4
b_id5
b_id8
b_id11
b_id12
b_id14
b_id18
b_id23
b_id26
Slope Parameter
−0.3
−0.2
−0.1
0.0
0.1
log(Distance)/log(Trial)
Parameter Level
Individual
Species
species
Aye_Aye
Dwarf_Lemur
Mouse_Lemur
Vervet

```
## pdf
##   2

## pdf
##   2
```

To visualize how the full set of parameter estimates come to together in our regression model, we plot a line using the mean intercept and slope for each species through the raw data. Additionally, we add the regression lines from 100 randomly selected samples using a more transparent color to illustrate the uncertainty of our estimates.

```
Trial <- 1:max(DT_data$Trial)
pal <- brewer.pal(n = 8, name = "Dark2")
species <- c("Aye_Aye", "Dwarf_Lemur", "Mouse_Lemur", "Vervet")
sample_set <- sample(1:4000, size = 100)

#Figure for Manuscript
tiff("../Docs/Supp_Figure 4_Posterior_Predictions.tif", width = 1600, height = 1200)
par(mfrow = c(2,2),
    mar = c(6,8,6,6))
for (i in 1:4) { #loop over each species (column in posterior estimates)
  plot(1, type = "n", xlim = c(0, max(DT_data$Trial_scaled)), ylim = c(4,6.5), ylab = NA, xlab = NA, ma
  title(ylab = "log(Distance)", xlab = "log(Trial)", cex.lab = 3, line = 4)
  Distance_mean <- mean(posteriors_DT$mu_species[,i]) + mean(posteriors_DT$b_species[,i]) * log(Trial)
  lines(x = log(Trial), y = Distance_mean, col = pal[i], lwd = 3)
  for (j in sample_set) {
    lines(x = log(Trial), y = posteriors_DT$mu_species[j,i] + posteriors_DT$b_species[j,i] * log(Trial)
  }
  species_data <- filter(DT_data, Species == species[i])
  points(x = species_data$Trial_scaled, y = species_data$Distance_scaled, col = pal[i], pch = 19, cex =
}
dev.off()
```

```
## pdf
##   2
```

```
#Figure for Supplement
for (i in 1:4) { #loop over each species (column in posterior estimates)
  plot(1, type = "n", xlim = c(0, max(DT_data$Trial_scaled)), ylim = c(3.5,7), ylab = NA, xlab = NA, ma
  title(ylab = "log(Distance)", xlab = "log(Trial)", cex.lab = 1, line = 2)
  Distance_mean <- mean(posteriors_DT$mu_species[,i]) + mean(posteriors_DT$b_species[,i]) * log(Trial)
  lines(x = log(Trial), y = Distance_mean, col = "red", lwd = 3)
  for (j in sample_set) {
    lines(x = log(Trial), y = posteriors_DT$mu_species[j,i] + posteriors_DT$b_species[j,i] * log(Trial)
  }
  points(x = filter(DT_data, Species == levels(DT_data$Species)[i])$Trial_scaled,
         y = filter(DT_data, Species == levels(DT_data$Species)[i])$Distance_scaled,
         pch = 19, cex = 0.5, col = pal[i])
}
```

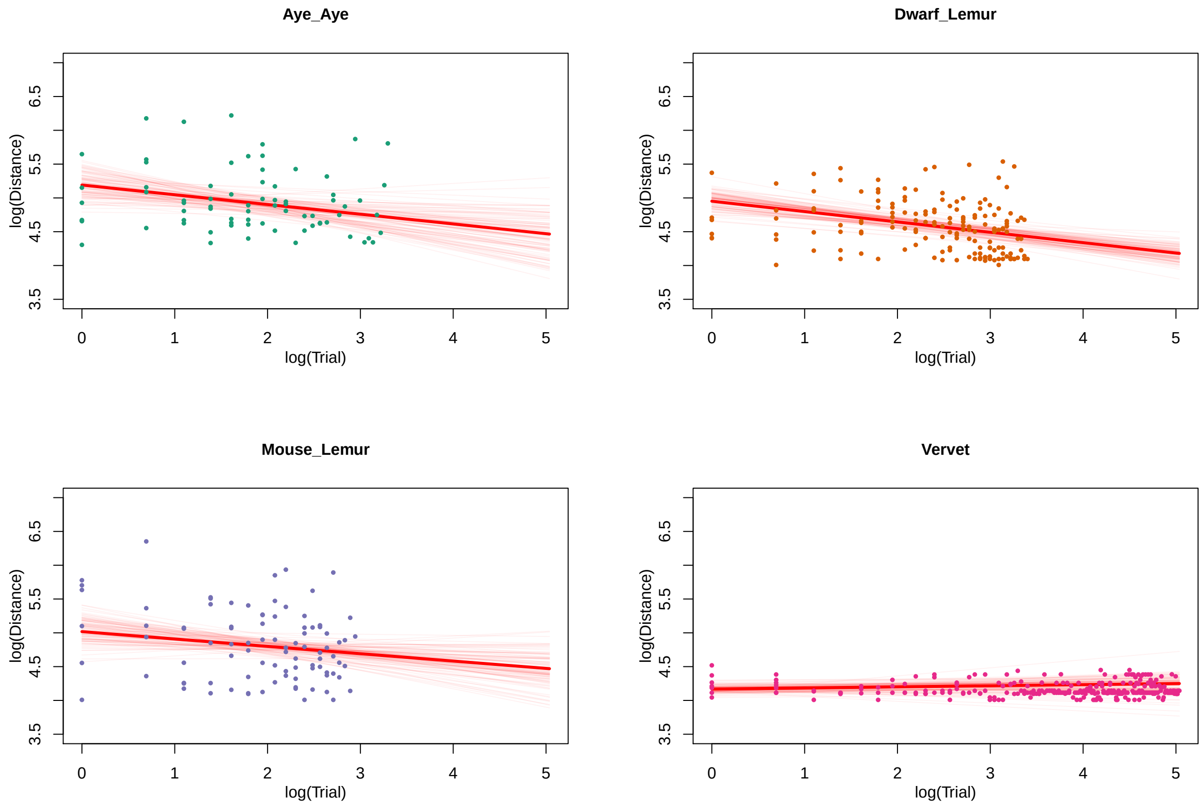

Finally, we report some specific numbers for use in final publication

```
#percent of posterior sample below 0 for slope of vervets
sum(posteriors_DT$b_species[,4] <=0)/length(posteriors_DT$b_species[,4])
```

```
## [1] 0.2043214
```

Pentagon Array Analysis

We analyze data from the Pentagon Array, which was only completed by vervets, in the same manner as the Double Trapezoid

```
#remove simulation data, data from arrays other than Pentagon, and any individuals that completed fewer
Pentagon_data <- data_cleaned %>%
  filter(Source == "Experimental", Array == "Pentagon", max(Trial) >= 5)

Pentagon_data$Species <- factor(Pentagon_data$Species)
Pentagon_data$ID <- factor(Pentagon_data$ID)

#Visualize data and restructure for analysis in STAN
ggplot(Pentagon_data) + geom_jitter(aes(x = Trial_scaled, y = Distance_scaled), alpha = 0.6) +
  facet_wrap(~Species)
```

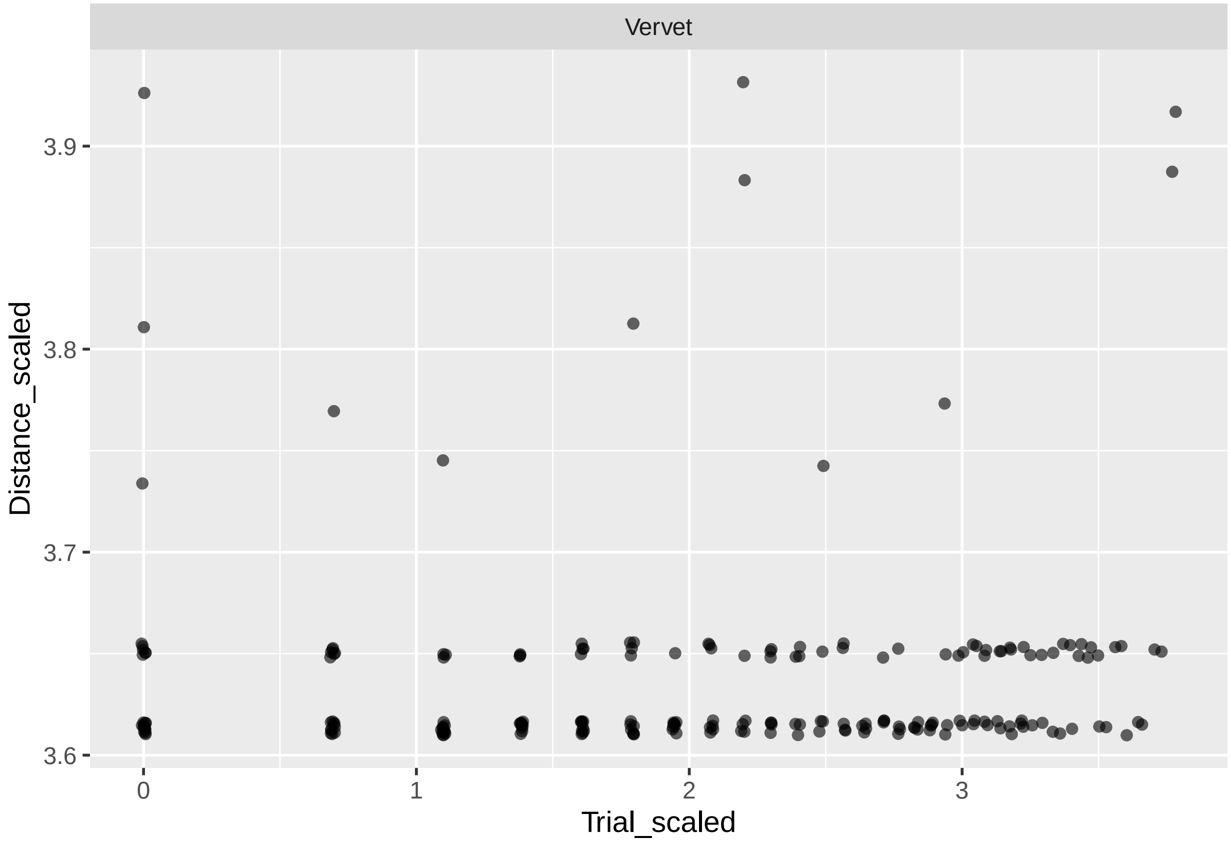


```r
stan_data_Pentagon <- list(N = nrow(Pentagon_data),
                           J = length(unique(Pentagon_data$ID)),
                           K = length(unique(Pentagon_data$Species)),
                           y = Pentagon_data$Distance_scaled,
                           x = Pentagon_data$Trial_scaled,
                           id = as.numeric(Pentagon_data$ID),
                           sp = as.numeric(Pentagon_data$Species))
```

It can be seen from the plot of the raw data that vervets initially chose routes through the Pentagon array that were close to the shortest possible route, and that performance did not change much over time and experience. To analyze these patters, we fit the data to the same statistical model as in the Double Trapezoid.

```r
options(mc.cores = parallel::detectCores())
fit_Pentagon <- stan(file = "../CODE/DT_power_model.stan",
             data = stan_data_Pentagon,
             chains = 4,
             iter = 8000,
             warmup = 4000,
             control = list(max_treedepth = 15))

save(fit_Pentagon, file = "../Results/power_model_Pentagon.Rdata")
```

Model Diagnostics

After fitting the model in STAN, we asses the resulting MCMC chains and posterior distributions for convergence and mixing, then examine to residuals to assess how well the model fits the structure of the data

```r
load("../Results/power_model_Pentagon.Rdata")
print(fit_Pentagon, pars=c("mu_0", "mu_species", "b_0", "b_species", "sigma_e", "lp__"), probs = c(.1,
```

```
## Inference for Stan model: DT_power_model.
## 4 chains, each with iter=8000; warmup=4000; thin=1;
## post-warmup draws per chain=4000, total post-warmup draws=16000.
##
##                  mean se_mean    sd    10%    50%    90% n_eff Rhat
## mu_0             4.21    0.01  0.69   3.32   4.24   5.10  8082 1.00
## mu_species[1]   -0.59    0.01  0.69  -1.47  -0.61   0.30  8094 1.00
## b_0              0.01    0.01  0.70  -0.88   0.03   0.89  7008 1.00
## b_species[1]     0.00    0.01  0.70  -0.88  -0.02   0.89  7006 1.00
## sigma_e[1]       0.09    0.00  0.00   0.08   0.09   0.10  1223 1.00
## lp__           699.84    4.85 35.16 666.20 692.34 739.93    53 1.08
##
## Samples were drawn using NUTS(diag_e) at Tue Aug 24 14:29:13 2021.
## For each parameter, n_eff is a crude measure of effective sample size,
## and Rhat is the potential scale reduction factor on split chains (at
## convergence, Rhat=1).
```

```r
#check convergence, appears strong
traceplot(fit_Pentagon, pars = c("mu_0", "mu_species", "b_0", "b_species", "lp__"), inc_warmup = TRUE)

#look for estimation anomalies:
pairs(fit_Pentagon, pars = c("mu_0", "mu_species", "sigma_e"), las = 1)
pairs(fit_Pentagon, pars = c("b_0", "b_species", "sigma_e"), las = 1)
pairs(fit_Pentagon, pars = c("mu_0", "mu_species", "b_0", "b_species", "lp__"), las = 1)
# all posteriors are normally distributed, with appropriate levels of correlation in expected places

#residual analysis
r_bar <- apply(extract(fit_Pentagon, "pearson_residual")[[1]], MARGIN = 2, mean)
y_pred <- apply(extract(fit_Pentagon, "y_pred")[[1]], MARGIN = 2, mean)
plot(x = y_pred, y = r_bar) #residuals against prediction
plot(x = stan_data_Pentagon$x, y = r_bar)
plot(x = stan_data_Pentagon$sp, y = r_bar)
qqnorm(r_bar)
```

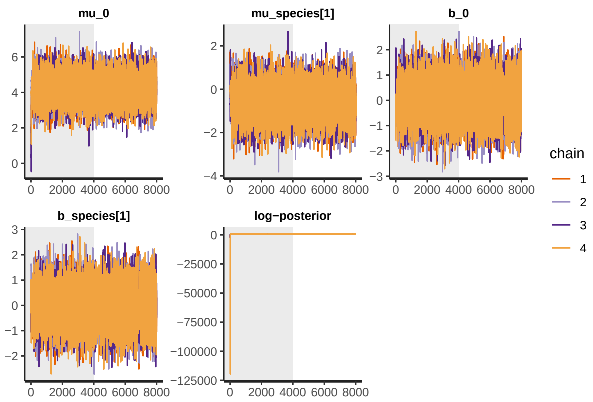
mu_0
mu_species[1]
b_0
b_species[1]
log−posterior
chain
1
2
3
4

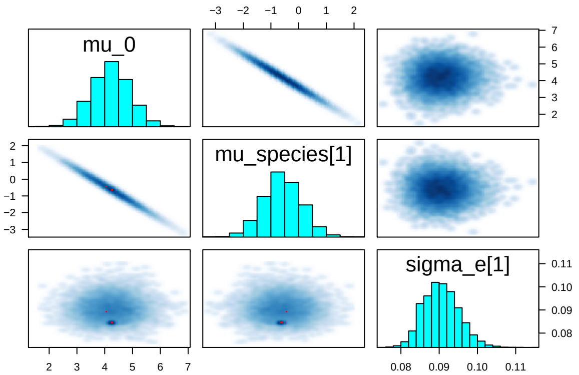
mu_0
mu_species[1]
sigma_e[1]

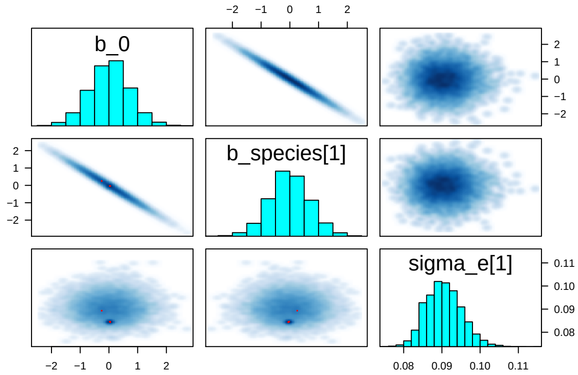
b_0
b_species[1]
sigma_e[1]

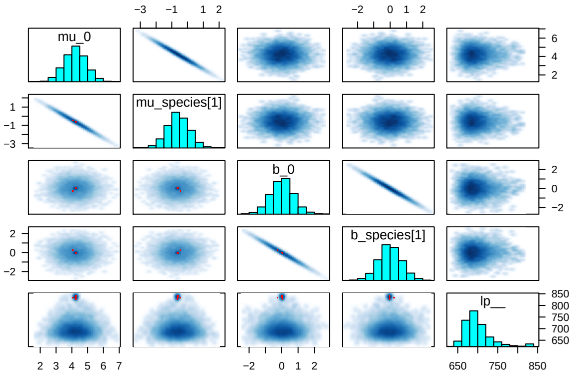
mu_0
mu_species[1]
b_0
b_species[1]
lp__

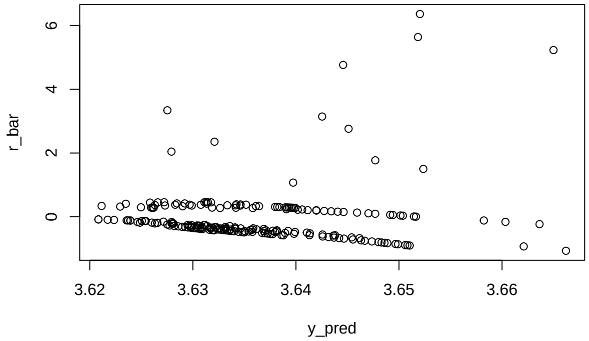
r_bar
y_pred

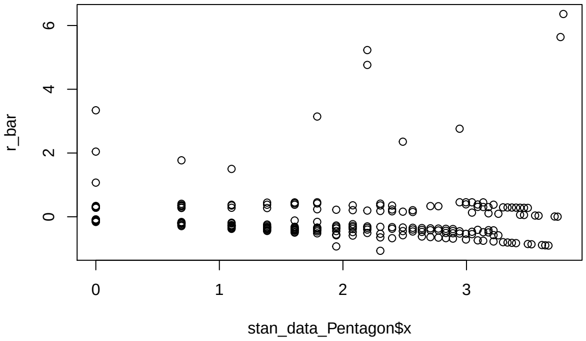
r_bar
stan_data_Pentagon$x

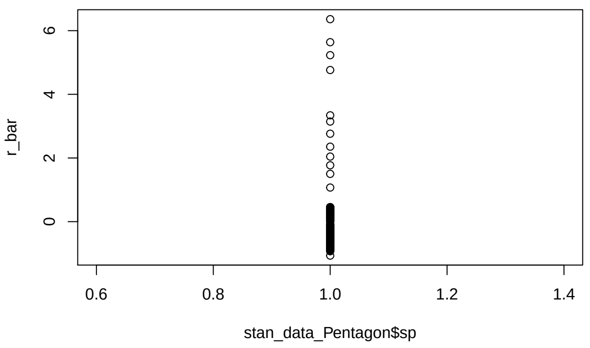

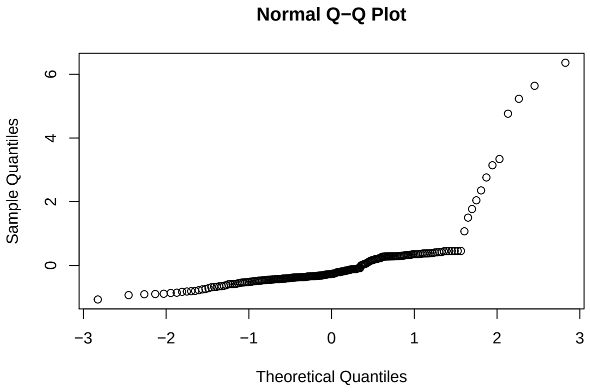

The MCM chains converge and show good mixing. The pairs plots indicate that parameter distributions in the posterior are normally distributed, with some expected correlation structure as described in the double trapezoid section. Red pixels in the pairs plot indicate samples with divergent transitions. Their placement in the sigma_e distributions suggest that these have to do with challenges estimating the correct error variance, while their placement in the distribution of b_0 suggests that this only occurs when the slope is estimated at 0. Their placement in the distribution of log-probability of the samples (high) suggests that when the model slope is estimated at 0, the error variance can be reduced, producing a better fit model, but that this requires a large change in the estimated error variance that creates warnings. Taken together, these divergent trasitions highlight that running a model with species level deviations around a grand mean for data with only 1 species and structure, discrete outcomes in a continuous variable isn't the best approach. We might consider simplifying the model for this array.

Accordingly, when looking at the residuals, it is revealed that this statistical model does not fit the structure of the data quite as well as in the Double Trapezoid. Based on the patterns in the residual plots and what we know about the structure of this experiment, this is likely because the outcome (distance traveled) has little room for gradual improvement: instead vervets are switching between a limited set of options with two, discrete possibilities for outcome, and then occasionally switching to other, less effective routes. Likely, these data would be better modeled by some sort of discrete choice following a Poisson distribution. However, to enable comparisons between arrays, we stick with this model for analysis, and make note of the inconsistencies as a caveat in interpreting our results.

Model Interpretation

Before assessing the posterior distributions, we need to add the deviations for species and individuals to the grand means for each sample to find the actual posterior estimates of the intercept and slope for each species/individual.

```
posteriors_Pentagon <- extract(fit_Pentagon)

#find the estimated intercepts by species for each sample: Used for plotting estimated learning curves
posteriors_Pentagon$mu_species <- apply(posteriors_Pentagon[["mu_species"]],
                               MARGIN = 2,
                               FUN = function(X) X + posteriors_Pentagon[["mu_0"]])
posteriors_Pentagon$b_species <- apply(posteriors_Pentagon[["b_species"]], MARGIN = 2, FUN = function(X) X + posteriors_Pentagon

for(i in 1:ncol(posteriors_Pentagon[["mu_id"]])){
  #get species of id factor level i
  sp <- Pentagon_data$Species[match(levels(Pentagon_data$ID), Pentagon_data$ID)][i]
  #add individual deviation to species level intercept
  posteriors_Pentagon[["mu_id"]][,i] <- posteriors_Pentagon[["mu_species"]][,sp] + posteriors_Pentagon[["mu_id"]][,i]
  #add individual deviation to species level slope
  posteriors_Pentagon[["b_id"]][,i] <- posteriors_Pentagon[["b_species"]][,sp] + posteriors_Pentagon[["b_id"]][,i]
}
```

To visualize the resulting posteriors, we determine quantiles of each posterior distribution and plot the 80% (thick bars) and 90% (thin bars) confidence intervals for each species and individual level parameter.

```
#function to get quantiles from each column of a matrix (corresponding to the posterior distribution of a variable)

#get quantiles for the first 8 parameters in "posteriors" list
posteriors_Pentagon[1:8] %>% lapply(FUN = apply_quant) %>%
   #bind list of quanitile vectors into a matrix
  do.call(args = ., what = cbind) %>%
  #transpose and save as dataframe
  t() %>% as.data.frame() -> posterior_quantiles_Pentagon

#remove % and start all names with a character to avoid trouble in ggplot
colnames(posterior_quantiles_Pentagon) <- paste("X", gsub("%", "", colnames(posterior_quantiles_Pentagon)), sep = "")

#add metadata to describe each parameter with quantiles
#get a vector named with parameter names for each type of parameter, then extract and concatenates those names.
posterior_quantiles_Pentagon$parameter <- names(unlist(sapply(posteriors_Pentagon[1:8],
                                                     function(X) seq(to = ncol(as.matrix(X))))))

posterior_quantiles_Pentagon$param_type <- rep(names(posteriors_Pentagon[1:8]), times = sapply(posteriors_Pentagon[1:8],
                                                                                   function(X) ncol(as.matrix(X))))

#~~give species metadata for parameters where relevant
posterior_quantiles_Pentagon$species <- NA
posterior_quantiles_Pentagon$species[posterior_quantiles_Pentagon$param_type == "mu_id"] <-
  as.character(Pentagon_data$Species[match(levels(Pentagon_data$ID), Pentagon_data$ID)])
posterior_quantiles_Pentagon$species[posterior_quantiles_Pentagon$param_type == "b_id"] <-
  as.character(Pentagon_data$Species[match(levels(Pentagon_data$ID), Pentagon_data$ID)])
posterior_quantiles_Pentagon$species[posterior_quantiles_Pentagon$param_type == "mu_species"] <-
  levels(Pentagon_data$Species)
posterior_quantiles_Pentagon$species[posterior_quantiles_Pentagon$param_type == "b_species"] <-
  levels(Pentagon_data$Species)
#~~~~~~~~~~
```

```
#plot intercept(mu) parameters
mu_posterior_plot <- posterior_quantiles_Pentagon %>%
  filter(param_type %in% c("mu_species", "mu_id")) %>%
  arrange(species) %>%
  mutate(parameter = factor(parameter, levels = parameter[length(parameter):1])) %>%
  ggplot(aes(y = parameter)) +
  geom_pointrange(aes(x = X50,
                      xmin = X10,
                      xmax = X90,
                      color = species,
                      alpha = param_type),
                  orientation = "y", size = 1.5, fatten = 1.5) +
  geom_linerange(aes(xmin = X5,
                     xmax = X95,
                     color = species,
                     alpha = param_type),
                 orientation = "y", size = 0.8) +
  scale_alpha_discrete(range = c(.4, 1), name = "Parameter Level", labels = c("Individual", "Species")) +
  scale_color_brewer(palette = "Dark2") +
  theme_bw() +
  theme(legend.key.size = unit(0.75, "cm")) +
  labs(x = "log(Minimum Distance Travelled)",
       y = "Parameter Name",
       title = "Estimated Distance Travelled on First Trial for Species and Individuals")

print(mu_posterior_plot)
```

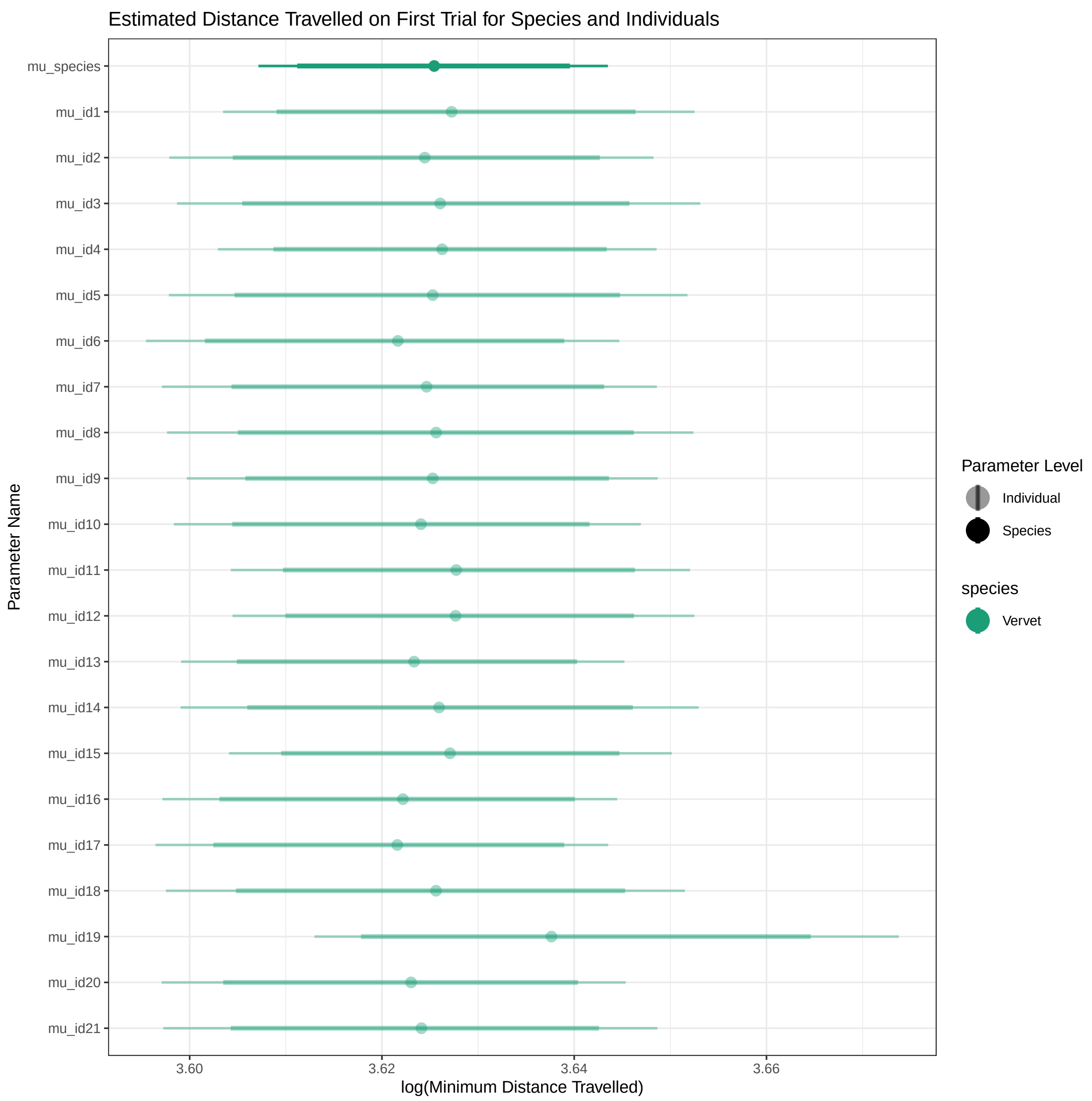


```
#plot slope (b) paramters
b_posterior_plot <- posterior_quantiles_Pentagon %>%
  filter(param_type %in% c("b_species", "b_id")) %>%
  arrange(species) %>%
  mutate(parameter = factor(parameter, levels = parameter[length(parameter):1])) %>%
  ggplot(aes(y = parameter)) +
  geom_pointrange(aes(x = X50,
                      xmin = X10,
                      xmax = X90,
                      alpha = param_type,
                      color = species),
                  orientation = "y", size = 1.5, fatten = 1.5) +
  geom_linerange(aes(xmin = X5,
                     xmax = X95,
                     alpha = param_type,
                     color = species),
                 orientation = "y", size = 0.8) +
```

```
  scale_alpha_discrete(range = c(.4, 1), name = "Parameter Level", labels = c("Individual", "Species")) +
  scale_color_brewer(palette = "Dark2") +
  geom_vline(xintercept = 0, color = "red", linetype = "dashed") +
  theme_bw() +
  theme(legend.key.size = unit(0.75, "cm")) +
  labs(x = "log(Distance)/log(Trial)",
       y = "Slope Parameter",
       title = "Estimated Learning Rates for Species and Individuals")

print(b_posterior_plot)
```

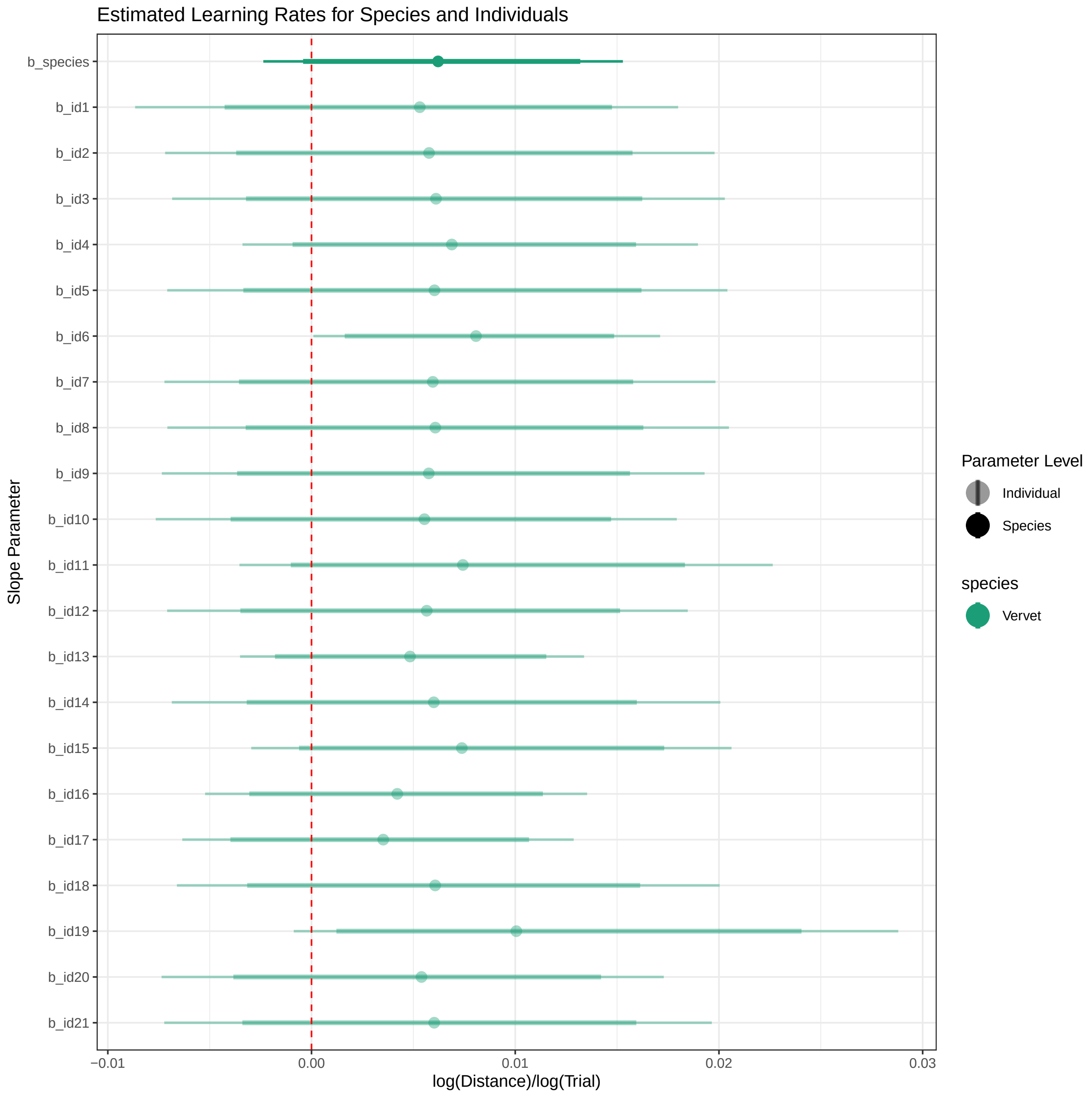


```
## pdf
##   2

## pdf
```

```
## 2
```

Finally, we plot a mean regression line (thick) and 100 sampled regression lines (thin) through the data for each species.

```
Trial <- 1:max(Pentagon_data$Trial)
pal <- brewer.pal(n = 8, name = "Dark2")
species <- c("Vervet")
sample_set <- sample(1:4000, size = 100)

#for manuscript
tiff("../Docs/Supp_Figure 9_Posterior_Predictions_Pentagon.tif", width = 1600, height = 1200)
par(mfrow = c(2,2),
    mar = c(6,8,6,6))
for (i in 1:1) { #loop over each species (column in posterior estimates)
  plot(1, type = "n", xlim = c(0, max(Pentagon_data$Trial_scaled)), ylim = c(3.5,4), ylab = NA, xlab = N
  title(ylab = "log(Distance)", xlab = "log(Trial)", cex.lab = 3, line = 4)
  Distance_mean <- mean(posteriors_Pentagon$mu_species[,i]) + mean(posteriors_Pentagon$b_species[,i]) *
  lines(x = log(Trial), y = Distance_mean, col = pal[i], lwd = 3)
  for (j in sample_set) {
    lines(x = log(Trial), y = posteriors_Pentagon$mu_species[j,i] + posteriors_Pentagon$b_species[j,i] *
  }
  species_data <- filter(Pentagon_data, Species == species[i])
  points(x = species_data$Trial_scaled, y = species_data$Distance_scaled, col = pal[i], pch = 19, cex =
}
dev.off()
```

```
## pdf
## 2
```

```
#for supplement
for (i in 1:1) { #loop over each species (column in posterior estimates)
  plot(1, type = "n", xlim = c(0, max(Pentagon_data$Trial_scaled)), ylim = c(3.5,4), ylab = NA, xlab = N
  title(ylab = "log(Distance)", xlab = "log(Trial)", cex.lab = 1, line = 2)
  Distance_mean <- mean(posteriors_Pentagon$mu_species[,i]) + mean(posteriors_Pentagon$b_species[,i]) *
  lines(x = log(Trial), y = Distance_mean, col = "red", lwd = 3)
  for (j in sample_set) {
    lines(x = log(Trial), y = posteriors_Pentagon$mu_species[j,i] + posteriors_Pentagon$b_species[j,i] *
  }
  points(x = filter(Pentagon_data, Species == levels(Pentagon_data$Species)[i])$Trial_scaled,
         y = filter(Pentagon_data, Species == levels(Pentagon_data$Species)[i])$Distance_scaled,
         pch = 19, cex = 0.5, col = alpha(pal[i], alpha = 0.4))
}
```

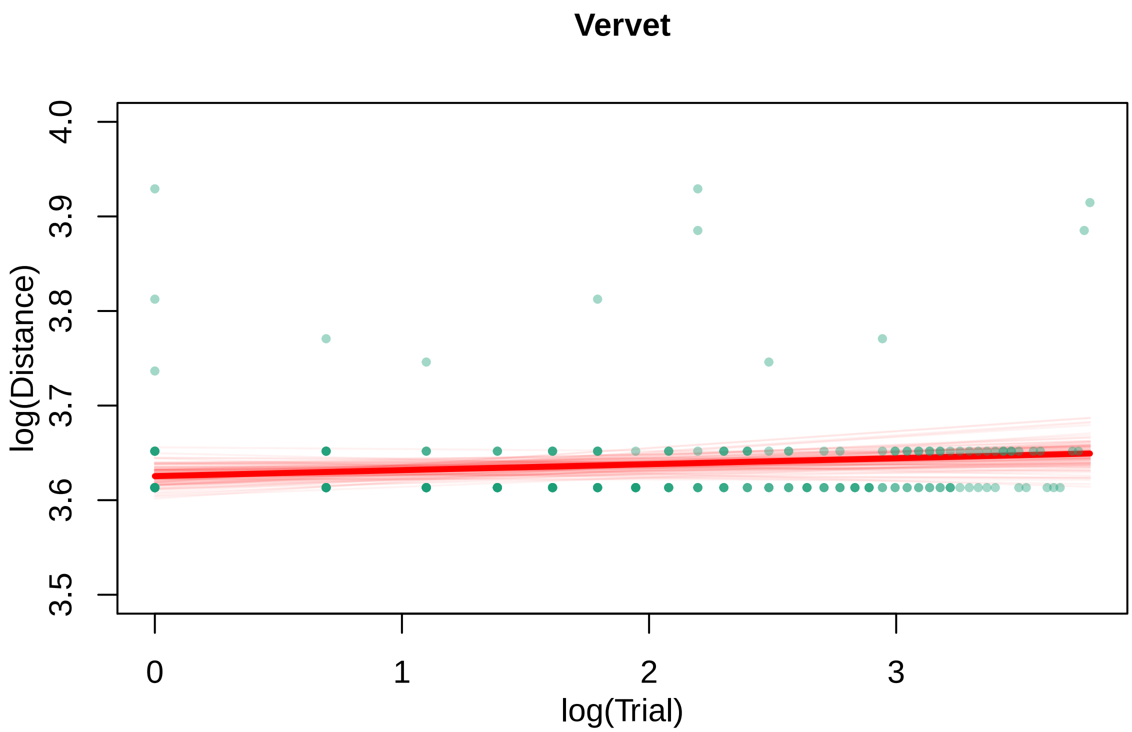

In the below code, we extract a few values for reporting in final publication

```
#slope parameter, vervets, percent of posterior greater than 0
sum(posteriors_Pentagon$b_species[,1] >= 0)/length(posteriors_Pentagon$b_species[,1])
```

```
## [1] 0.885
```

Z-Array Analysis

We analyze data from the Z-Array, which was completed by vervets and japanese macaques, in the same manner as the Double Trapezoid and the Pentagon.

```
#remove simulation data, data from arrays other than Z-Array, and any individuals that completed fewer
Zed_data <- data_cleaned %>%
  filter(Source == "Experimental", Array == "Zarray", max(Trial) >= 5)

Zed_data$Species <- factor(Zed_data$Species)
Zed_data$ID <- factor(Zed_data$ID)

#visualize the data and restructure for model fitting in STAN
ggplot(Zed_data) + geom_jitter(aes(x = Trial_scaled, y = Distance_scaled), alpha = 0.6) +
  facet_wrap(~Species)
```

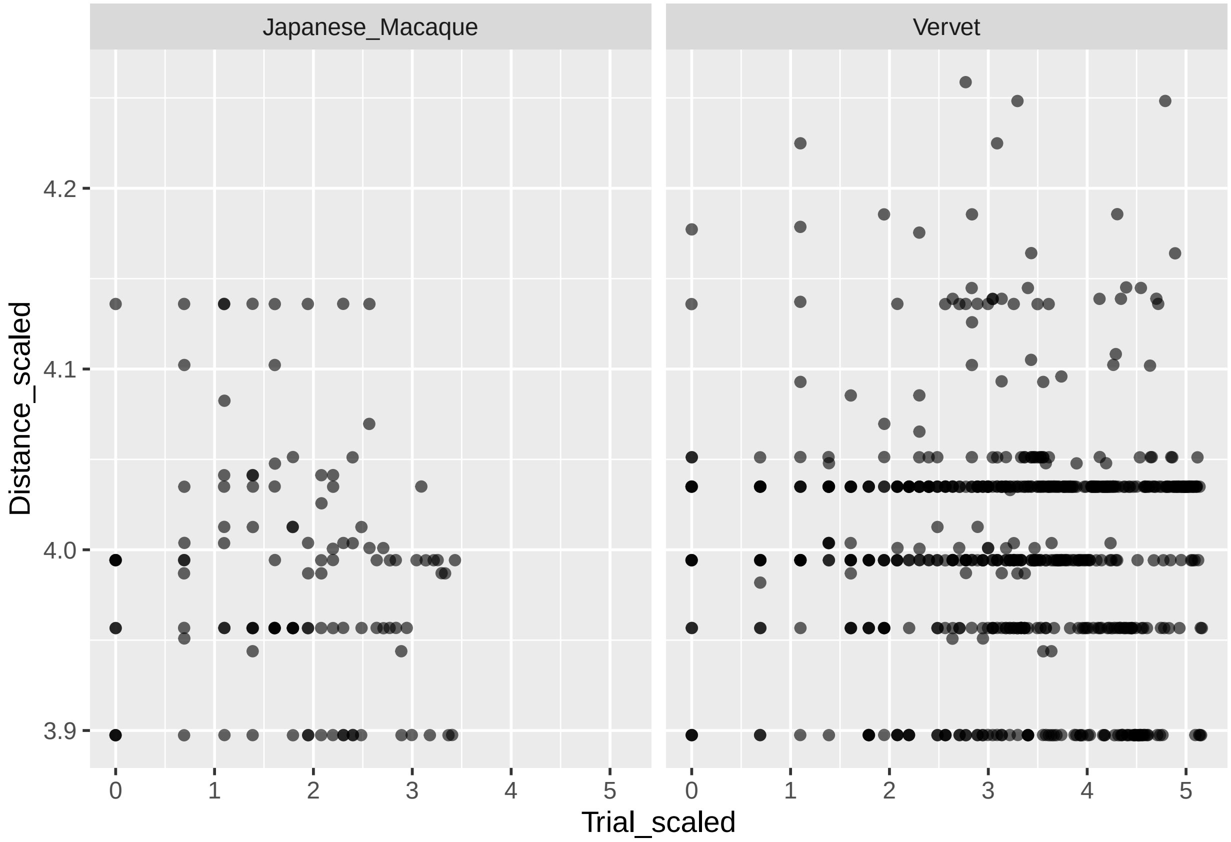


```r
stan_data_Zed <- list(N = nrow(Zed_data),
                      J = length(unique(Zed_data$ID)),
                      K = length(unique(Zed_data$Species)),
                      y = Zed_data$Distance_scaled,
                      x = Zed_data$Trial_scaled,
                      id = as.numeric(Zed_data$ID),
                      sp = as.numeric(Zed_data$Species))
```

```r
options(mc.cores = parallel::detectCores())
fit_Zed <- stan(file = "../CODE/DT_power_model.stan",
             data = stan_data_Zed,
             chains = 4,
             iter = 8000,
             warmup = 4000,
             control = list(max_treedepth = 15))

save(fit_Zed, file = "../Results/power_model_Zed.Rdata")
```

Model Diagnostics

```r
load("../Results/power_model_Zed.Rdata")

#summary of results
```

```r
print(fit_Zed, pars=c("mu_0", "mu_species", "b_0",
                      "b_species", "sigma_e", "lp__"), probs = c(.1, .5,.9))
```

```
## Inference for Stan model: DT_power_model.
## 4 chains, each with iter=8000; warmup=4000; thin=1;
## post-warmup draws per chain=4000, total post-warmup draws=16000.
##
##                  mean se_mean    sd     10%     50%     90% n_eff Rhat
## mu_0             4.27    0.01  0.58    3.54    4.26    5.02  7398    1
## mu_species[1]   -0.26    0.01  0.58   -1.01   -0.26    0.47  7396    1
## mu_species[2]   -0.26    0.01  0.58   -1.01   -0.26    0.47  7391    1
## b_0              0.00    0.01  0.58   -0.74    0.00    0.74  6861    1
## b_species[1]    -0.01    0.01  0.58   -0.75   -0.01    0.73  6862    1
## b_species[2]     0.00    0.01  0.58   -0.74    0.00    0.74  6857    1
## sigma_e[1]       0.10    0.00  0.01    0.09    0.10    0.11 17021    1
## sigma_e[2]       0.13    0.00  0.00    0.12    0.13    0.13 20737    1
## lp__          1903.85    1.06 19.29 1882.86 1900.72 1929.61   334    1
##
## Samples were drawn using NUTS(diag_e) at Thu Aug 26 00:50:36 2021.
## For each parameter, n_eff is a crude measure of effective sample size,
## and Rhat is the potential scale reduction factor on split chains (at
## convergence, Rhat=1).
```

```r
traceplot(fit_Zed, pars = c("mu_0", "mu_species", "b_0", "b_species", "lp__"), inc_warmup = TRUE) #chec
```

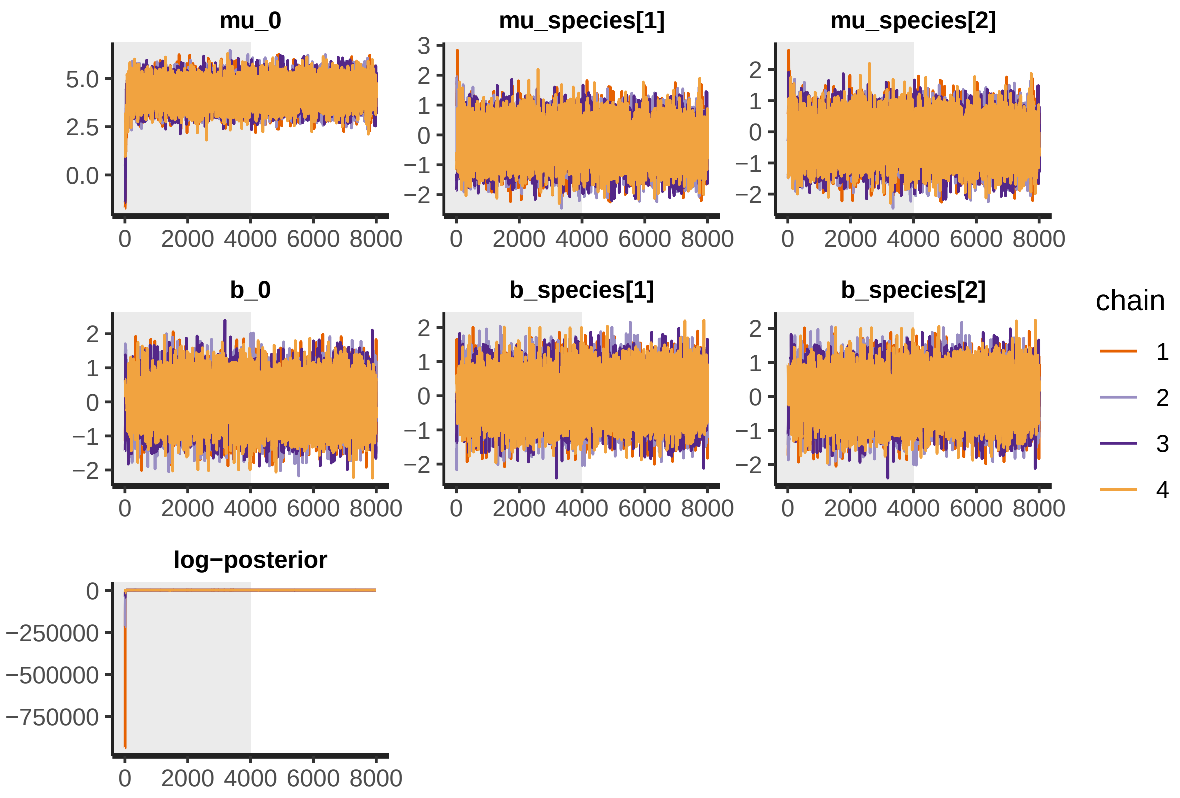

```r
#look for estimation anomalies:
pairs(fit_Zed, pars = c("mu_0", "mu_species", "sigma_e"), las = 1)
```

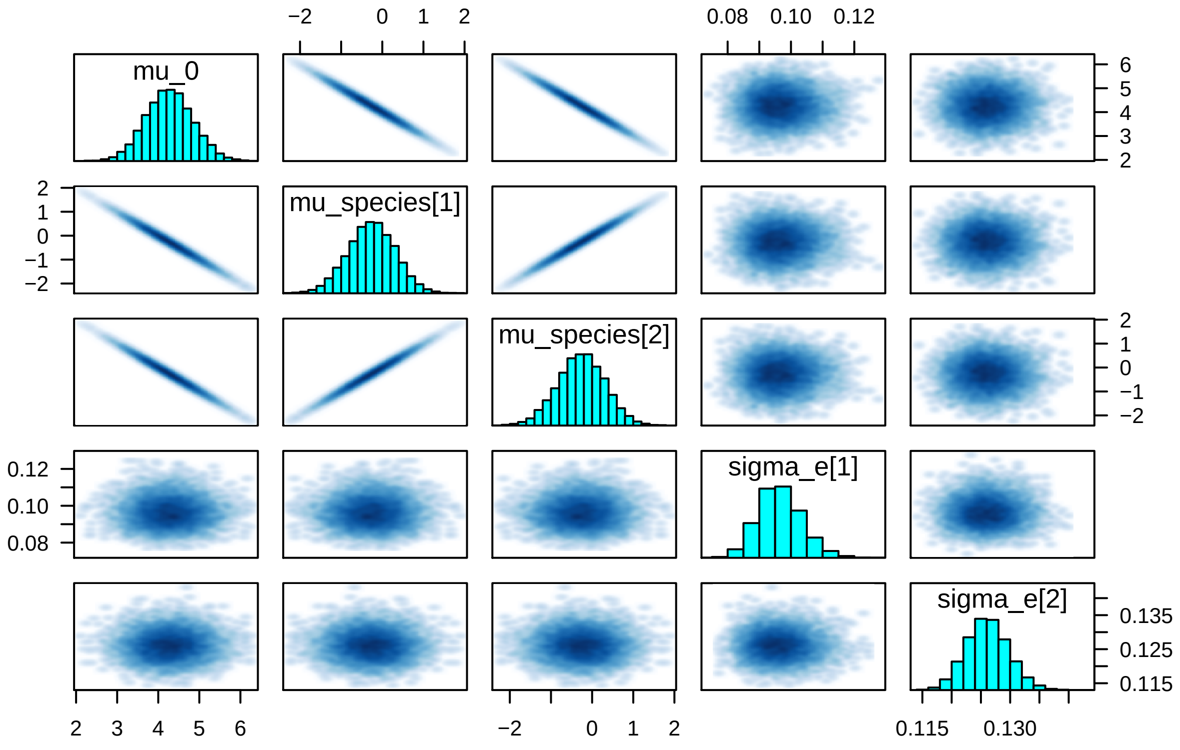


```r
pairs(fit_Zed, pars = c("b_0", "b_species", "sigma_e"), las = 1)
```

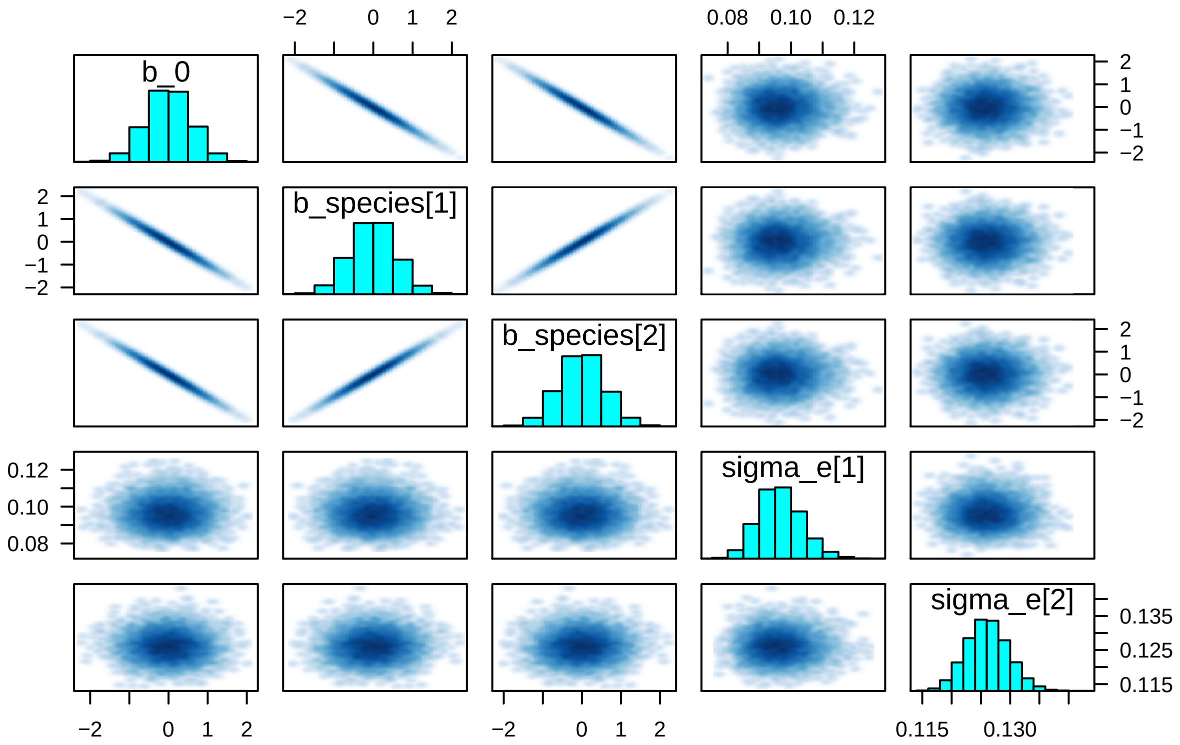


```r
pairs(fit_Zed, pars = c("mu_0", "mu_species", "b_0", "b_species", "lp__"), las = 1)
```

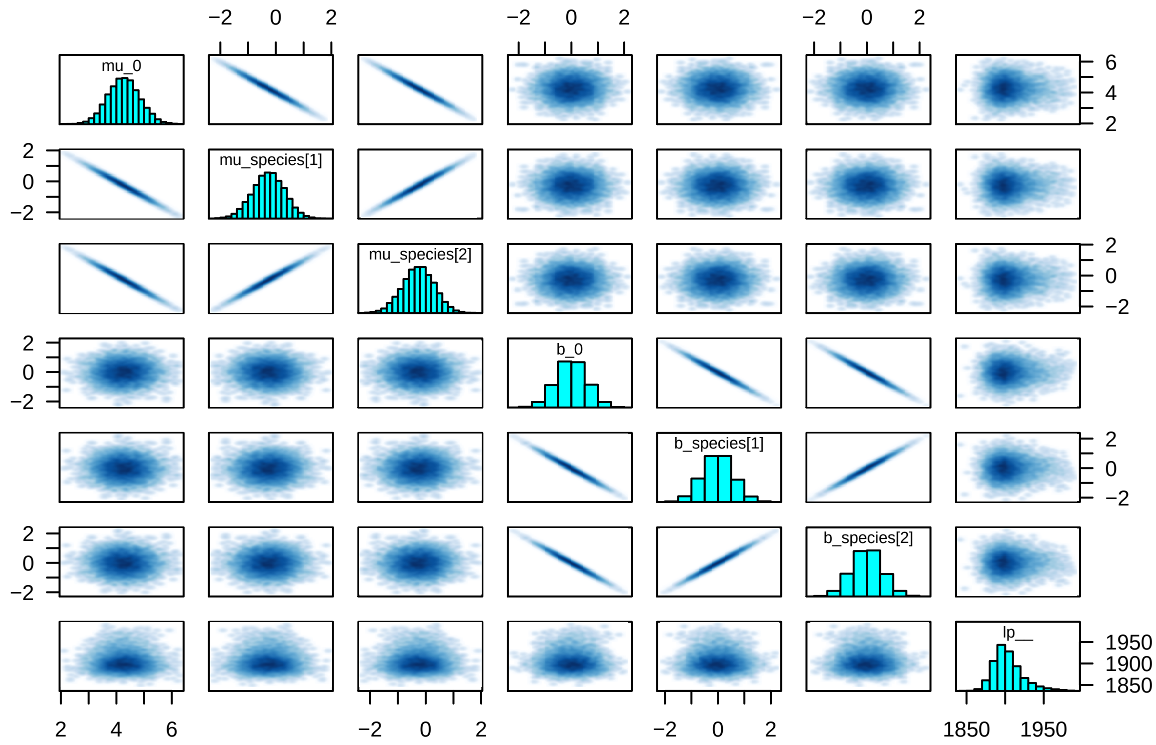


```r
#all posteriors are normally distributed, with appropriate levels of correlation in expected places
```

```r
#residual analysis
r_bar <- apply(extract(fit_Zed, "pearson_residual")[[1]], MARGIN = 2, mean)
y_pred <- apply(extract(fit_Zed, "y_pred")[[1]], MARGIN = 2, mean)
plot(x = y_pred, y = r_bar) #residuals against prediction
plot(x = stan_data_Zed$x, y = r_bar)
plot(x = stan_data_Zed$sp, y = r_bar)
qqnorm(r_bar)
```

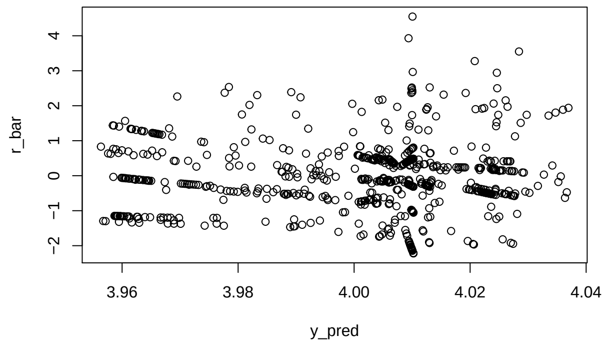

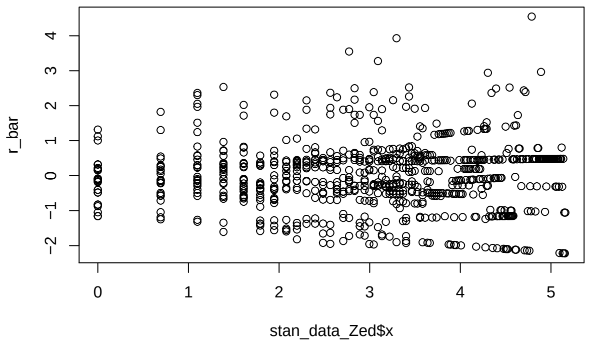

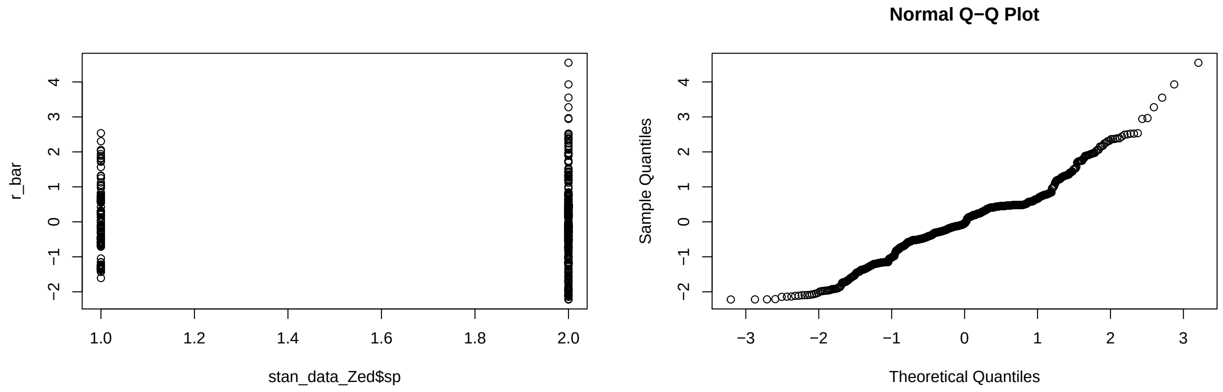

MCMC chains show converging and good mixing. Parameter distributions in the posterior are normal with no unexpected correlations. Residuals show a sligh downward trend with predicted distance traveled, but it is small enough not to be concerning.

Model Interpretation

Because we are estimating the grand mean for the intercept and slope of the model and then estimating species and individual level *deviations*, we need to add the deviations to the grand means for each sample to find the actual posterior estimates of the intercept and slope for each species/individual.

```
posteriors_Zed <- extract(fit_Zed)
posteriors_Zed$mu_species <- apply(posteriors_Zed[["mu_species"]], MARGIN = 2, FUN = function(X) X + pos
posteriors_Zed$b_species <- apply(posteriors_Zed[["b_species"]], MARGIN = 2, FUN = function(X) X + poste

for(i in 1:ncol(posteriors_Zed[["mu_id"]])){
  sp <- Zed_data$Species[match(levels(Zed_data$ID), Zed_data$ID)][i] #get species of id factor level i
  posteriors_Zed[["mu_id"]][,i] <- posteriors_Zed[["mu_species"]][,sp] + posteriors_Zed[["mu_id"]][,i]
  posteriors_Zed[["b_id"]][,i] <- posteriors_Zed[["b_species"]][,sp] + posteriors_Zed[["b_id"]][,i]  #a
}
```

To visualize the resulting posteriors, we determine quantiles of each posterior distribution and plot the 80% (thick bars) and 90% (thin bars) confidence intervals for each species and individual level parameter.

```
#function to get quantiles from each column of a matrix (corresponding to the posterior distribution of a variable)
apply_quant <- function(x) apply(as.matrix(x),
                                 MARGIN = 2,
                                 quantile, c(0.025, .05, .10, .5, .90, .95, 0.975))

#get quantiles for the first 8 parameters in "posteriors" list
posteriors_Zed[1:8] %>% lapply(FUN = apply_quant) %>%
  #bind list of quanitile vectors into a matrix
  do.call(args = ., what = cbind) %>%
  #transpose and save as dataframe
  t() %>% as.data.frame() -> posterior_quantiles_Zed

#remove % and start all names with a character to avoid trouble in ggplot
colnames(posterior_quantiles_Zed) <- paste("X", gsub("%", "", colnames(posterior_quantiles_Zed)), sep = "")

#add metadata to describe each parameter with quantiles
#gets a vector named with parameter names for each type of parameter, then extracts and concatenates those names.
posterior_quantiles_Zed$parameter <- names(unlist(sapply(posteriors_Zed[1:8],
                                                 function(X) seq(to = ncol(as.matrix(X))))))
posterior_quantiles_Zed$param_type <- rep(names(posteriors_Zed[1:8]),
```

```r
                                      times = sapply(posteriors_Zed[1:8],
                                                     function(X) ncol(as.matrix(X))))

#~~give species metadata for parameters where relevant
posterior_quantiles_Zed$species <- NA
posterior_quantiles_Zed$species[posterior_quantiles_Zed$param_type == "mu_id"] <-
  as.character(Zed_data$Species[match(levels(Zed_data$ID), Zed_data$ID)])
posterior_quantiles_Zed$species[posterior_quantiles_Zed$param_type == "b_id"] <-
  as.character(Zed_data$Species[match(levels(Zed_data$ID), Zed_data$ID)])
posterior_quantiles_Zed$species[posterior_quantiles_Zed$param_type == "mu_species"] <-
  levels(Zed_data$Species)
posterior_quantiles_Zed$species[posterior_quantiles_Zed$param_type == "b_species"] <-
  levels(Zed_data$Species)
#~~~~~~~~~~
```

```r
#plot intercept(mu) parameters
mu_posterior_plot <- posterior_quantiles_Zed %>%
  filter(param_type %in% c("mu_species", "mu_id")) %>%
  arrange(species) %>%
  mutate(parameter = factor(parameter, levels = parameter[length(parameter):1])) %>%
  ggplot(aes(y = parameter)) +
  geom_pointrange(aes(x = X50,
                      xmin = X10,
                      xmax = X90,
                      color = species,
                      alpha = param_type),
                  orientation = "y", size = 1.5, fatten = 1.5) +
  geom_linerange(aes(xmin = X5,
                     xmax = X95,
                     color = species,
                     alpha = param_type),
                 orientation = "y", size = 0.8) +
  scale_alpha_discrete(range = c(.4, 1), name = "Parameter Level", labels = c("Individual", "Species")) +
  scale_color_brewer(palette = "Dark2") +
  theme_bw() +
  theme(legend.key.size = unit(0.75, "cm")) +
  labs(x = "log(Minimum Distance Travelled)",
       y = "Parameter Name",
       title = "Estimated Distance Travelled on First Trial for Species and Individuals")

print(mu_posterior_plot)
```

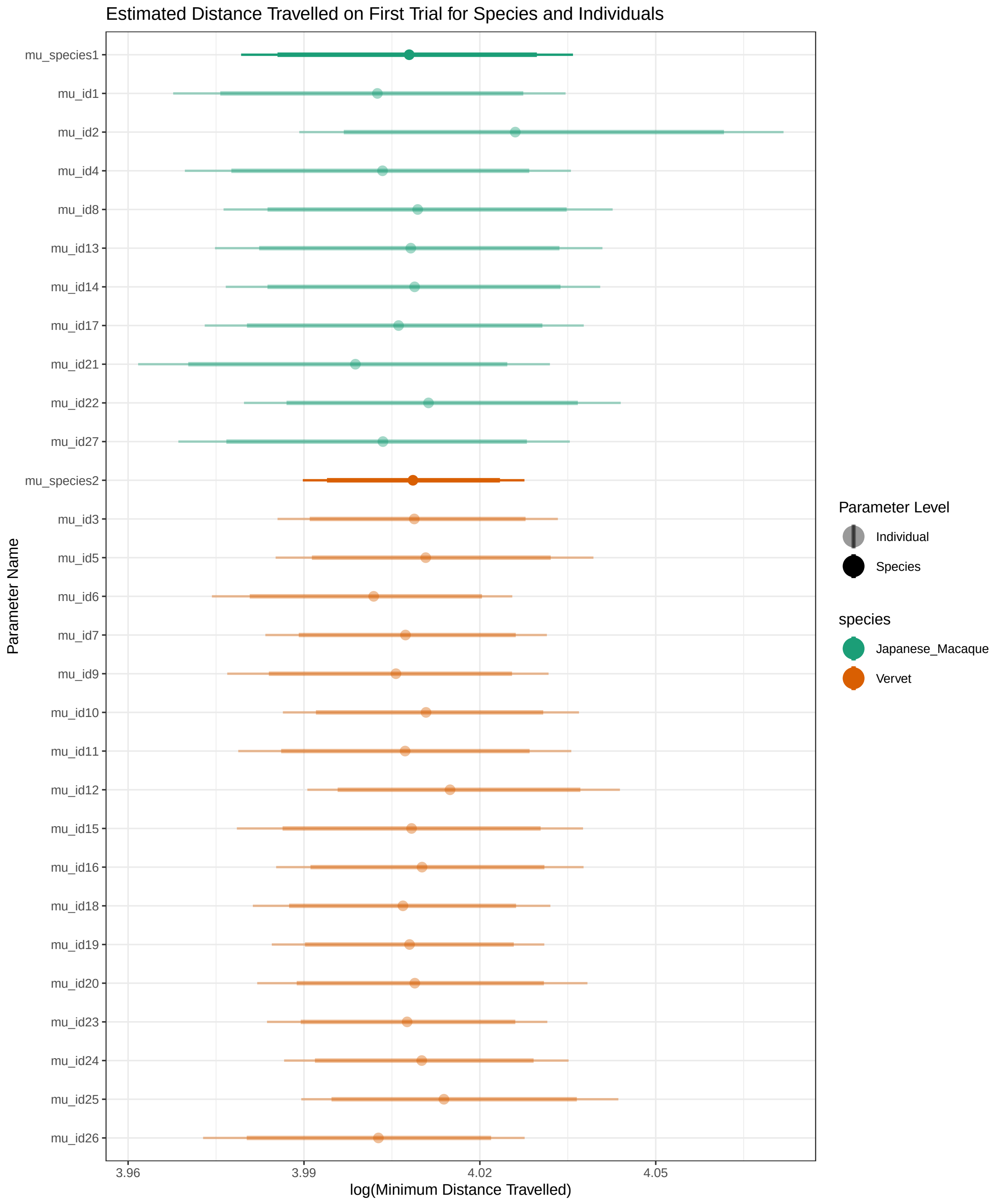


```
#plot slope (b) paramters
b_posterior_plot <- posterior_quantiles_Zed %>%
  filter(param_type %in% c("b_species", "b_id")) %>%
  arrange(species) %>%
  mutate(parameter = factor(parameter, levels = parameter[length(parameter):1])) %>%
  ggplot(aes(y = parameter)) +
  geom_pointrange(aes(x = X50,
                      xmin = X10,
```

```
                      xmax = X90,
                      alpha = param_type,
                      color = species),
                  orientation = "y", size = 1.5, fatten = 1.5) +
  geom_linerange(aes(xmin = X5,
                     xmax = X95,
                     alpha = param_type,
                     color = species),
                 orientation = "y", size = 0.8) +
  scale_alpha_discrete(range = c(.4, 1), name = "Parameter Level", labels = c("Individual", "Species")) +
  scale_color_brewer(palette = "Dark2") +
  geom_vline(xintercept = 0, color = "red", linetype = "dashed") +
  theme_bw() +
  theme(legend.key.size = unit(0.75, "cm")) +
  labs(x = "log(Distance)/log(Trial)",
       y = "Slope Parameter",
       title = "Estimated Learning Rates for Species and Individuals")

print(b_posterior_plot)
```

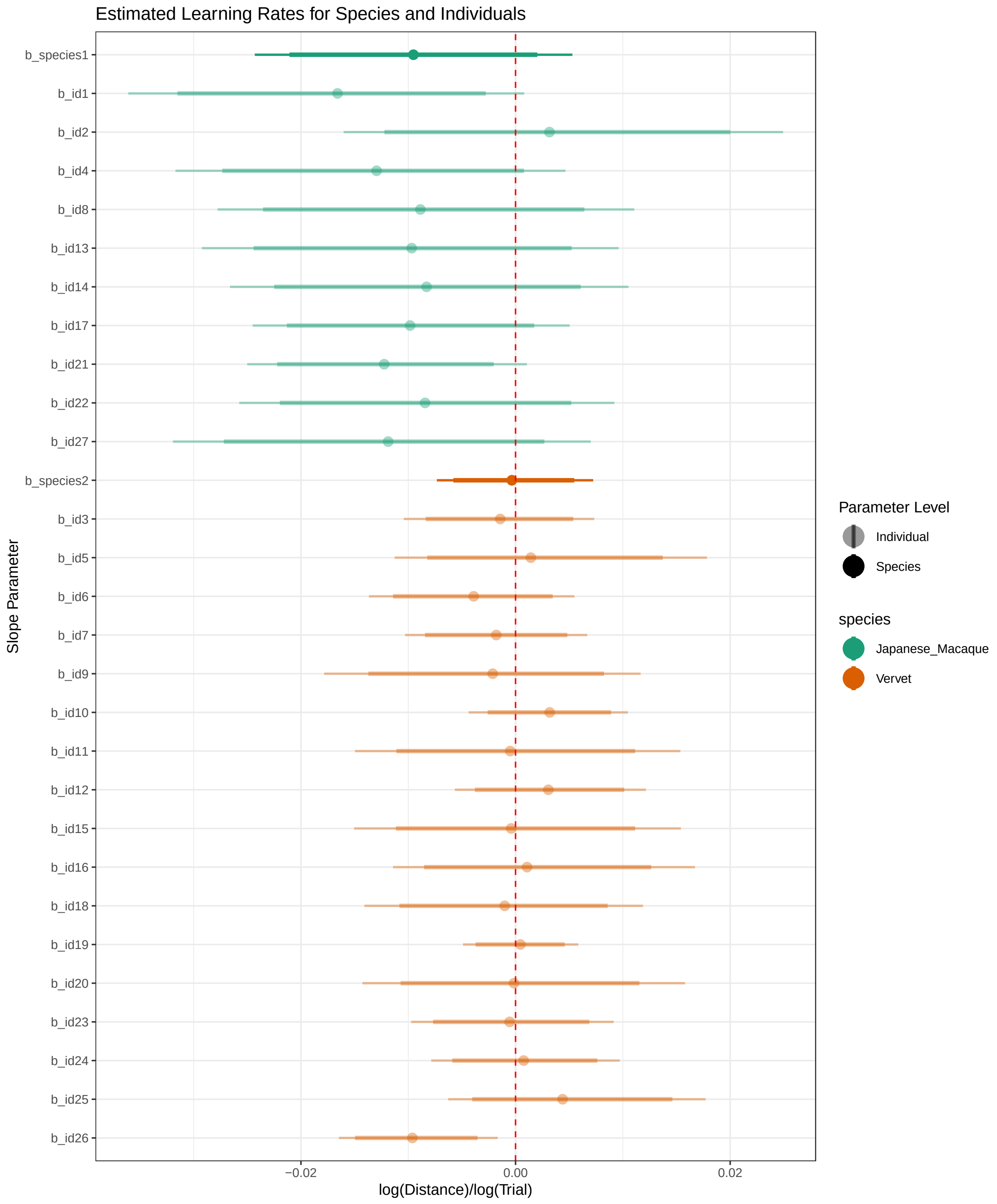

Estimated Learning Rates for Species and Individuals
b_species1
b_id1
b_id2
b_id4
b_id8
b_id13
b_id14
b_id17
b_id21
b_id22
b_id27
b_species2
b_id3
b_id5
b_id6
b_id7
b_id9
b_id10
b_id11
b_id12
b_id15
b_id16
b_id18
b_id19
b_id20
b_id23
b_id24
b_id25
b_id26
Slope Parameter
−0.02
0.00
0.02
log(Distance)/log(Trial)
Parameter Level
Individual
Species
species
Japanese_Macaque
Vervet


```
## pdf
##   2

## pdf
##   2
```

Finally, we plot a mean regression line (thick) and 100 sampled regression lines (thin) through the data for each species.

```
Trial <- 1:max(Zed_data$Trial)
pal <- brewer.pal(n = 8, name = "Dark2")
species <- c("Japanese_Macaque", "Vervet")
sample_set <- sample(1:4000, size = 100)

#Figure for Manuscript
tiff("../Docs/Supp_Figure 14_Posterior_Predictions_Zed.tif", width = 1600, height = 1200)
par(mfrow = c(2,2),
    mar = c(6,8,6,6))
for (i in 1:2) { #loop over each species (column in posterior estimates)
  plot(1, type = "n", xlim = c(0, max(Zed_data$Trial_scaled)), ylim = c(3.8,4.5), ylab = NA, xlab = NA,
  title(ylab = "log(Distance)", xlab = "log(Trial)", cex.lab = 3, line = 4)
  Distance_mean <- mean(posteriors_Zed$mu_species[,i]) + mean(posteriors_Zed$b_species[,i]) * log(Trial
  lines(x = log(Trial), y = Distance_mean, col = pal[i], lwd = 3)
  for (j in sample_set) {
    lines(x = log(Trial), y = posteriors_Zed$mu_species[j,i] + posteriors_Zed$b_species[j,i] * log(Tria
  }
  species_data <- filter(Zed_data, Species == species[i])
  points(x = species_data$Trial_scaled, y = species_data$Distance_scaled, col = pal[i], pch = 19, cex =
}
dev.off()
```

```
## pdf
##   2
```

```
#Figure for Supplement
for (i in 1:2) { #loop over each species (column in posterior estimates)
  plot(1, type = "n", xlim = c(0, max(Zed_data$Trial_scaled)), ylim = c(3.8,4.5), ylab = NA, xlab = NA,
  title(ylab = "log(Distance)", xlab = "log(Trial)", cex.lab = 1, line = 2)
  Distance_mean <- mean(posteriors_Zed$mu_species[,i]) + mean(posteriors_Zed$b_species[,i]) * log(Trial
  lines(x = log(Trial), y = Distance_mean, col = "red", lwd = 3)
  for (j in sample_set) {
    lines(x = log(Trial), y = posteriors_Zed$mu_species[j,i] + posteriors_Zed$b_species[j,i] * log(Tria
  }
  points(x = filter(Zed_data, Species == levels(Zed_data$Species)[i])$Trial_scaled,
         y = filter(Zed_data, Species == levels(Zed_data$Species)[i])$Distance_scaled,
         pch = 19, cex = 0.5, col = alpha(pal[i], alpha = 0.4))
}
```

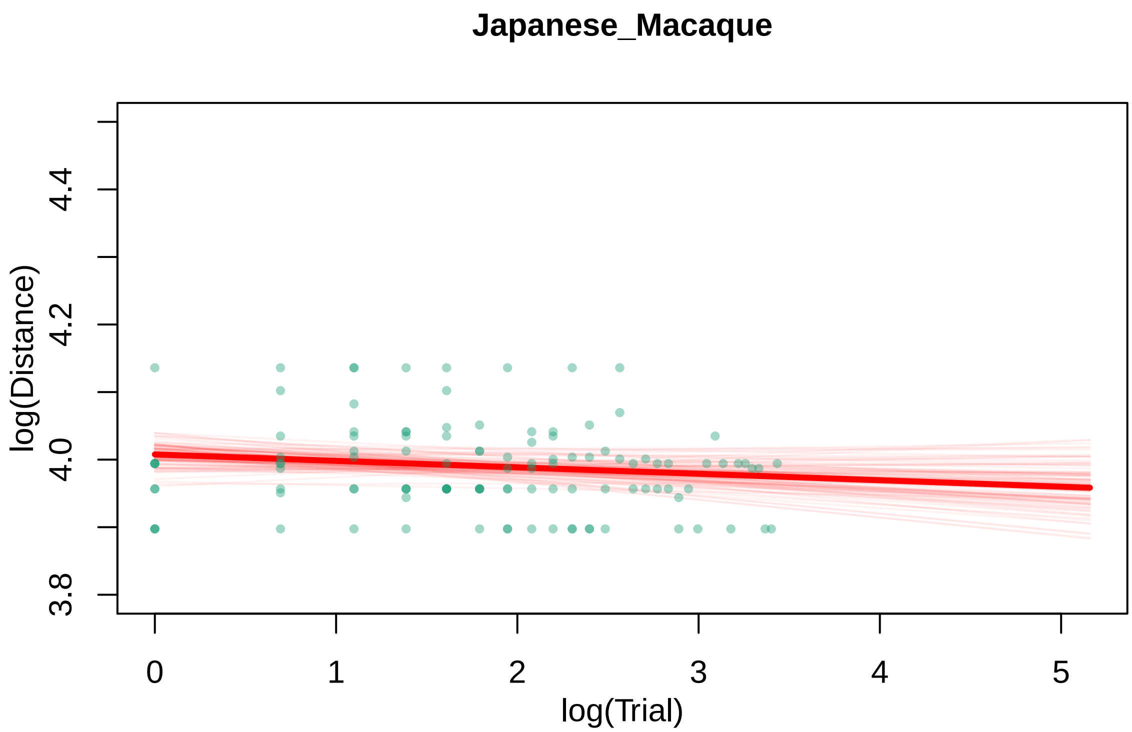
Japanese_Macaque
log(Distance)
3.8
4.0
4.2
4.4
0
1
2
3
4
5
log(Trial)

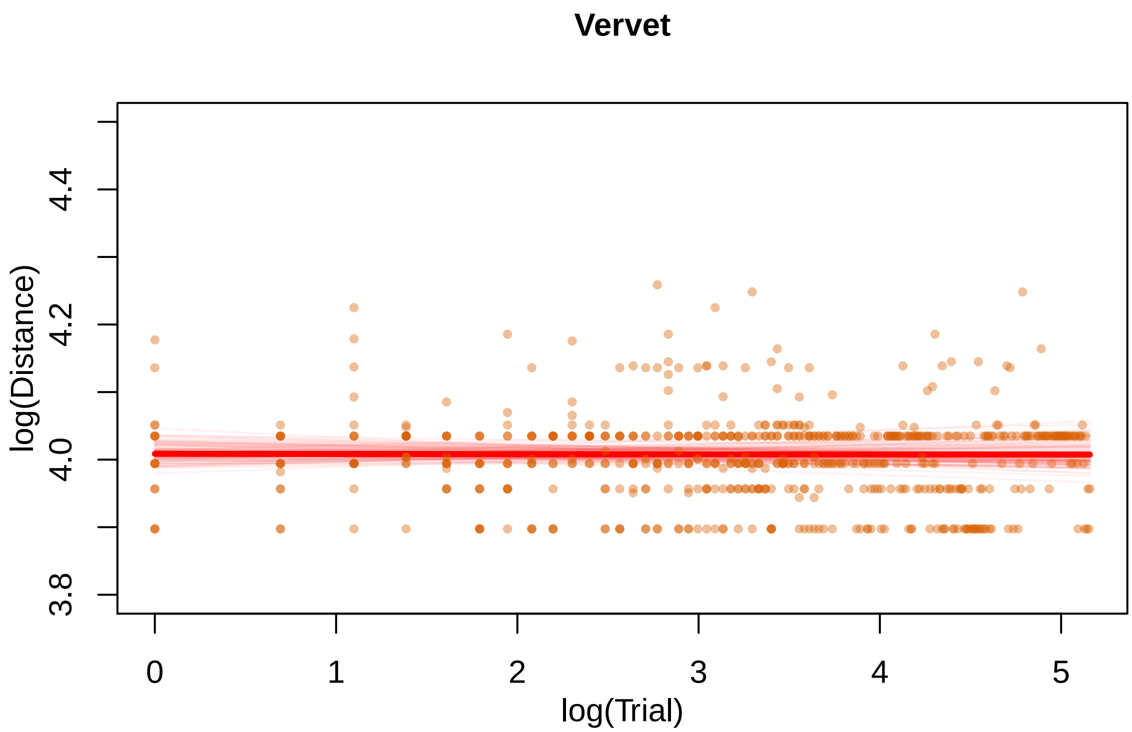

Below, we calculate a few additional values for final publication

```
sum(posteriors_Zed$b_species[,1] <= 0)/length(posteriors_Zed$b_species[,1])
```

```
## [1] 0.855
```

Simulation analysis, Double Trapezoid

We start by analyzing simulations from the Double Trapezoid array, in which the most species of primates were tested and thus offers the most potential for comparative analysis.

```
#Get simulated (species == "NONE") double trapezoid data,
#remove individuals with fewer than 5 trials, and treat reinforcement factor (Source) as Species
DT_data_sim <- data_cleaned %>%
  filter(Species == "None", Array == "DT", max(Trial) >= 5) %>% mutate(Species = Source)

DT_data_sim$Species <- factor(DT_data_sim$Species,
                              levels = c("Reinforcement_Factor1", "Reinforcement_Factor1.2", "Reinforcement_Factor2"))

#downsample simulations to reduce computational time of statistical model
DT_data_sim <- DT_data_sim %>% filter(as.numeric(ID) %% 2 == 0)

DT_data_sim$ID <- factor(DT_data_sim$ID)

#visualize raw data
ggplot(DT_data_sim) + geom_point(aes(x = Trial_scaled, y = Distance_scaled), alpha = 0.1) +
  facet_wrap(~Species)
```

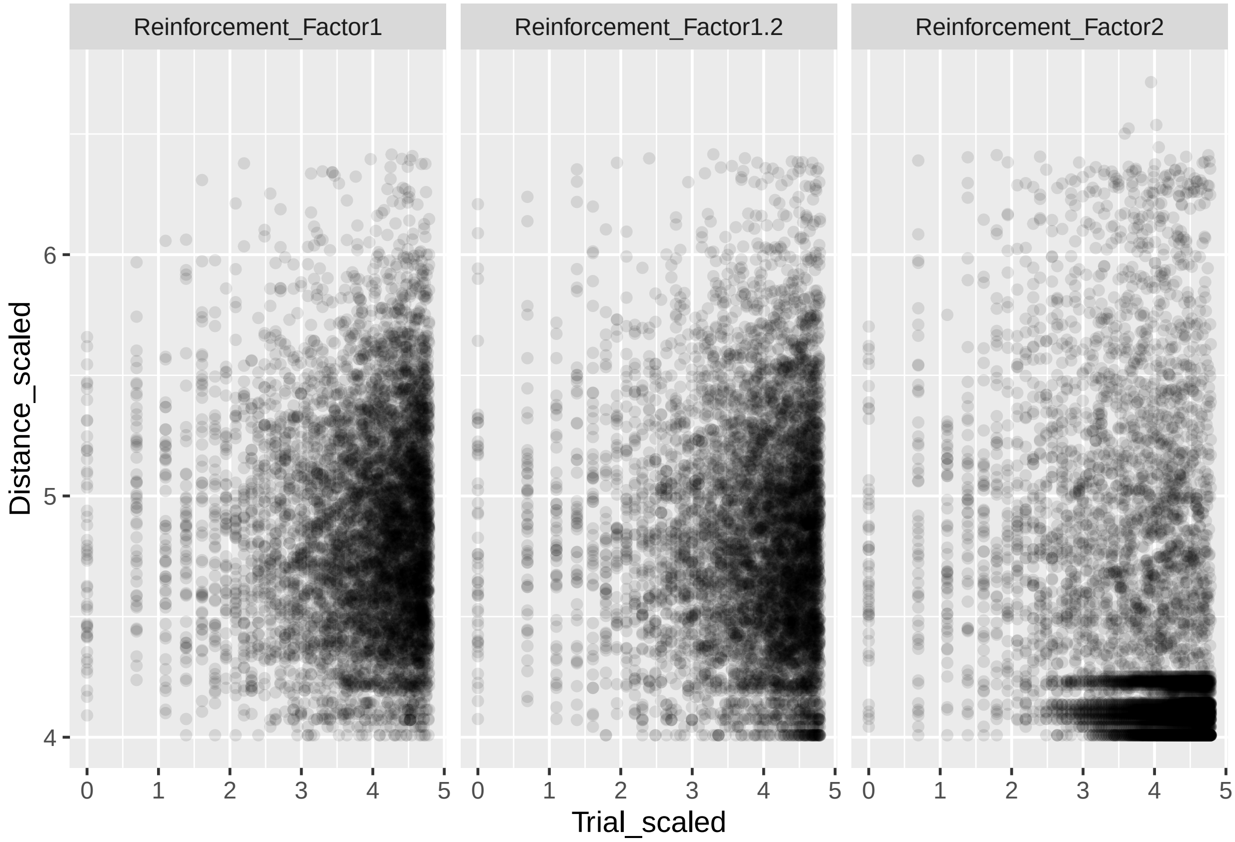


```
#Restructure data for usage in STAN (see "DT_power_model.stan" for more details)
stan_data_sim_DT <- list(N = nrow(DT_data_sim),
                         J = length(unique(DT_data_sim$ID)),
                         K = length(unique(DT_data_sim$Species)),
                         y = DT_data_sim$Distance_scaled,
                         x = DT_data_sim$Trial_scaled,
                         id = as.numeric(factor(DT_data_sim$ID)),
                         sp = as.numeric(DT_data_sim$Species))
```

Before running the analysis, we can already see from the raw data that simulations with a Reinforcement Factor of 2 begin to converge of usage of routes with relatively low total travel distance around e^2.5 (~12) trials, though a subset of individuals remain highly varied in their performance. No such trend is immediately visible in data from simulations with Reinforcement Factors of 1 or 1.2. To better see the heterogeneity in individual performance, especially in Reinforcement Factor 2 agents, we add lines to the data for a subset of these individuals

```
DT_data_sim_subset <- DT_data_sim %>% filter(ID %in% sample(unique(DT_data_sim$ID), 15))

ggplot(DT_data_sim_subset, aes(x = Trial_scaled, y = Distance_scaled, color = ID)) +
  geom_point(size = 0.7) +
  geom_line(alpha = 0.5) +
  facet_wrap(~Species)
```

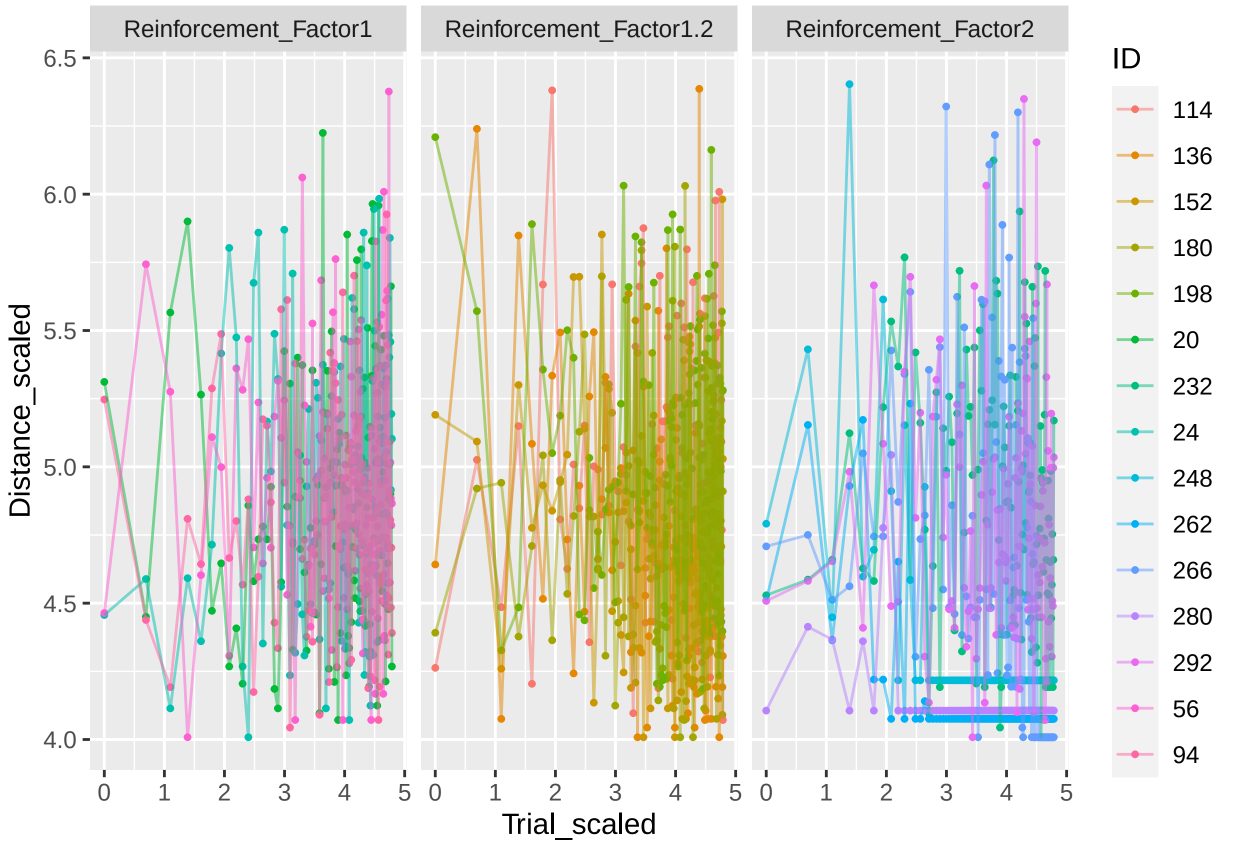

Having gained a sense of the patterns in these simulations, we analyze them using the same statistical model as used for the empirical data. The final STAN properties used in the following code block we adjusted several times to achieve convergence and mixing in the MCMC chains.

```
options(mc.cores = parallel::detectCores())
fit_sim <- stan(file = "../CODE/DT_power_model.stan",
             data = stan_data_sim_DT,
             chains = 4,
             warmup = 3000,
             iter = 6000,
             control = list(max_treedepth = 15)
             )

save(fit_sim, file = "../Results/power_model_fit1.Rdata")
```

Our first step after computing model parameters in STAN is to visualize diagnostics of well the model fit the data.

```
load("../Results/power_model_fit1.Rdata")
```

```
#summarize results
print(fit_sim, pars=c("mu_0", "mu_species", "b_0", "b_species", "sigma_mu_id",
                      "sigma_b_id", "sigma_e", "lp__"),
      probs = c(.1, .5,.9))
```

```
## Inference for Stan model: DT_power_model.
```

```
## 4 chains, each with iter=6000; warmup=3000; thin=1;
## post-warmup draws per chain=3000, total post-warmup draws=12000.
##
##                   mean se_mean    sd     10%     50%     90% n_eff Rhat
## mu_0              4.97    0.01  0.51    4.32    4.98    5.63  9584    1
## mu_species[1]    -0.15    0.01  0.51   -0.81   -0.15    0.50  9553    1
## mu_species[2]    -0.05    0.01  0.51   -0.71   -0.06    0.60  9600    1
## mu_species[3]     0.40    0.01  0.51   -0.26    0.39    1.05  9565    1
## b_0              -0.06    0.01  0.50   -0.69   -0.07    0.59  9458    1
## b_species[1]      0.07    0.01  0.50   -0.58    0.08    0.70  9455    1
## b_species[2]      0.04    0.01  0.50   -0.61    0.04    0.67  9455    1
## b_species[3]     -0.17    0.01  0.50   -0.81   -0.16    0.46  9452    1
## sigma_mu_id       0.18    0.00  0.02    0.15    0.18    0.20  3189    1
## sigma_b_id        0.05    0.00  0.00    0.04    0.05    0.05  3830    1
## sigma_e[1]        0.98    0.00  0.01    0.97    0.98    0.99 28658    1
## sigma_e[2]        1.04    0.00  0.01    1.03    1.04    1.05 30303    1
## sigma_e[3]        0.94    0.00  0.01    0.93    0.94    0.95 22380    1
## lp__           5714.07    0.33 16.38 5692.69 5714.47 5734.66  2482    1
##
## Samples were drawn using NUTS(diag_e) at Fri Sep 17 10:23:14 2021.
## For each parameter, n_eff is a crude measure of effective sample size,
## and Rhat is the potential scale reduction factor on split chains (at
## convergence, Rhat=1).
```

```r
#View parameter estimates across chains
traceplot(fit_sim, pars = c("mu_0", "mu_species", "b_0", "b_species"), inc_warmup = TRUE)

#Look at parameter distributions and correlations
pairs(fit_sim, pars = c("mu_0", "mu_species"), las = 1)
pairs(fit_sim, pars = c("b_0", "b_species"), las = 1)
pairs(fit_sim, pars = c("mu_0", "b_0"), las = 1)

#residual analysis
r_bar <- apply(extract(fit_sim, "pearson_residual")[[1]], MARGIN = 2, mean)
y_pred <- apply(extract(fit_sim, "y_pred")[[1]], MARGIN = 2, mean)
plot(x = y_pred, y = r_bar) #residuals against prediction
plot(x = stan_data_sim_DT$x, y = r_bar)
plot(x = stan_data_sim_DT$sp, y = r_bar)
qqnorm(r_bar)
```

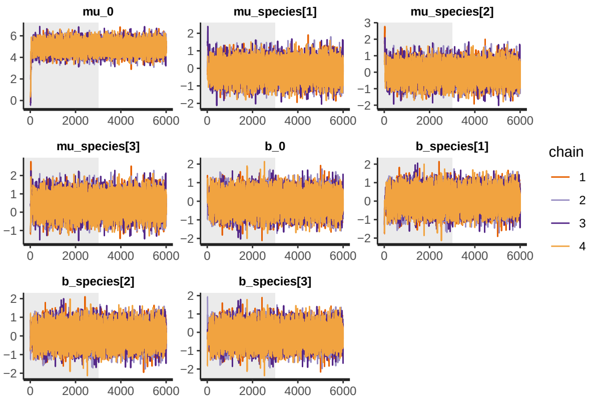

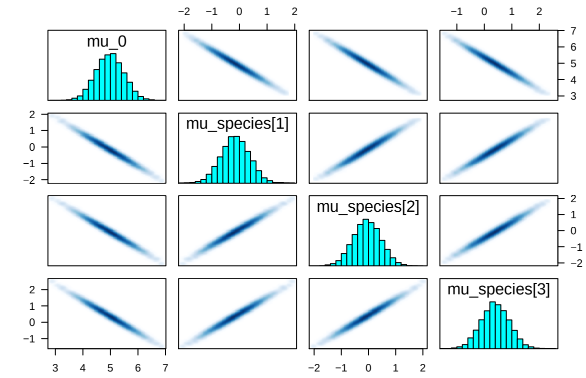

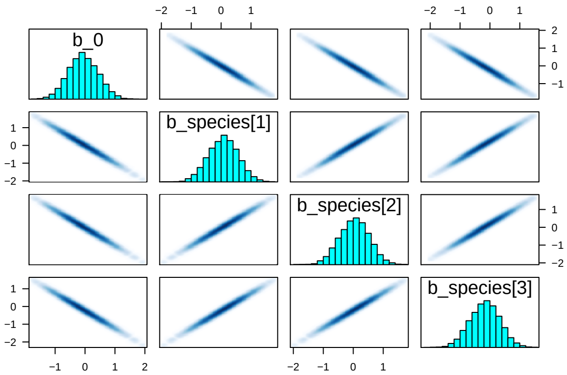

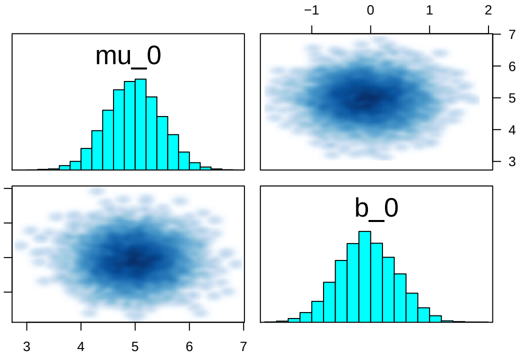

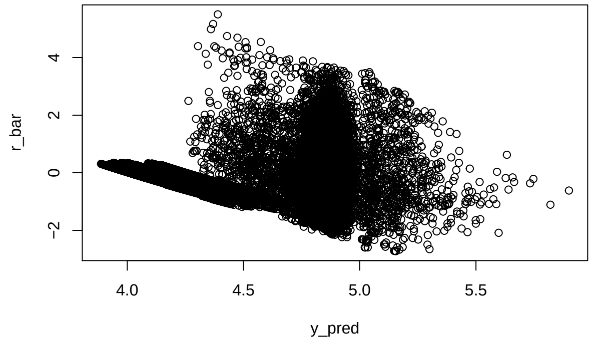

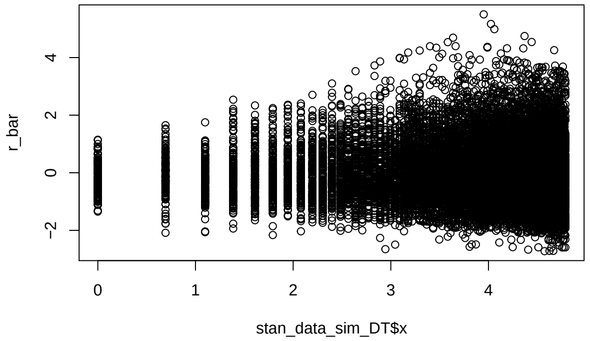

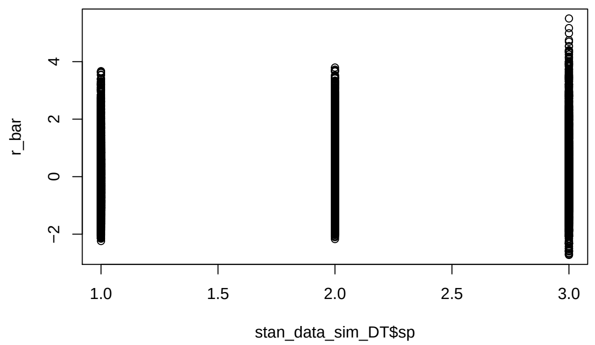

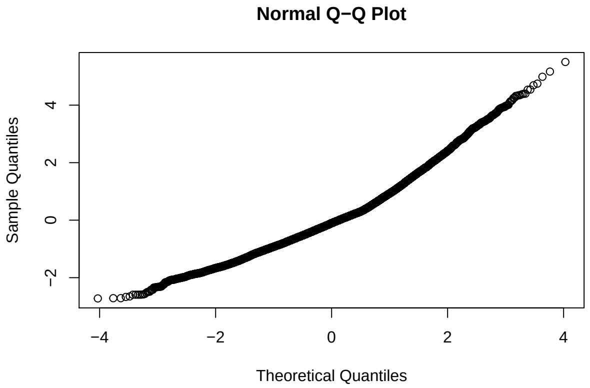

The first thing we note, based on the n_eff and Rhat values from the data summary and the traceplots of the parameter estimates across all four MCMC chains is that our model has converged across chains, and that chains are well mixed. We avoid interpreting the raw reports of parameter values in the data summary, as these don't capture parameter correlations, and most of our values of interest require combining estimated parameters, which we do further below.

The pairs plots indicate a great deal of correlation between the grand mean parameters for intercept and slope, and their corresponding species level adjustments. This is to be expected; as the estimated grand mean parameters move around the true value of species level parameters, estimates of the species level adjustments should change accordingly. Importantly, estimates for the grand mean of the intercept and the grand mean of the slope are not correlated. Additionally, there are not other irregularities in the parameter estimate distributions that would indicate the model was unidentifiable.

The residuals of this model, however, suggest our model does not fit the data well. Looking at residuals

plotted with the predicted value on the x axis, there is a strong negative trend, indicated overestimation is more likely when predictions of distance traveled are low, and vice-versa. Additionally, the variance in residuals is much lower when predicted outcomes are low, reflecting that the convergence of Learning Factor 2 agents on short routes is not well modelled, and perhaps skewing parameter estimates. However, as our goal here is to understand these results in the context of our empirical analysis, we chose not to adjust this model, and instead consider the reasons why a power law function is a good model for actual primate learning in the Double Trapezoid array, but not for "learning" when the iterative reinforcement algorithm operates in the same array.

Thus, we move on by extracting the posteriors distributions of each parameter. First, we calculate species level intercepts and slopes by adding each species level adjustment to the grand means of the appropriate parameters. Then we do the same for individual-level parameter estimates, adding individual-level adjustments to the species level parameters just calculated.

```
posteriors_sim_DT <- extract(fit_sim)

#finds the estimated intercepts by species for each sample - used for plotting estimated learning curves
posteriors_sim_DT$mu_species <- apply(posteriors_sim_DT[["mu_species"]],
                                      MARGIN = 2, FUN = function(X) X + posteriors_sim_DT[["mu_0"]])
posteriors_sim_DT$b_species <- apply(posteriors_sim_DT[["b_species"]],
                                     MARGIN = 2, FUN = function(X) X + posteriors_sim_DT[["b_0"]])

for(i in 1:ncol(posteriors_sim_DT[["mu_id"]])){
  #get species of id factor level i
  sp <- DT_data_sim$Species[match(levels(DT_data_sim$ID), DT_data_sim$ID)][i]
  #add individual deviation to species level intercept
  posteriors_sim_DT[["mu_id"]][,i] <- posteriors_sim_DT[["mu_species"]][,sp] + posteriors_sim_DT[["mu_id"]][,i]
   #add individual deviation to species level slope
  posteriors_sim_DT[["b_id"]][,i] <- posteriors_sim_DT[["b_species"]][,sp] + posteriors_sim_DT[["b_id"]][,i]
}
```

To visualize the resulting posteriors, we first determine quantiles of each posterior distribution and arrange these values in a dataframe for easy plotting.

```
#function to get quantiles from each column of a matrix (corresponding to the posterior distribution of a variable)
apply_quant <- function(x) apply(as.matrix(x), MARGIN = 2, quantile, c(0.025, .05, .10, .5, .90, .95, 0.975))

#get quantiles for the first 8 parameters in "posteriors" list
posteriors_sim_DT[1:8] %>% lapply(FUN = apply_quant) %>%
  #bind list of quantile vectors into a matrix
  do.call(args = ., what = cbind) %>%
  #transpose and save as dataframe
  t() %>% as.data.frame() -> posterior_quantiles_sim_DT

#remove % and start all names with a character to avoid trouble in ggplot
colnames(posterior_quantiles_sim_DT) <- paste("X",
                                              gsub("%", "", colnames(posterior_quantiles_sim_DT)),
                                              sep = "")

#add metadata to describe each parameter with quantiles
#gets a vector named with parameter names for each type of parameter, then extracts and concatenates those names.
posterior_quantiles_sim_DT$parameter <- names(unlist(sapply(posteriors_sim_DT[1:8],
                                                            function(X) seq(to = ncol(as.matrix(X)))
                                                            )))
posterior_quantiles_sim_DT$param_type <- rep(names(posteriors_sim_DT[1:8]),
                                             times = sapply(posteriors_sim_DT[1:8],
                                                            function(X) ncol(as.matrix(X)))
                                             )

#~~give species metadata for parameters where relevant
posterior_quantiles_sim_DT$species <- NA
posterior_quantiles_sim_DT$species[posterior_quantiles_sim_DT$param_type == "mu_id"] <-
  as.character(DT_data_sim$Species[match(levels(DT_data_sim$ID), DT_data_sim$ID)])
posterior_quantiles_sim_DT$species[posterior_quantiles_sim_DT$param_type == "b_id"] <-
```

```r
  as.character(DT_data_sim$Species[match(levels(DT_data_sim$ID), DT_data_sim$ID)])
posterior_quantiles_sim_DT$species[posterior_quantiles_sim_DT$param_type == "mu_species"] <-
  levels(DT_data_sim$Species)
posterior_quantiles_sim_DT$species[posterior_quantiles_sim_DT$param_type == "b_species"] <-
  levels(DT_data_sim$Species)
#~~~~~~~~~~

posterior_quantiles_sim_mu_id_DT <- filter(posterior_quantiles_sim_DT, param_type == "mu_id")
posterior_quantiles_sim_b_id_DT <- filter(posterior_quantiles_sim_DT, param_type == "b_id")

#get a sample of 100 individuals to visualize individual level variance
sample_set_DT <- sample(x = 1:nrow(posterior_quantiles_sim_mu_id_DT), size = 100)

posterior_quantiles_sim_idSubset_DT <- rbind(filter(posterior_quantiles_sim_DT, ! param_type  %in% c("mu_id", "b_id")),
                                          posterior_quantiles_sim_mu_id_DT[sample_set_DT,],
                                          posterior_quantiles_sim_b_id_DT[sample_set_DT,]
                                          )
```

We then plot the 80% (thick bars) and 90% (thin bars) confidence intervals for each species and individual level parameter.

```r
#plot intercept(mu) parameters
mu_posterior_plot <- posterior_quantiles_sim_idSubset_DT %>%
  filter(param_type %in% c("mu_species", "mu_id")) %>%
  arrange(species) %>%
  mutate(parameter = factor(parameter, levels = parameter[length(parameter):1])) %>%
  ggplot(aes(y = parameter)) +
  geom_pointrange(aes(x = X50,
                      xmin = X10,
                      xmax = X90,
                      color = species,
                      alpha = param_type),
                  orientation = "y", size = 1.5, fatten = 1.5) +
  geom_linerange(aes(xmin = X5,
                     xmax = X95,
                     color = species,
                     alpha = param_type),
                 orientation = "y", size = 0.8) +
  scale_alpha_discrete(range = c(.4, 1), name = "Parameter Level", labels = c("Individual", "Species")) +
  scale_color_brewer(palette = "Dark2") +
  theme_bw() +
  theme(legend.key.size = unit(0.75, "cm")) +
  labs(x = "log(Minimum Distance Travelled)",
       y = "Parameter Name",
       title = "Estimated Distance Travelled on First Trial for Species and Individuals")

print(mu_posterior_plot)
```

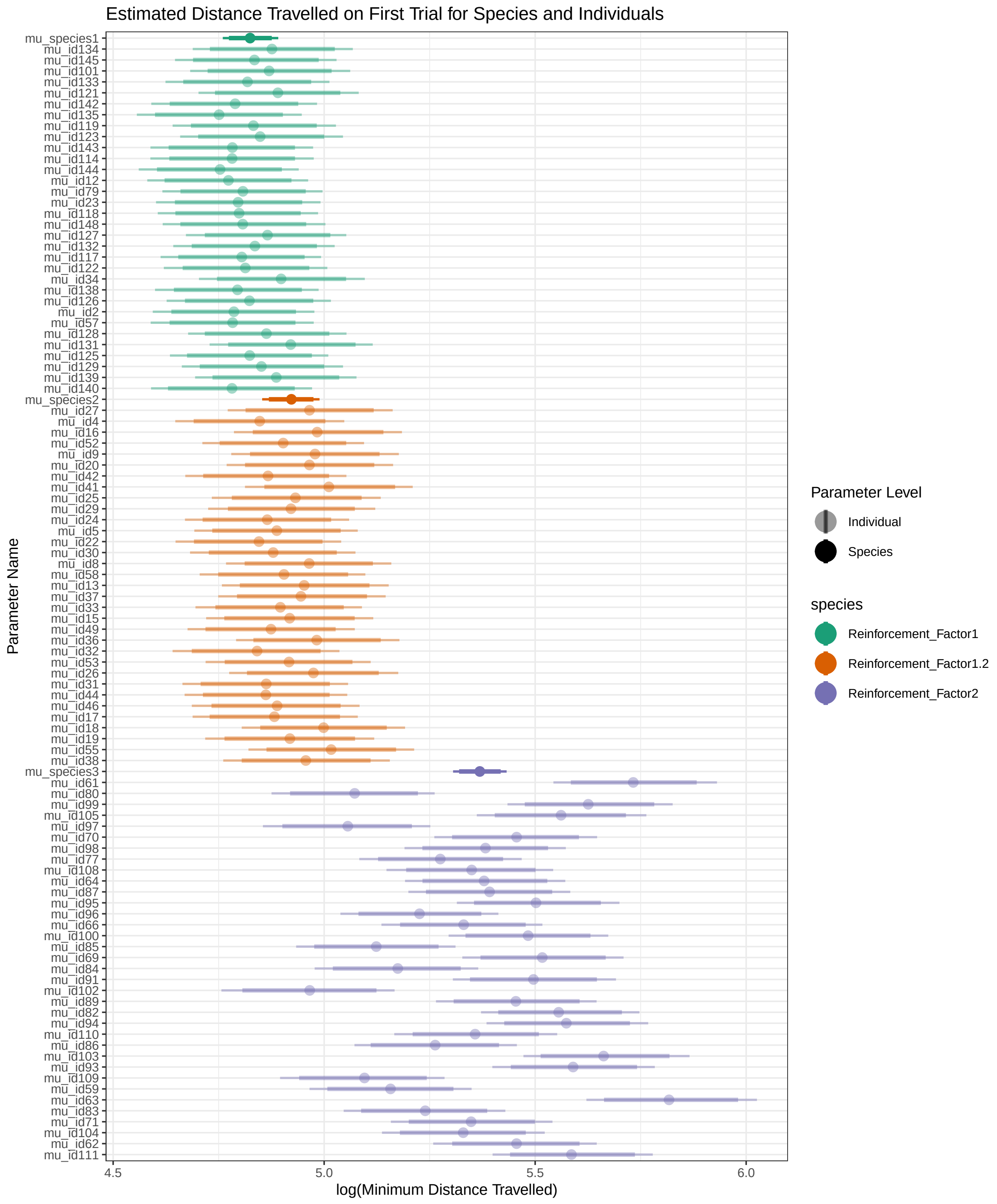


```
#plot slope (b) paramters
b_posterior_plot <- posterior_quantiles_sim_idSubset_DT %>%
  filter(param_type %in% c("b_species", "b_id")) %>%
  arrange(species) %>%
  mutate(parameter = factor(parameter, levels = parameter[length(parameter):1])) %>%
  ggplot(aes(y = parameter)) +
  geom_pointrange(aes(x = X50,
                      xmin = X10,
```

```
                      xmax = X90,
                      alpha = param_type,
                      color = species),
                  orientation = "y", size = 1.5, fatten = 1.5) +
  geom_linerange(aes(xmin = X5,
                     xmax = X95,
                     alpha = param_type,
                     color = species),
                 orientation = "y", size = 0.8) +
  scale_alpha_discrete(range = c(.4, 1), name = "Parameter Level", labels = c("Individual", "Species")) +
  scale_color_brewer(palette = "Dark2") +
  geom_vline(xintercept = 0, color = "red", linetype = "dashed") +
  theme_bw() +
  theme(legend.key.size = unit(0.75, "cm")) +
  labs(x = "log(Distance)/log(Trial)",
       y = "Slope Parameter",
       title = "Estimated Learning Rates for Species and Individuals")

print(b_posterior_plot)
```

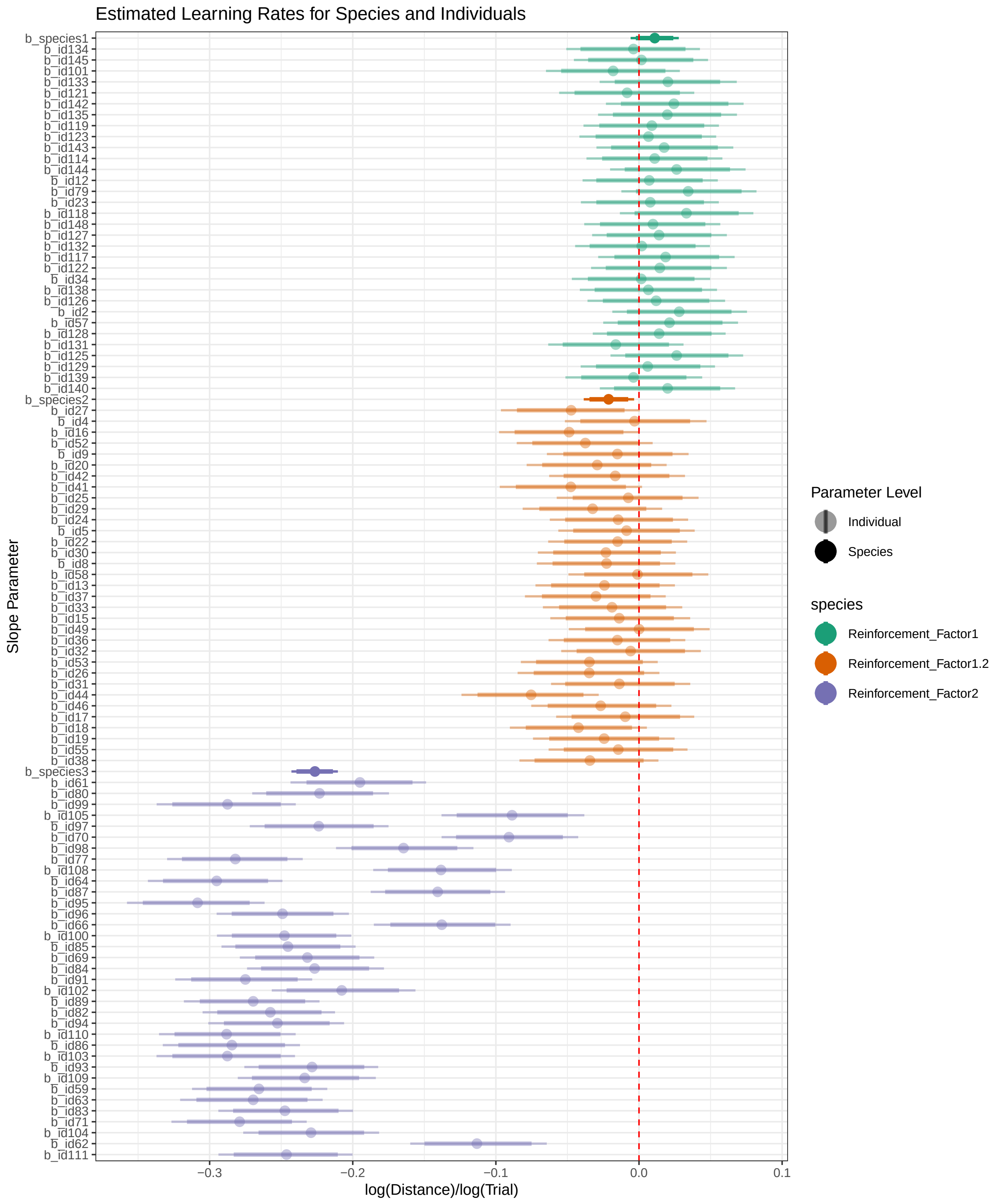

There are several interesting take-aways from these plots. First, there are significant differences in the estimated intercepts for agents of different reinforcement factors. Because we know the behavior of agents on the first trial to be identical, this must reflect the poor fit of the model. Considering the pattern of data, and the structure of the residuals, this is likely caused by a pattern of learning in which shortest route is learned very quickly once found, but the time it takes to find that route is quite stochastic. Real animals, on the other hand, show strategic exploration and gradual improvement - producing the power function learning

curves that fit the empirical data well. This difference in learning pattern for the simulated agents is also apparent in the high individual variation in estimated parameters for the leanring factor 2. The learning rate is less a consequence of how fast the animal learns, but how long it takes to find a shorter route, and which route that is.

None-the-less, we do see overall learning rates that reflect our predictions about the iterative reinforcement algorithm. Though the estimated magnitude of the learning rate for reinforcement factor is small, more than 90% of the posterior estimates for the slope fall below zero, indicated improvment. For Reinforcement Factor 1, on the other hand, 0 is contained within the 80% Credible Interval, giving (as expected) no evidence of improvement. For Reinforcement Factor 2 agents, the magnitude of the slope parameter in the full posterior distribution is unsurprisingly much larger.

```
## pdf
##   2
```

```
## pdf
##   2
```

Finally, we visualize the mean regression models, with a sample of models from the posteriors and the raw data superimposed.

```r
Trial <- 1:max(DT_data_sim$Trial)
pal <- brewer.pal(n = 8, name = "Dark2")

sample_set <- sample(1:4000, size = 100)


for (i in 1:3) { #loop over each species (column in posterior estimates)
  plot(1, type = "n", xlim = c(0, max(DT_data_sim$Trial_scaled)), ylim = c(3.5,7), ylab = NA, xlab = NA, main = levels(DT_data_s
  title(ylab = "log(Distance)", xlab = "log(Trial)", cex.lab = 1, line = 2)
  Distance_mean <- mean(posteriors_sim_DT$mu_species[,i]) + mean(posteriors_sim_DT$b_species[,i]) * log(Trial)
  lines(x = log(Trial), y = Distance_mean, col = "red", lwd = 3)
  for (j in sample_set) {
    lines(x = log(Trial), y = posteriors_sim_DT$mu_species[j,i] + posteriors_sim_DT$b_species[j,i] * log(Trial), col = alpha("re
  }
  points(x = filter(DT_data_sim, Species == levels(DT_data_sim$Species)[i])$Trial_scaled,
         y = filter(DT_data_sim, Species == levels(DT_data_sim$Species)[i])$Distance_scaled,
         pch = 19, cex = 0.5, col = alpha(pal[i], alpha = 0.4))
}
```

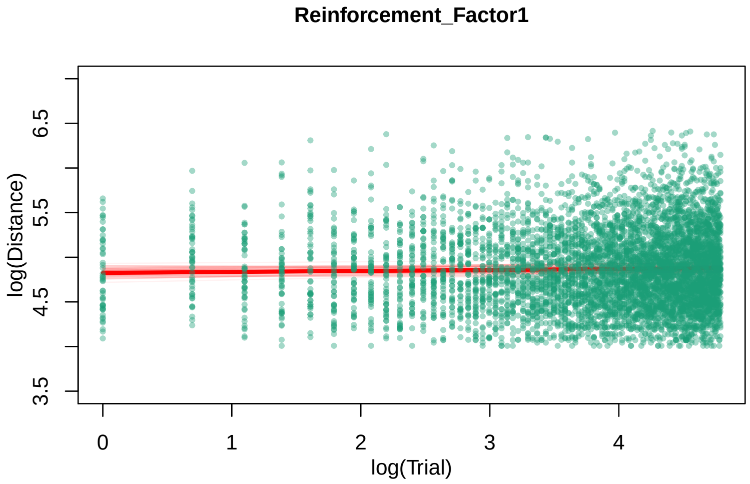

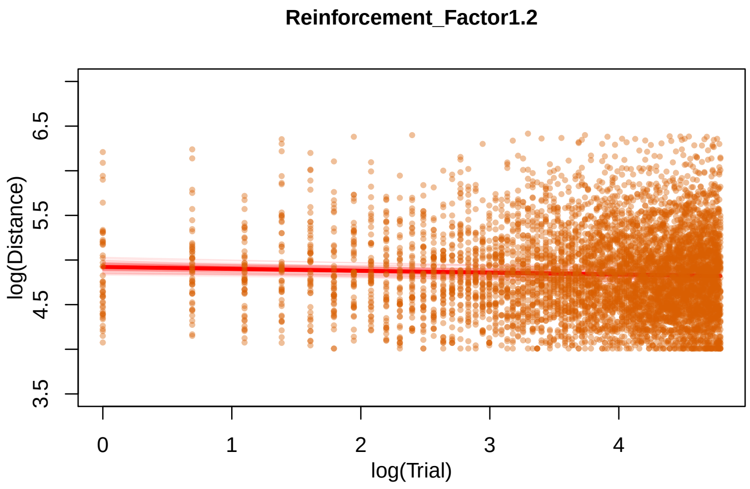

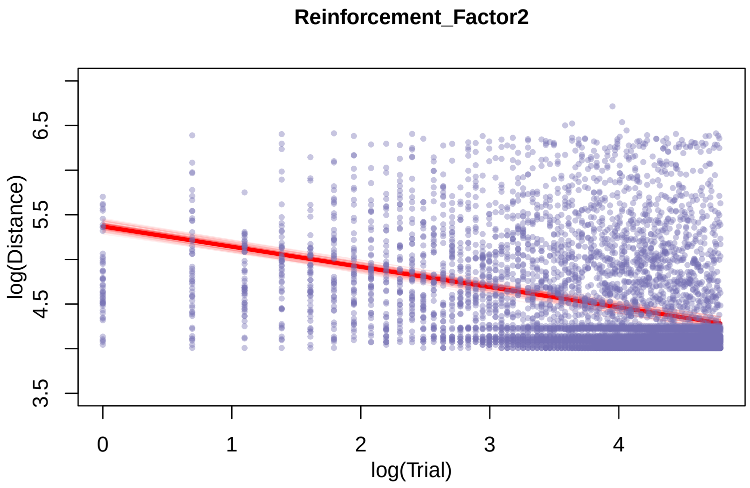

Simulation analysis, Pentagon

We set up the simulated data form the Pentagon array much as we did for the Double Trapezoid

```
#get simulated data from pentagon, remove individuals with fewer than 5 trials, treat Reinforcement Factor as "Species"
Pentagon_data_sim <- data_cleaned %>%
  filter(Species == "None", Array == "Pentagon", max(Trial) >= 5) %>%
  mutate(Species = Source)

Pentagon_data_sim$Species <- factor(Pentagon_data_sim$Species,
                                    levels = c("Reinforcement_Factor1", "Reinforcement_Factor1.2", "Reinforcement_Factor2"))


Pentagon_data_sim <- Pentagon_data_sim %>% filter(as.numeric(ID) %% 2 == 0) #downsample to reduce computational time of statisti

Pentagon_data_sim$ID <- factor(Pentagon_data_sim$ID)

#visualize raw data
```

```r
ggplot(Pentagon_data_sim) + geom_point(aes(x = Trial_scaled, y = Distance_scaled), alpha = 0.4) +
  facet_wrap(~Species)
```

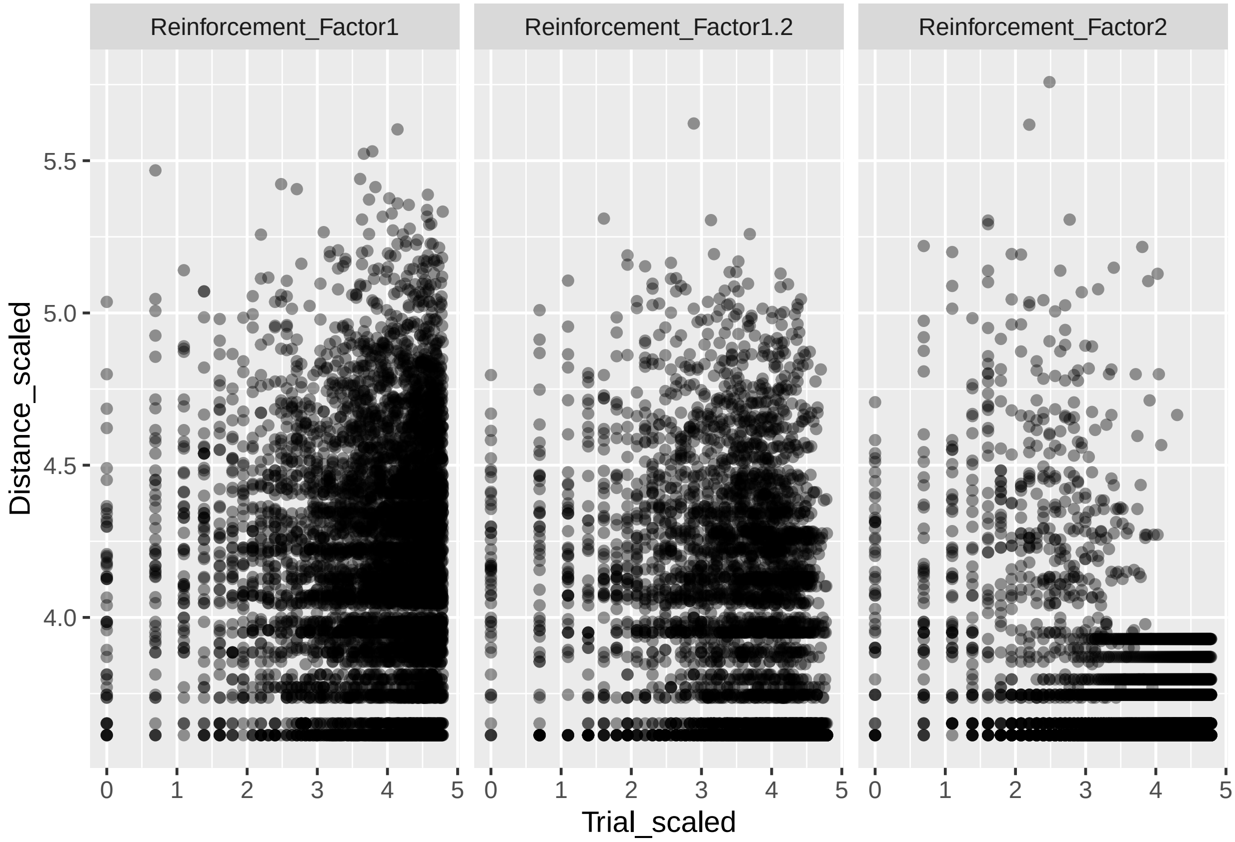


```r
stan_data_sim_Pentagon <- list(N = nrow(Pentagon_data_sim),
                      J = length(unique(Pentagon_data_sim$ID)),
                      K = length(unique(Pentagon_data_sim$Species)),
                      y = Pentagon_data_sim$Distance_scaled,
                      x = Pentagon_data_sim$Trial_scaled,
                      id = as.numeric(factor(Pentagon_data_sim$ID)),
                      sp = as.numeric(Pentagon_data_sim$Species))
```

```r
options(mc.cores = parallel::detectCores())
fit_sim <- stan(file = "../CODE/DT_power_model.stan",
             data = stan_data_sim_Pentagon,
             chains = 4,
             warmup = 2000,
             iter = 4000,
             control = list(max_treedepth = 15)
             )

save(fit_sim, file = "../Results/power_model_fit_Pentagon.Rdata")
```

```r
load("../Results/power_model_fit_Pentagon.Rdata")
```

As with the Double Trapezoid, we check the model diagnositcs after fitting it to the Pentagon simulations

```r
print(fit_sim, pars=c("mu_0", "mu_species", "b_0", "b_species",
                      "sigma_mu_id", "sigma_b_id", "sigma_e", "lp__"),
      probs = c(.1, .5,.9))
```

```
## Inference for Stan model: DT_power_model.
## 4 chains, each with iter=4000; warmup=2000; thin=1;
## post-warmup draws per chain=2000, total post-warmup draws=8000.
##
##                    mean se_mean    sd      10%      50%      90% n_eff Rhat
## mu_0               4.43    0.01  0.49     3.82     4.42     5.06  3740    1
## mu_species[1]     -0.28    0.01  0.49    -0.92    -0.28     0.33  3719    1
## mu_species[2]      0.26    0.01  0.49    -0.38     0.26     0.87  3765    1
## mu_species[3]     -0.32    0.01  0.49    -0.95    -0.31     0.30  3719    1
## b_0               -0.08    0.01  0.50    -0.73    -0.07     0.56  3647    1
## b_species[1]       0.09    0.01  0.50    -0.55     0.08     0.73  3648    1
## b_species[2]      -0.12    0.01  0.50    -0.77    -0.13     0.52  3641    1
## b_species[3]      -0.02    0.01  0.50    -0.67    -0.03     0.62  3649    1
## sigma_mu_id        0.20    0.00  0.01     0.18     0.20     0.22  6191    1
## sigma_b_id         0.04    0.00  0.00     0.04     0.04     0.05  6052    1
## sigma_e[1]         0.83    0.00  0.01     0.82     0.83     0.84 18369    1
## sigma_e[2]         0.68    0.00  0.01     0.67     0.68     0.68 17986    1
## sigma_e[3]         0.30    0.00  0.00     0.29     0.30     0.30 17675    1
## lp__           16198.06    0.25 13.22 16181.34 16198.34 16214.79  2889    1
##
## Samples were drawn using NUTS(diag_e) at Tue Oct 05 10:07:41 2021.
## For each parameter, n_eff is a crude measure of effective sample size,
## and Rhat is the potential scale reduction factor on split chains (at
## convergence, Rhat=1).
```

```r
traceplot(fit_sim, pars = c("mu_0", "mu_species", "b_0", "b_species"), inc_warmup = TRUE)
pairs(fit_sim, pars = c("mu_0", "mu_species"), las = 1)
pairs(fit_sim, pars = c("b_0", "b_species"), las = 1)

#residual analysis
r_bar <- apply(extract(fit_sim, "pearson_residual")[[1]], MARGIN = 2, mean)
y_pred <- apply(extract(fit_sim, "y_pred")[[1]], MARGIN = 2, mean)
plot(x = y_pred, y = r_bar) #residuals against prediction
plot(x = stan_data_sim_Pentagon$x, y = r_bar)
plot(x = stan_data_sim_Pentagon$sp, y = r_bar)
qqnorm(r_bar)
```

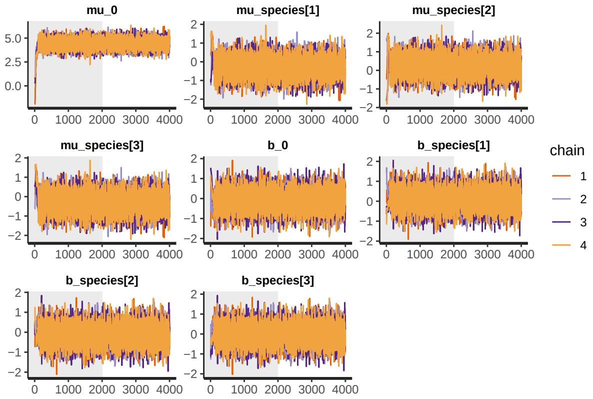
mu_0
mu_species[1]
mu_species[2]
mu_species[3]
b_0
b_species[1]
b_species[2]
b_species[3]
chain
1
2
3
4

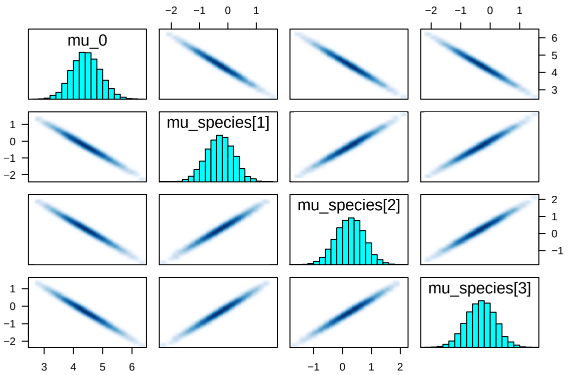
mu_0
mu_species[1]
mu_species[2]
mu_species[3]

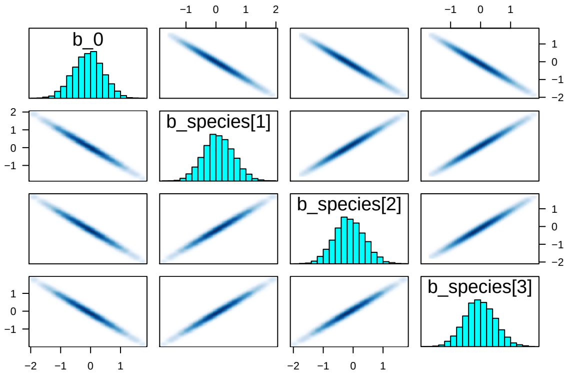
b_0
b_species[1]
b_species[2]
b_species[3]

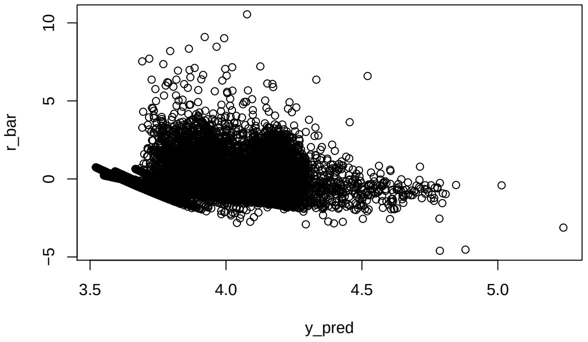
r_bar
y_pred

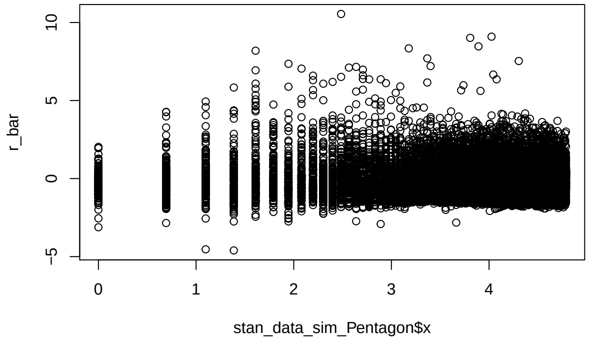
r_bar
stan_data_sim_Pentagon$x

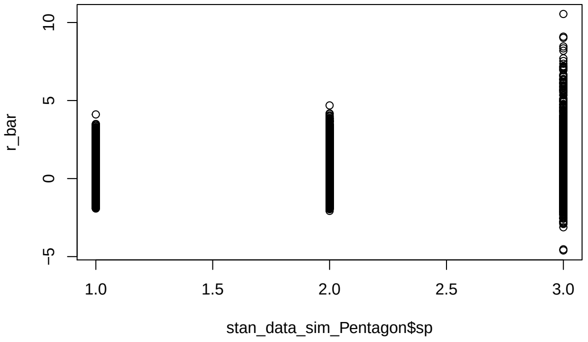
r_bar
stan_data_sim_Pentagon$sp

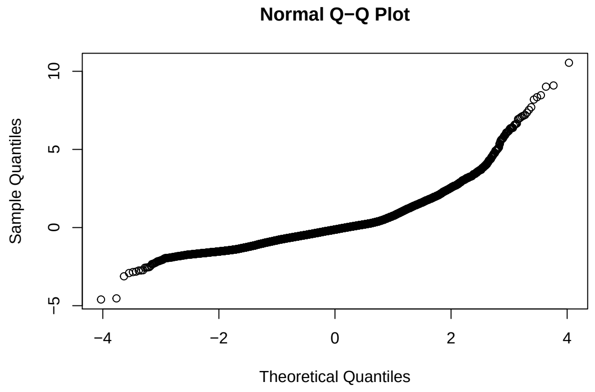

These results look much as they did for the double trapezoid: an identifiable, well-mixed model that fails to capture the real structure of the data, giving residuals that are biased by the Reinforcement Factor of the agent and the trial number.

Having assessed the fit of the model, we look at the parameter estimates, first getting species and individual level estimates by adding relevant deviations to mean estimates.

```
posteriors_sim_Pentagon <- extract(fit_sim)

#find the estimated intercepts by species for each sample - used for plotting estimated learning curves
posteriors_sim_Pentagon$mu_species <- apply(posteriors_sim_Pentagon[["mu_species"]],
                                   MARGIN = 2,
                                   FUN = function(X) X + posteriors_sim_Pentagon[["mu_0"]])

posteriors_sim_Pentagon$b_species <- apply(posteriors_sim_Pentagon[["b_species"]],
                                  MARGIN = 2,
                                  FUN = function(X) X + posteriors_sim_Pentagon[["b_0"]])

#do the same for individual estimates
for(i in 1:ncol(posteriors_sim_Pentagon[["mu_id"]])){
  #get species of id factor level i
  sp <- Pentagon_data_sim$Species[match(levels(Pentagon_data_sim$ID),
                                        Pentagon_data_sim$ID)][i]
  #add individual deviation to species level intercept
  posteriors_sim_Pentagon[["mu_id"]][,i] <- posteriors_sim_Pentagon[["mu_species"]][,sp] + posteriors_s
  #add individual deviation to species level slope
  posteriors_sim_Pentagon[["b_id"]][,i] <- posteriors_sim_Pentagon[["b_species"]][,sp] + posteriors_sim
}
```

To visualize the resulting posteriors, we determine quantiles of each posterior distribution and plot the 80% (thick bars) and 90% (thin bars) confidence intervals for each species and individual level parameter.

```
#function to get quantiles from each column of a matrix (corresponding to the posterior distribution of a variable)
apply_quant <- function(x) apply(as.matrix(x), MARGIN = 2, quantile, c(0.025, .05, .10, .5, .90, .95, 0.975))

#get quantiles for the first 8 parameters in "posteriors" list
posteriors_sim_Pentagon[1:8] %>% lapply(FUN = apply_quant) %>%
  #bind list of quanitile vectors into a matrix
  do.call(args = ., what = cbind) %>%
  #transpose and save as dataframe
  t() %>% as.data.frame() -> posterior_quantiles_sim_Pentagon

#remove % and start all names with a character to avoid trouble in ggplot
colnames(posterior_quantiles_sim_Pentagon) <- paste("X", gsub("%", "", colnames(posterior_quantiles_sim_Pentagon)), sep = "")
```

```r
#add metadata to describe each parameter with quantiles
posterior_quantiles_sim_Pentagon$parameter <-
  #get a vector named with parameter names for each type of parameter, then extract and concatenate those names.
  names(unlist(sapply(posteriors_sim_Pentagon[1:8], function(X) seq(to = ncol(as.matrix(X))))))
posterior_quantiles_sim_Pentagon$param_type <- rep(names(posteriors_sim_Pentagon[1:8]),
                                          times = sapply(posteriors_sim_Pentagon[1:8],
                                                         function(X) ncol(as.matrix(X))))

#~~give species metadata for parameters where relevant
posterior_quantiles_sim_Pentagon$species <- NA
posterior_quantiles_sim_Pentagon$species[posterior_quantiles_sim_Pentagon$param_type == "mu_id"] <-
  as.character(Pentagon_data_sim$Species[match(levels(Pentagon_data_sim$ID), Pentagon_data_sim$ID)])
posterior_quantiles_sim_Pentagon$species[posterior_quantiles_sim_Pentagon$param_type == "b_id"] <-
  as.character(Pentagon_data_sim$Species[match(levels(Pentagon_data_sim$ID), Pentagon_data_sim$ID)])
posterior_quantiles_sim_Pentagon$species[posterior_quantiles_sim_Pentagon$param_type == "mu_species"] <-
  levels(Pentagon_data_sim$Species)
posterior_quantiles_sim_Pentagon$species[posterior_quantiles_sim_Pentagon$param_type == "b_species"] <-
  levels(Pentagon_data_sim$Species)
#~~~~~~~~~~

posterior_quantiles_sim_Pentagon_mu_id <- filter(posterior_quantiles_sim_Pentagon, param_type == "mu_id")
posterior_quantiles_sim_Pentagon_b_id <- filter(posterior_quantiles_sim_Pentagon, param_type == "b_id")

#get a sample of 100 individuals to visualize individual level variance
sample_set <- sample(x = 1:nrow(posterior_quantiles_sim_Pentagon_mu_id), size = 100)

#get only id level quantiles
posterior_quantiles_sim_Pentagon_idSubset <- rbind(filter(posterior_quantiles_sim_Pentagon, ! param_type  %in% c("mu_id", "b_id"
                                          posterior_quantiles_sim_Pentagon_mu_id[sample_set,],
                                          posterior_quantiles_sim_Pentagon_b_id[sample_set,]
                                          )
```

```r
#plot intercept(mu) parameters
mu_posterior_plot <- posterior_quantiles_sim_Pentagon_idSubset %>%
  filter(param_type %in% c("mu_species", "mu_id")) %>%
  arrange(species) %>%
  mutate(parameter = factor(parameter, levels = parameter[length(parameter):1])) %>%
  ggplot(aes(y = parameter)) +
  geom_pointrange(aes(x = X50,
                      xmin = X10,
                      xmax = X90,
                      color = species,
                      alpha = param_type),
                  orientation = "y", size = 1.5, fatten = 1.5) +
  geom_linerange(aes(xmin = X5,
                     xmax = X95,
                     color = species,
                     alpha = param_type),
                 orientation = "y", size = 0.8) +
  scale_alpha_discrete(range = c(.4, 1), name = "Parameter Level", labels = c("Individual", "Species")) +
  scale_color_brewer(palette = "Dark2") +
  theme_bw() +
  theme(legend.key.size = unit(0.75, "cm")) +
  labs(x = "log(Minimum Distance Travelled)",
       y = "Parameter Name",
       title = "Estimated Distance Travelled on First Trial for Species and Individuals")

print(mu_posterior_plot)
```

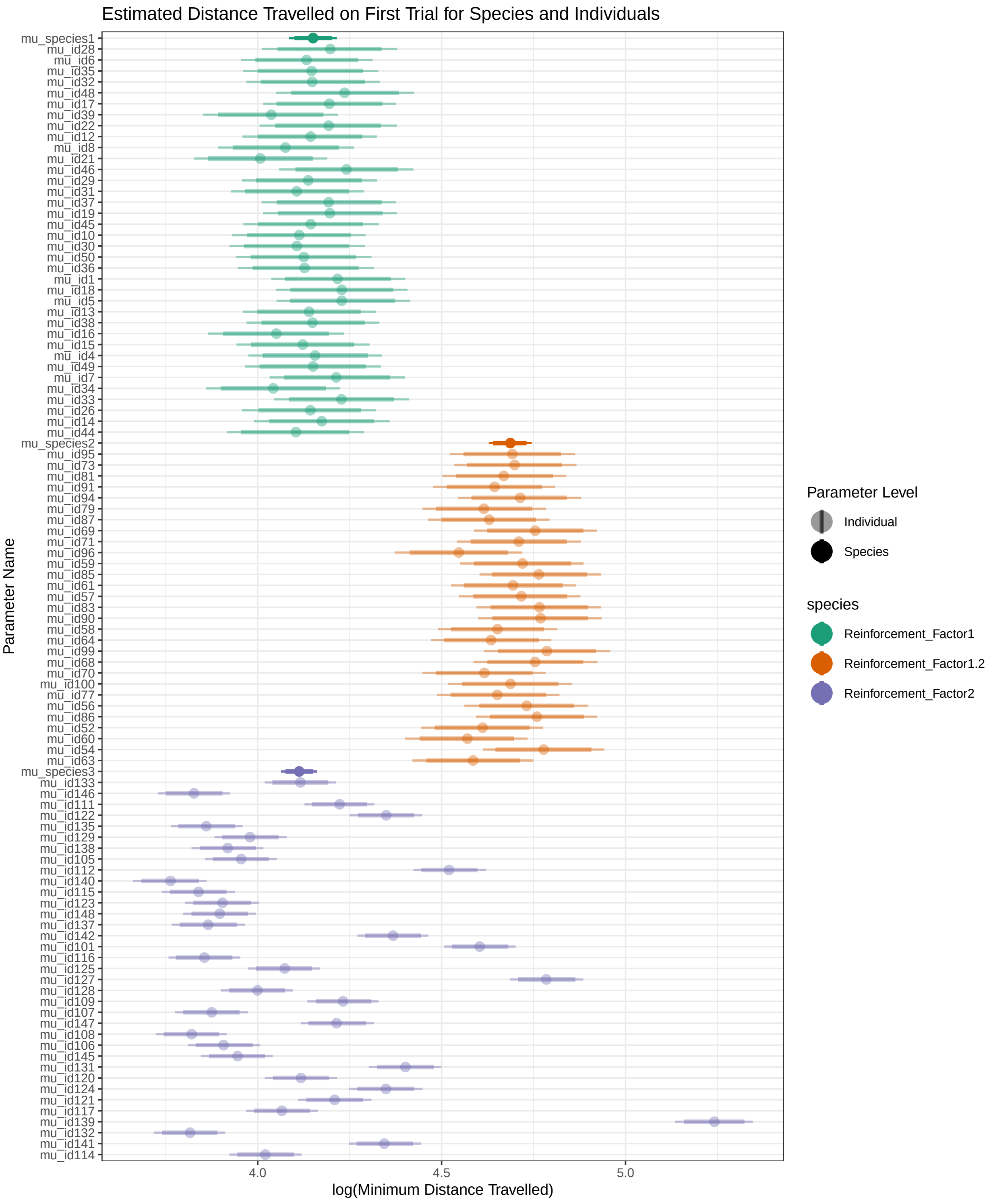


```r
#plot slope (b) paramters
b_posterior_plot <- posterior_quantiles_sim_Pentagon_idSubset %>%
  filter(param_type %in% c("b_species", "b_id")) %>%
  arrange(species) %>%
  mutate(parameter = factor(parameter, levels = parameter[length(parameter):1])) %>%
  ggplot(aes(y = parameter)) +
  geom_pointrange(aes(x = X50,
                      xmin = X10,
```

```
                      xmax = X90,
                      alpha = param_type,
                      color = species),
                  orientation = "y", size = 1.5, fatten = 1.5) +
  geom_linerange(aes(xmin = X5,
                     xmax = X95,
                     alpha = param_type,
                     color = species),
                 orientation = "y", size = 0.8) +
  scale_alpha_discrete(range = c(.4, 1), name = "Parameter Level", labels = c("Individual", "Species")) +
  scale_color_brewer(palette = "Dark2") +
  geom_vline(xintercept = 0, color = "red", linetype = "dashed") +
  theme_bw() +
  theme(legend.key.size = unit(0.75, "cm")) +
  labs(x = "log(Distance)/log(Trial)",
       y = "Slope Parameter",
       title = "Estimated Learning Rates for Species and Individuals")

print(b_posterior_plot)
```

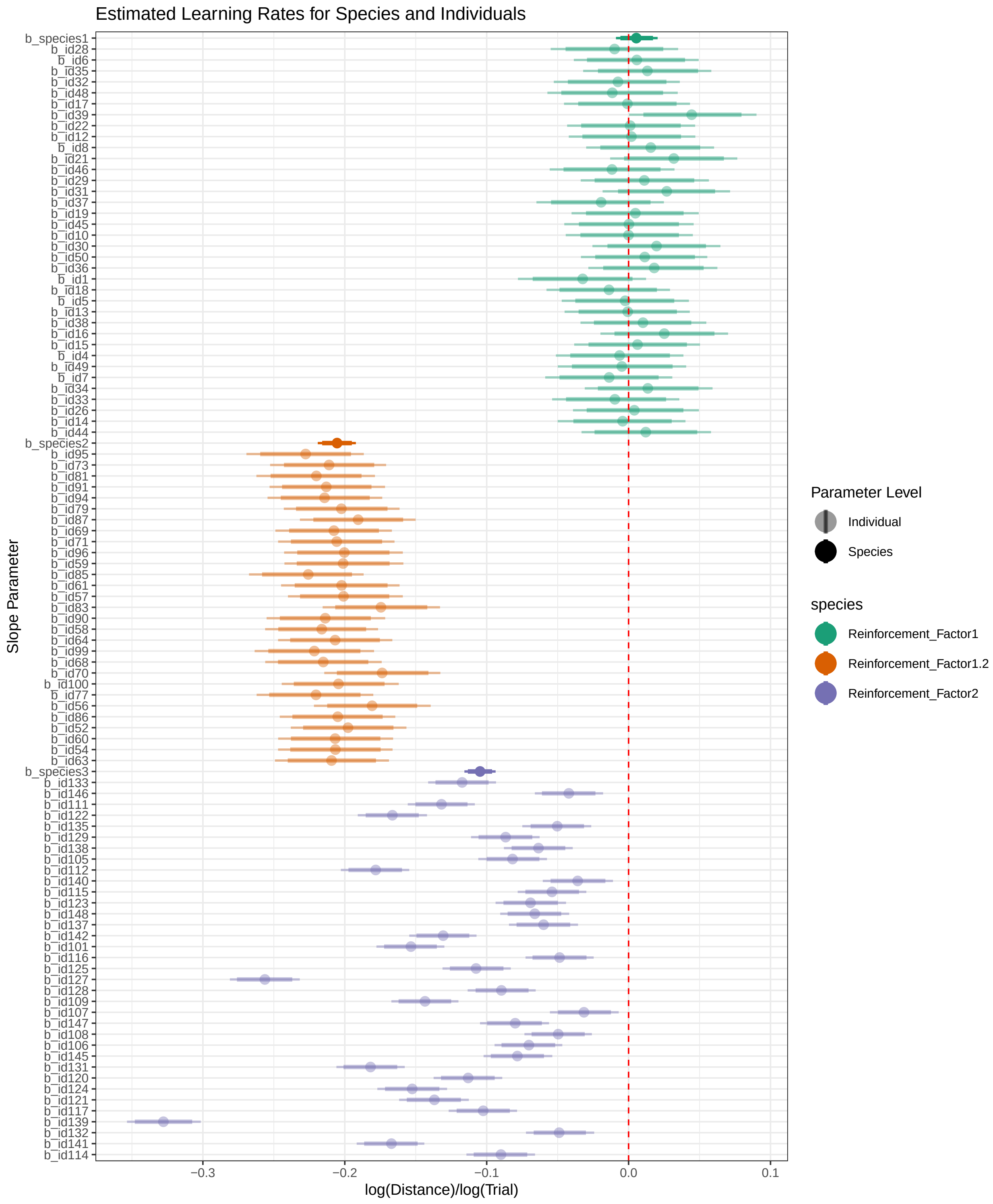
Estimated Learning Rates for Species and Individuals
Slope Parameter
b_species1
b_id28
b_id6
b_id35
b_id32
b_id48
b_id17
b_id39
b_id22
b_id12
b_id8
b_id21
b_id46
b_id29
b_id31
b_id37
b_id19
b_id45
b_id10
b_id30
b_id50
b_id36
b_id1
b_id18
b_id5
b_id13
b_id38
b_id16
b_id15
b_id4
b_id49
b_id7
b_id34
b_id33
b_id26
b_id14
b_id44
b_species2
b_id95
b_id73
b_id81
b_id91
b_id94
b_id79
b_id87
b_id69
b_id71
b_id96
b_id59
b_id85
b_id61
b_id57
b_id83
b_id90
b_id58
b_id64
b_id99
b_id68
b_id70
b_id100
b_id77
b_id56
b_id86
b_id52
b_id60
b_id54
b_id63
b_species3
b_id133
b_id146
b_id111
b_id122
b_id135
b_id129
b_id138
b_id105
b_id112
b_id140
b_id115
b_id123
b_id148
b_id137
b_id142
b_id101
b_id116
b_id125
b_id127
b_id128
b_id109
b_id107
b_id147
b_id108
b_id106
b_id145
b_id131
b_id120
b_id124
b_id121
b_id117
b_id139
b_id132
b_id141
b_id114
−0.3
−0.2
−0.1
0.0
0.1
log(Distance)/log(Trial)
Parameter Level
Individual
Species
species
Reinforcement_Factor1
Reinforcement_Factor1.2
Reinforcement_Factor2

```
## pdf
##   2

## pdf
##   2
```

The estimated learning rate for reinforcement factor 1.2 is greater in magnitude than for reinforcement factor 2! Some data visualization is necessary to understand what is going on.

```
Trial <- 1:max(Pentagon_data_sim$Trial)
pal <- brewer.pal(n = 8, name = "Dark2")

sample_set <- sample(1:4000, size = 100)


for (i in 1:3) { #loop over each species (column in posterior estimates)
  plot(1, type = "n", xlim = c(0, max(Pentagon_data_sim$Trial_scaled)), ylim = c(3.5,6), ylab = NA, xlab = NA, main = levels(Pen
  title(ylab = "log(Distance)", xlab = "log(Trial)", cex.lab = 1, line = 2)
  Distance_mean <- mean(posteriors_sim_Pentagon$mu_species[,i]) + mean(posteriors_sim_Pentagon$b_species[,i]) * log(Trial)
  lines(x = log(Trial), y = Distance_mean, col = "red", lwd = 3)
  for (j in sample_set) {
    lines(x = log(Trial), y = posteriors_sim_Pentagon$mu_species[j,i] + posteriors_sim_Pentagon$b_species[j,i] * log(Trial), col
  }
  points(x = filter(Pentagon_data_sim, Species == levels(Pentagon_data_sim$Species)[i])$Trial_scaled,
         y = filter(Pentagon_data_sim, Species == levels(Pentagon_data_sim$Species)[i])$Distance_scaled,
         pch = 19, cex = 0.5, col = alpha(pal[i], alpha = 0.4))
}
```

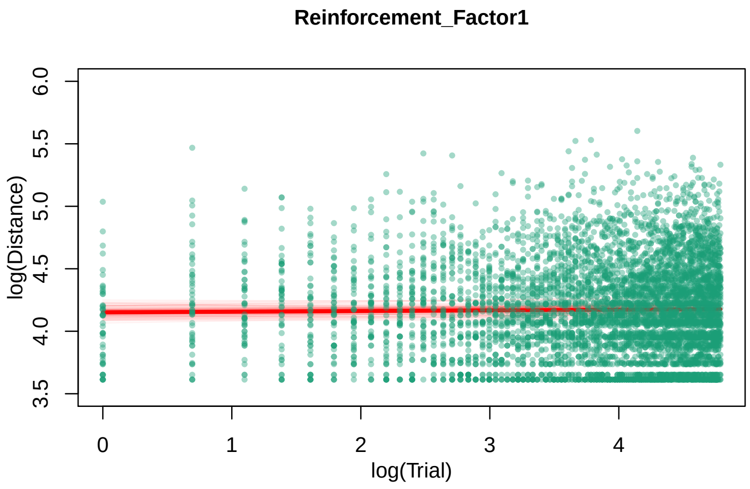

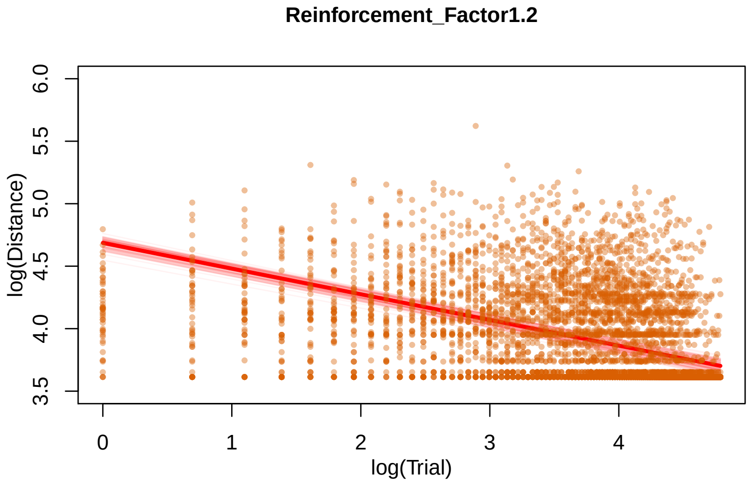

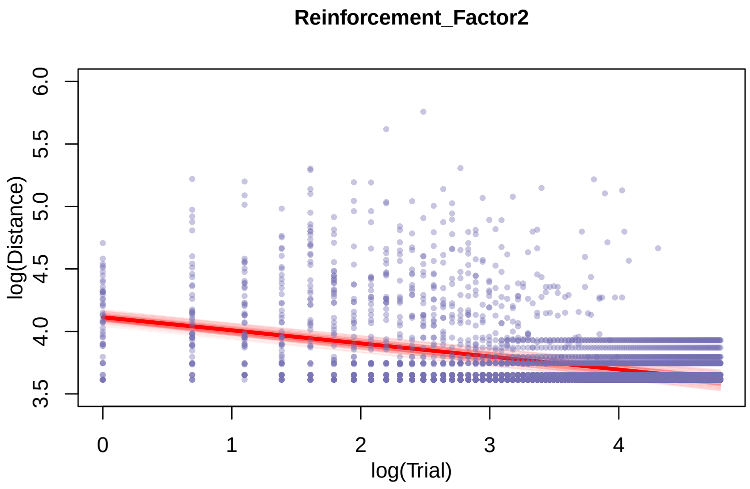

Simulation analysis, Z-Array

We set up the simulated data form the Pentagon array much as we did for the Double Trapezoid

```
#get simulated data from pentagon, remove individuals with fewer than 5 trials, treat Reinforcement Factor as "Species"
Zed_data_sim <- data_cleaned %>%
  filter(Species == "None", Array == "Zarray", max(Trial) >= 5) %>%
  mutate(Species = Source)

Zed_data_sim$Species <- factor(Zed_data_sim$Species,
                                     levels = c("Reinforcement_Factor1", "Reinforcement_Factor1.2", "Reinforcement_Factor2"))


Zed_data_sim <- Zed_data_sim %>% filter(as.numeric(ID) %% 2 == 0) #downsample to reduce computational time of statistical model

Zed_data_sim$ID <- factor(Zed_data_sim$ID)

#visualize raw data
ggplot(Zed_data_sim) + geom_point(aes(x = Trial_scaled, y = Distance_scaled), alpha = 0.4) +
  facet_wrap(~Species)
```

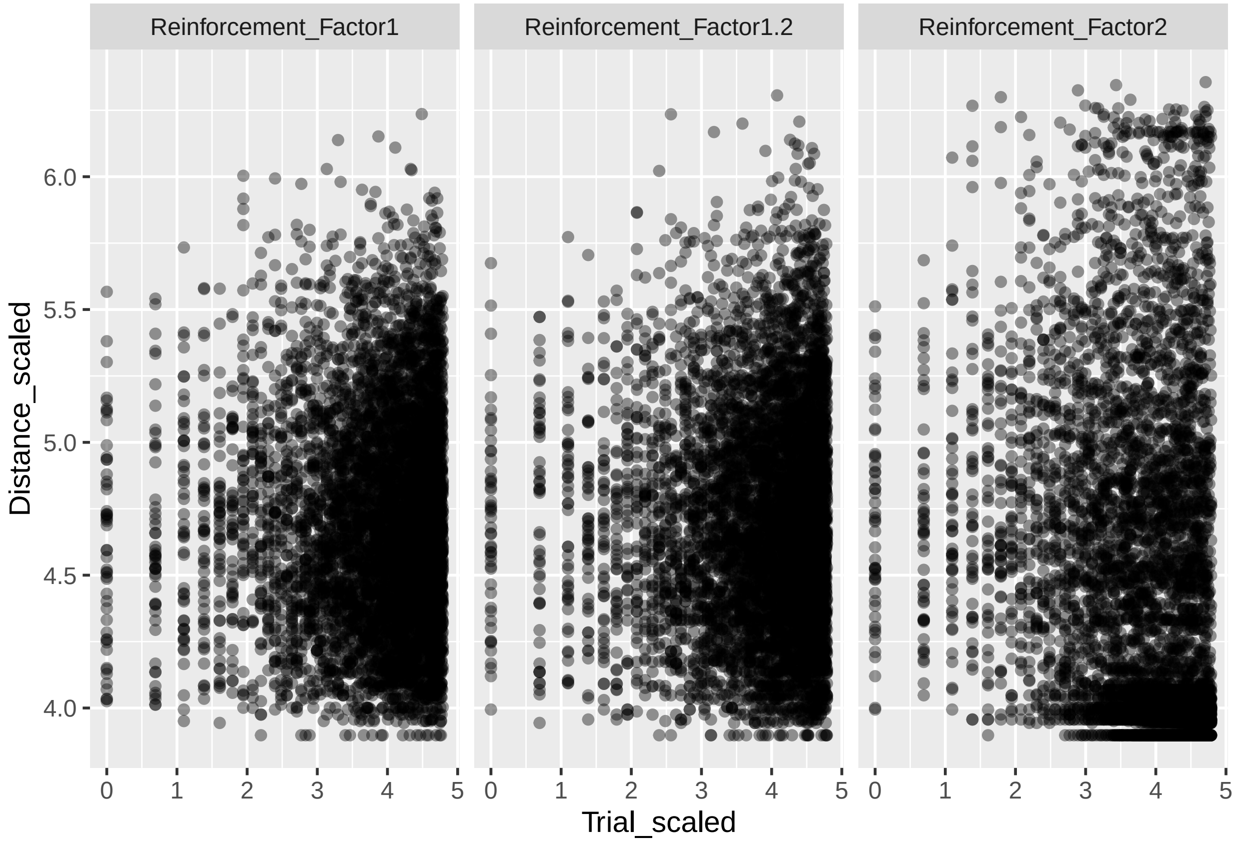


```
stan_data_sim_Zed <- list(N = nrow(Zed_data_sim),
                          J = length(unique(Zed_data_sim$ID)),
                          K = length(unique(Zed_data_sim$Species)),
                          y = Zed_data_sim$Distance_scaled,
                          x = Zed_data_sim$Trial_scaled,
                          id = as.numeric(factor(Zed_data_sim$ID)),
                          sp = as.numeric(Zed_data_sim$Species))
```

```
options(mc.cores = parallel::detectCores())
fit_sim <- stan(file = "../CODE/DT_power_model.stan",
             data = stan_data_sim_Zed,
             chains = 4,
             warmup = 1000,
             iter = 2000,
             control = list(max_treedepth = 15)
             )

save(fit_sim, file = "../Results/power_model_fit_Zed.Rdata")
```

```
load("../Results/power_model_fit_Zed.Rdata")
```

As with the Double Trapezoid, we check the model diagnostics after fitting it to the Zed simulations

```
print(fit_sim, pars=c("mu_0", "mu_species", "b_0", "b_species",
                      "sigma_mu_id", "sigma_b_id", "sigma_e", "lp__"),
      probs = c(.1, .5,.9))
```

```
## Inference for Stan model: DT_power_model.
## 4 chains, each with iter=2000; warmup=1000; thin=1;
## post-warmup draws per chain=1000, total post-warmup draws=4000.
##
##                 mean se_mean    sd     10%     50%     90% n_eff Rhat
## mu_0            4.87    0.01  0.51    4.20    4.88    5.50  3028 1.00
## mu_species[1]  -0.15    0.01  0.51   -0.79   -0.16    0.52  3018 1.00
## mu_species[2]  -0.14    0.01  0.51   -0.76   -0.15    0.53  3025 1.00
## mu_species[3]   0.38    0.01  0.51   -0.26    0.38    1.05  3038 1.00
## b_0            -0.06    0.01  0.51   -0.70   -0.06    0.58  2660 1.00
## b_species[1]    0.05    0.01  0.51   -0.59    0.06    0.70  2665 1.00
## b_species[2]    0.05    0.01  0.51   -0.59    0.05    0.70  2658 1.00
## b_species[3]   -0.15    0.01  0.51   -0.79   -0.16    0.49  2653 1.00
## sigma_mu_id     0.11    0.00  0.02    0.08    0.11    0.14   255 1.02
## sigma_b_id      0.05    0.00  0.00    0.05    0.05    0.06   937 1.01
## sigma_e[1]      0.89    0.00  0.01    0.88    0.89    0.90  9967 1.00
## sigma_e[2]      0.92    0.00  0.01    0.91    0.92    0.93  8928 1.00
## sigma_e[3]      0.95    0.00  0.01    0.94    0.95    0.96  4468 1.00
## lp__         6975.35    1.92 28.95 6942.57 6972.95 7010.55   228 1.03
##
## Samples were drawn using NUTS(diag_e) at Mon Oct 11 05:23:07 2021.
## For each parameter, n_eff is a crude measure of effective sample size,
## and Rhat is the potential scale reduction factor on split chains (at
## convergence, Rhat=1).
```

```
traceplot(fit_sim, pars = c("mu_0", "mu_species", "b_0", "b_species"), inc_warmup = TRUE)
pairs(fit_sim, pars = c("mu_0", "mu_species"), las = 1)
pairs(fit_sim, pars = c("b_0", "b_species"), las = 1)

#residual analysis
r_bar <- apply(extract(fit_sim, "pearson_residual")[[1]], MARGIN = 2, mean)
y_pred <- apply(extract(fit_sim, "y_pred")[[1]], MARGIN = 2, mean)
plot(x = y_pred, y = r_bar) #residuals against prediction
plot(x = stan_data_sim_Zed$x, y = r_bar)
plot(x = stan_data_sim_Zed$sp, y = r_bar)
qqnorm(r_bar)
```

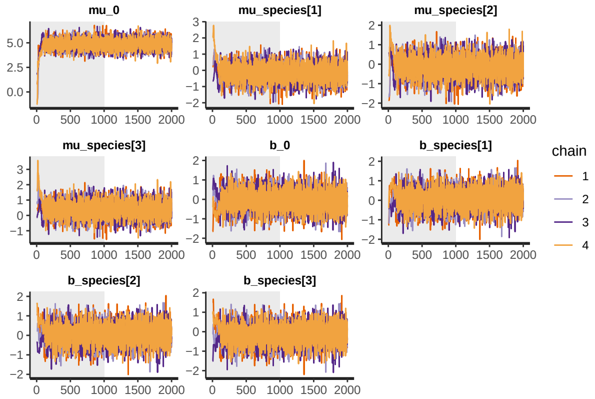

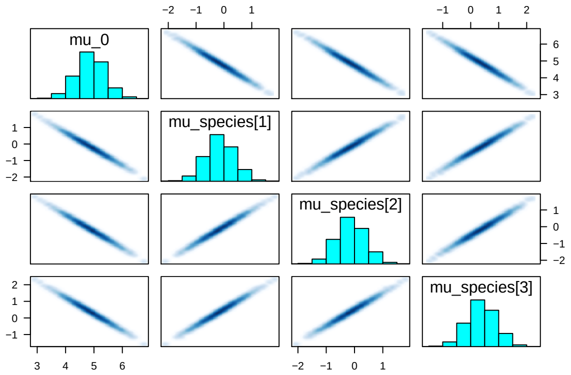

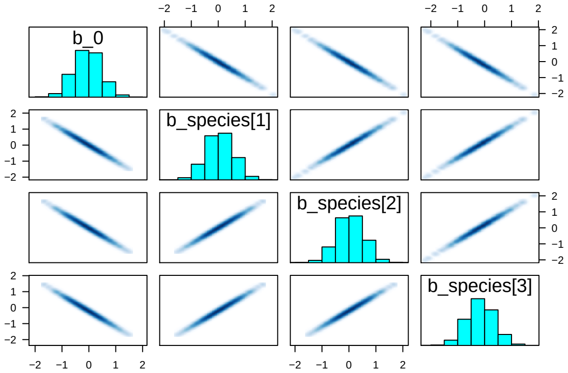

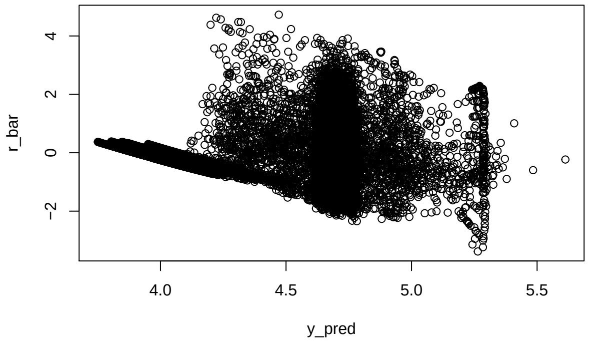

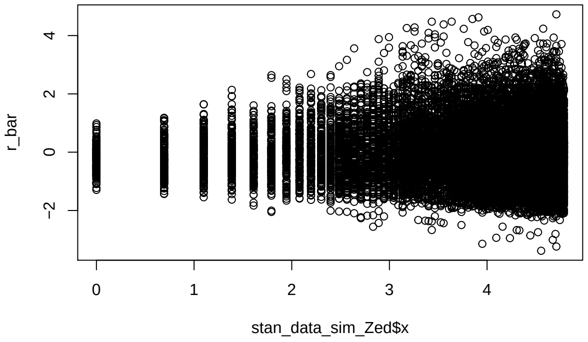

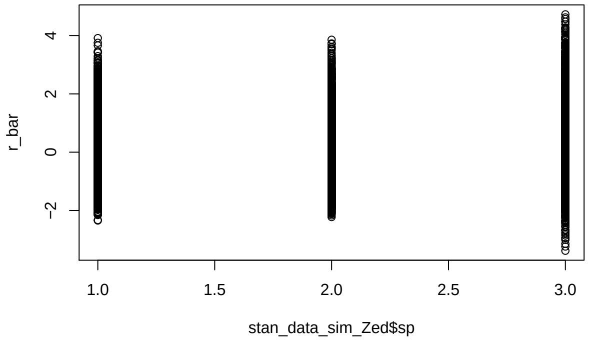

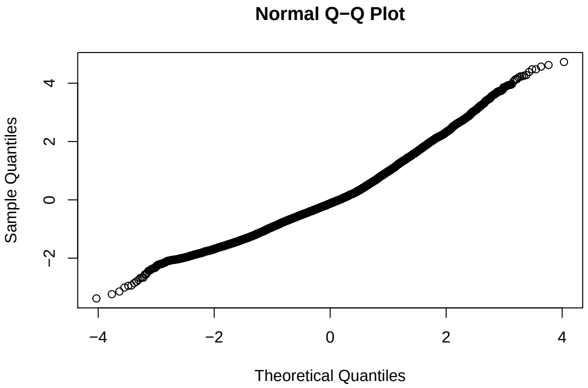

THe MCMC chains for this model did not mix as well as in the DT or Pentagon simulations, however we deem the Rhat values and effective sample sizes to indicate good enough mixture and convergence to continue the analysis. As with the DT and Pentagon simulations, the learning model fails to capture some of the structure in the data, with residuals showing a downward trend along predicted distances travelled. Overall, though, residuals from the ZED array appear to be the most independantly and identically distributed relative to the other simulation analyses.

Having assessed the fit of the model, we look at the parameter estimates, first getting species and individual level estimates by adding relevant deviations to mean estimates.

```
posteriors_sim_Zed <- extract(fit_sim)

#find the estimated intercepts by species for each sample - used for plotting estimated learning curves
posteriors_sim_Zed$mu_species <- apply(posteriors_sim_Zed[["mu_species"]],
```

```
                                             MARGIN = 2,
                                             FUN = function(X) X + posteriors_sim_Zed[["mu_0"]])

posteriors_sim_Zed$b_species <- apply(posteriors_sim_Zed[["b_species"]],
                                      MARGIN = 2,
                                      FUN = function(X) X + posteriors_sim_Zed[["b_0"]])

#do the same for individual estimates
for(i in 1:ncol(posteriors_sim_Zed[["mu_id"]])){
  #get species of id factor level i
  sp <- Zed_data_sim$Species[match(levels(Zed_data_sim$ID),
                                  Zed_data_sim$ID)][i]
  #add individual deviation to species level intercept
  posteriors_sim_Zed[["mu_id"]][,i] <- posteriors_sim_Zed[["mu_species"]][,sp] + posteriors_sim_Zed[["mu
  #add individual deviation to species level slope
  posteriors_sim_Zed[["b_id"]][,i] <- posteriors_sim_Zed[["b_species"]][,sp] + posteriors_sim_Zed[["b_i
}
```

To visualize the resulting posteriors, we determine quantiles of each posterior distribution and plot the 80% (thick bars) and 90% (thin bars) confidence intervals for each species and individual level parameter.

```
#function to get quantiles from each column of a matrix (corresponding to the posterior distribution of a variable)
apply_quant <- function(x) apply(as.matrix(x), MARGIN = 2, quantile, c(0.025, .05, .10, .5, .90, .95, 0.975))

#get quantiles for the first 8 parameters in "posteriors" list
posteriors_sim_Zed[1:8] %>% lapply(FUN = apply_quant) %>%
  #bind list of quanitile vectors into a matrix
  do.call(args = ., what = cbind) %>%
  #transpose and save as dataframe
  t() %>% as.data.frame() -> posterior_quantiles_sim_Zed

#remove % and start all names with a character to avoid trouble in ggplot
colnames(posterior_quantiles_sim_Zed) <- paste("X", gsub("%", "", colnames(posterior_quantiles_sim_Zed)), sep = "")

#add metadata to describe each parameter with quantiles
posterior_quantiles_sim_Zed$parameter <-
  #get a vector named with parameter names for each type of parameter, then extract and concatenate those names.
  names(unlist(sapply(posteriors_sim_Zed[1:8], function(X) seq(to = ncol(as.matrix(X))))))
posterior_quantiles_sim_Zed$param_type <- rep(names(posteriors_sim_Zed[1:8]),
                                          times = sapply(posteriors_sim_Zed[1:8],
                                                         function(X) ncol(as.matrix(X))))

#~~give species metadata for parameters where relevant
posterior_quantiles_sim_Zed$species <- NA
posterior_quantiles_sim_Zed$species[posterior_quantiles_sim_Zed$param_type == "mu_id"] <-
  as.character(Zed_data_sim$Species[match(levels(Zed_data_sim$ID), Zed_data_sim$ID)])
posterior_quantiles_sim_Zed$species[posterior_quantiles_sim_Zed$param_type == "b_id"] <-
  as.character(Zed_data_sim$Species[match(levels(Zed_data_sim$ID), Zed_data_sim$ID)])
posterior_quantiles_sim_Zed$species[posterior_quantiles_sim_Zed$param_type == "mu_species"] <-
  levels(Zed_data_sim$Species)
posterior_quantiles_sim_Zed$species[posterior_quantiles_sim_Zed$param_type == "b_species"] <-
  levels(Zed_data_sim$Species)
#~~~~~~~~~~

posterior_quantiles_sim_Zed_mu_id <- filter(posterior_quantiles_sim_Zed, param_type == "mu_id")
posterior_quantiles_sim_Zed_b_id <- filter(posterior_quantiles_sim_Zed, param_type == "b_id")

#get a sample of 100 individuals to visualize individual level variance
sample_set <- sample(x = 1:nrow(posterior_quantiles_sim_Zed_mu_id), size = 100)

#get only id level quantiles
posterior_quantiles_sim_Zed_idSubset <- rbind(filter(posterior_quantiles_sim_Zed, ! param_type  %in% c("mu_id", "b_id")),
```

```
                                            posterior_quantiles_sim_Zed_mu_id[sample_set,],
                                            posterior_quantiles_sim_Zed_b_id[sample_set,]
                                            )
```

```
#plot intercept(mu) parameters
mu_posterior_plot <- posterior_quantiles_sim_Zed_idSubset %>%
  filter(param_type %in% c("mu_species", "mu_id")) %>%
  arrange(species) %>%
  mutate(parameter = factor(parameter, levels = parameter[length(parameter):1])) %>%
  ggplot(aes(y = parameter)) +
  geom_pointrange(aes(x = X50,
                      xmin = X10,
                      xmax = X90,
                      color = species,
                      alpha = param_type),
                  orientation = "y", size = 1.5, fatten = 1.5) +
  geom_linerange(aes(xmin = X5,
                     xmax = X95,
                     color = species,
                     alpha = param_type),
                 orientation = "y", size = 0.8) +
  scale_alpha_discrete(range = c(.4, 1), name = "Parameter Level", labels = c("Individual", "Species")) +
  scale_color_brewer(palette = "Dark2") +
  theme_bw() +
  theme(legend.key.size = unit(0.75, "cm")) +
  labs(x = "log(Minimum Distance Travelled)",
       y = "Parameter Name",
       title = "Estimated Distance Travelled on First Trial for Species and Individuals")

print(mu_posterior_plot)
```

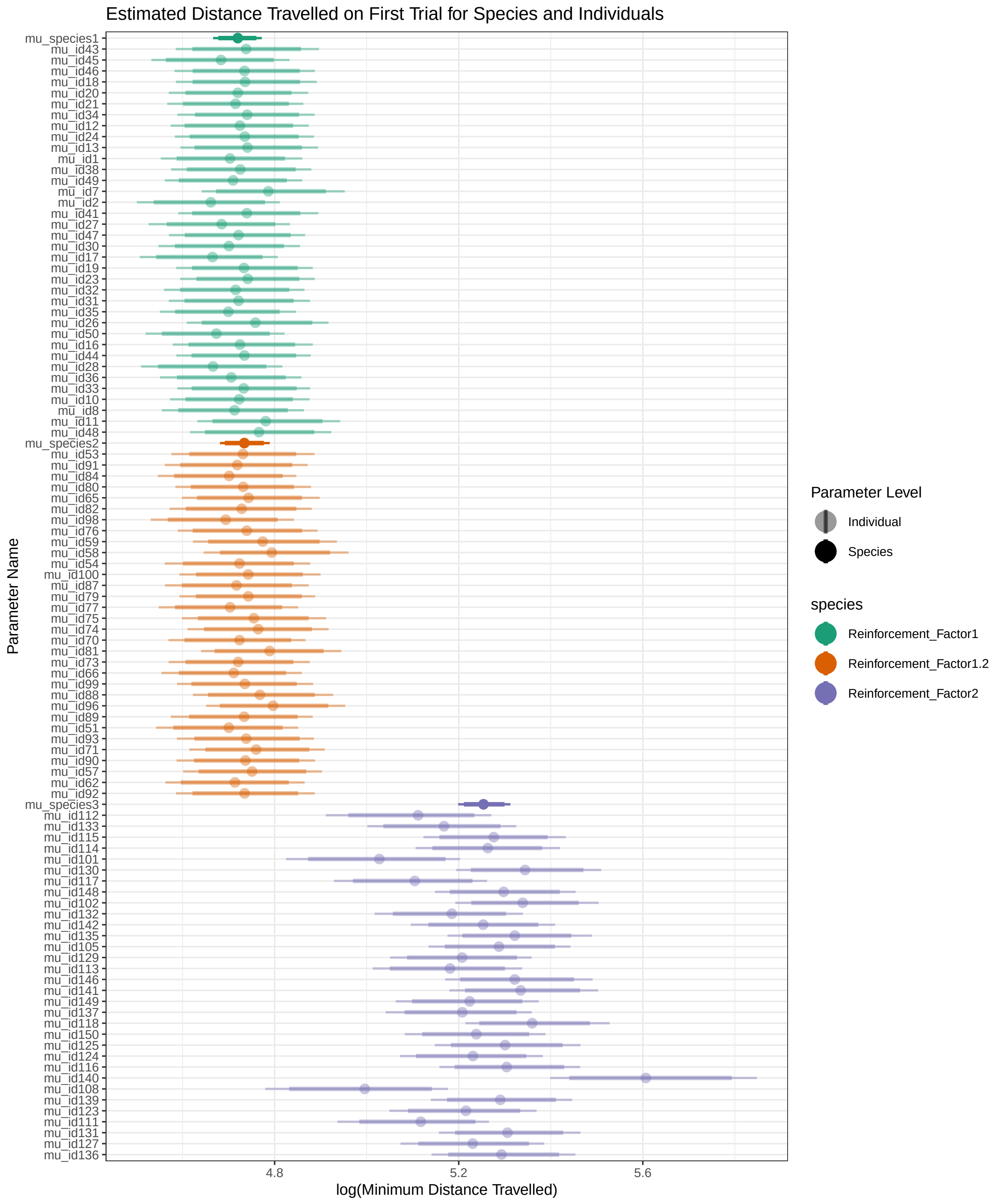


```
#plot slope (b) paramters
b_posterior_plot <- posterior_quantiles_sim_Zed_idSubset %>%
  filter(param_type %in% c("b_species", "b_id")) %>%
  arrange(species) %>%
  mutate(parameter = factor(parameter, levels = parameter[length(parameter):1])) %>%
  ggplot(aes(y = parameter)) +
  geom_pointrange(aes(x = X50,
                      xmin = X10,
```

```
                      xmax = X90,
                      alpha = param_type,
                      color = species),
                  orientation = "y", size = 1.5, fatten = 1.5) +
  geom_linerange(aes(xmin = X5,
                     xmax = X95,
                     alpha = param_type,
                     color = species),
                 orientation = "y", size = 0.8) +
  scale_alpha_discrete(range = c(.4, 1), name = "Parameter Level", labels = c("Individual", "Species")) +
  scale_color_brewer(palette = "Dark2") +
  geom_vline(xintercept = 0, color = "red", linetype = "dashed") +
  theme_bw() +
  theme(legend.key.size = unit(0.75, "cm")) +
  labs(x = "log(Distance)/log(Trial)",
       y = "Slope Parameter",
       title = "Estimated Learning Rates for Species and Individuals")

print(b_posterior_plot)
```

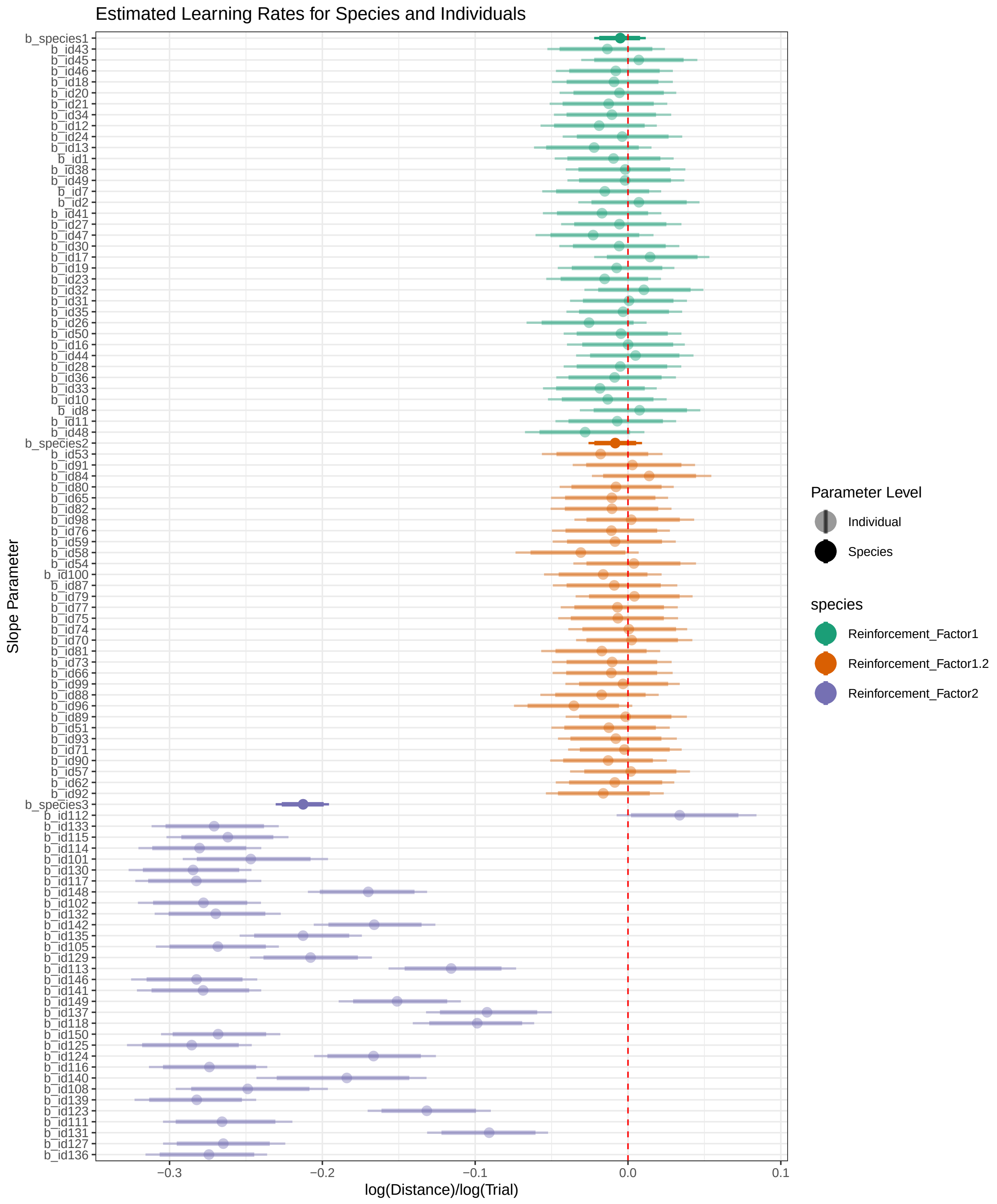

This results look very similar to the Double Trapezoid simulations. They have the same strong effect of reinforcement factor on learning rate, with an accompanying shift in estimated intercept indicating a mis-fit to the data. They also exhibit the high individual variance in paramter estimates for reinforcement factor 2 agents. Interestingly, a learning rate of 0 is contained in the 80% Credible Interval of reinforcement factor 1.2 agents, suggesting this level of reinforcement is not sufficient to learn shorter routes - a notable difference from the results in the Double Trapezoid.

```
## pdf
##   2
```

```
## pdf
##   2
```

```
Trial <- 1:max(Zed_data_sim$Trial)
pal <- brewer.pal(n = 8, name = "Dark2")

sample_set <- sample(1:4000, size = 100)


for (i in 1:3) { #loop over each species (column in posterior estimates)
  plot(1, type = "n", xlim = c(0, max(Zed_data_sim$Trial_scaled)), ylim = c(3.5,6), ylab = NA, xlab = NA, main = levels(Zed_data
  title(ylab = "log(Distance)", xlab = "log(Trial)", cex.lab = 1, line = 2)
  Distance_mean <- mean(posteriors_sim_Zed$mu_species[,i]) + mean(posteriors_sim_Zed$b_species[,i]) * log(Trial)
  lines(x = log(Trial), y = Distance_mean, col = "red", lwd = 3)
  for (j in sample_set) {
    lines(x = log(Trial), y = posteriors_sim_Zed$mu_species[j,i] + posteriors_sim_Zed$b_species[j,i] * log(Trial), col = alpha("
  }
  points(x = filter(Zed_data_sim, Species == levels(Zed_data_sim$Species)[i])$Trial_scaled,
         y = filter(Zed_data_sim, Species == levels(Zed_data_sim$Species)[i])$Distance_scaled,
         pch = 19, cex = 0.5, col = alpha(pal[i], alpha = 0.4))
}
```

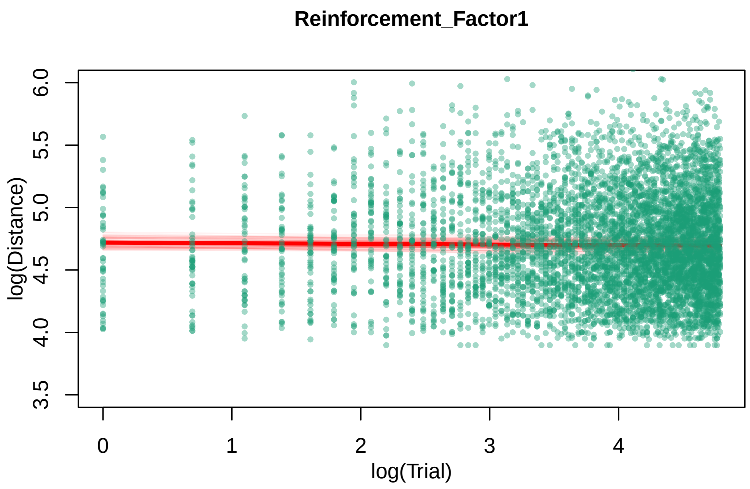

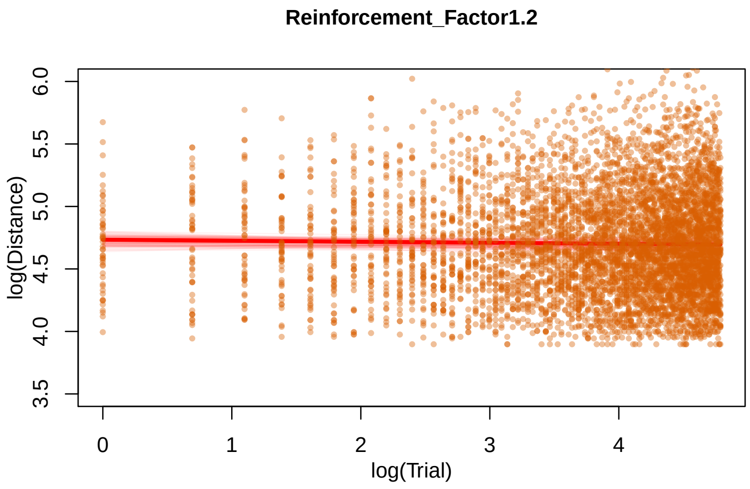

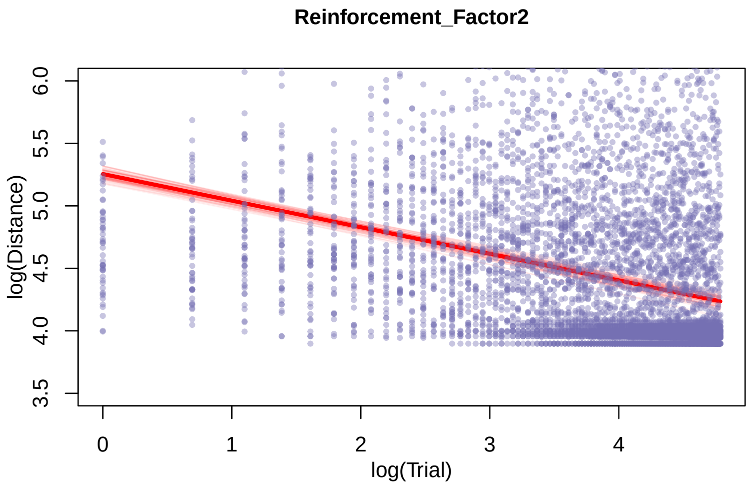

Integrated Analyses

First, we want to look at species level parameter estimates, for both real species and simulated agent types, within arrays.

```
#add metadata to quantiles
posterior_quantiles_DT <- mutate(posterior_quantiles_DT, array = "DT", type = "Empirical")
posterior_quantiles_Pentagon <- mutate(posterior_quantiles_Pentagon, array = "Pentagon", type = "Empiri
posterior_quantiles_Zed <- mutate(posterior_quantiles_Zed, array = "Zed", type = "Empirical")
posterior_quantiles_sim_DT <- mutate(posterior_quantiles_sim_DT, array = "DT", type = "Simulated")
posterior_quantiles_sim_Pentagon <- mutate(posterior_quantiles_sim_Pentagon, array = "Pentagon", type =
posterior_quantiles_sim_Zed <- mutate(posterior_quantiles_sim_Zed, array = "Zed", type = "Simulated")

#integrate quantiles from all analyses
all_posterior_quantiles <-  rbind(posterior_quantiles_DT, posterior_quantiles_Pentagon, posterior_quant
```

```
all_posterior_quantiles$species <- factor(all_posterior_quantiles$species, levels = c("Aye_Aye", "Dwarf_
all_posterior_quantiles$type <- factor(all_posterior_quantiles$type, levels = c("Empirical", "Simulated

#plot for Supplement
posteriors_plot <- all_posterior_quantiles %>%
  filter(param_type %in% c("mu_species", "b_species")) %>%
  arrange(species) %>%
  ggplot(aes(y = species)) + facet_grid(cols = vars(param_type), rows = vars(array) , scales = "free") +
  geom_pointrange(aes(x = X50,
                    xmin = X10,
                    xmax = X90,
                    color = type),
                orientation = "y", size = 1.5, fatten = 1.5) +
  geom_linerange(aes(xmin = X5,
                    xmax = X95,
                    color = type),
                orientation = "y", size = 0.8) +
  scale_y_discrete(limits = rev) +
  scale_color_brewer(palette = "Dark2") +
  theme_bw() +
  theme(legend.key.size = unit(0.75, "cm"),
        plot.title = element_text(hjust = 0.75)) +
  labs(x = "log(Minimum Distance Travelled)",
       y = "Species",
       title = "Posterior Estimates of Model Slope (b) and Intercept (mu)")

print(posteriors_plot)
```

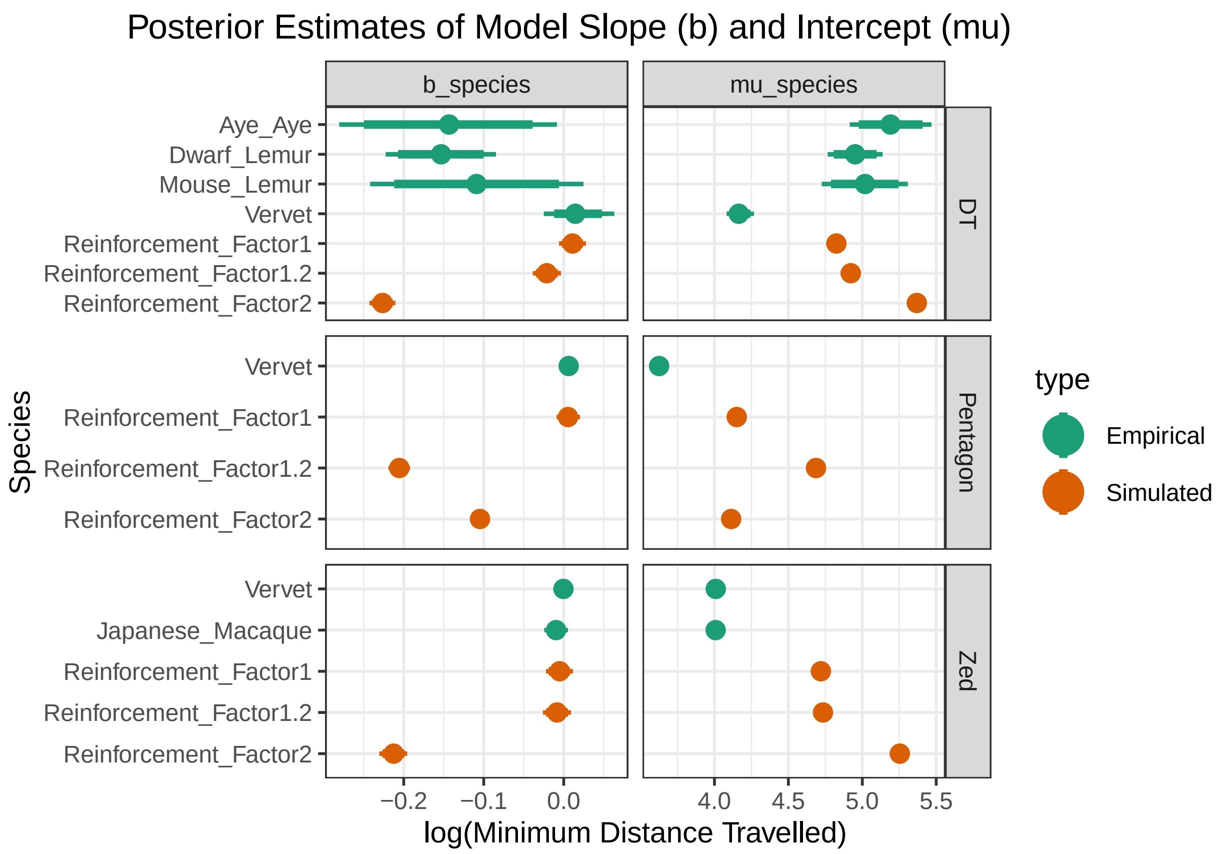


```
#plot for manuscript

tiff(filename = "../Docs/Figure 3_Credible Intervals.tif", width = 1200, height = 1000)
posteriors_plot <- all_posterior_quantiles %>%
  filter(param_type %in% c("mu_species", "b_species")) %>%
  arrange(species) %>%
  mutate(species = factor(gsub("_", " ", species),
                          levels = c("Aye Aye", "Dwarf Lemur", "Mouse Lemur", "Vervet", "Japanese Macaq
  ggplot(aes(y = species)) + facet_grid(cols = vars(param_type), rows = vars(array) , scales = "free") +
  geom_pointrange(aes(x = X50,
                      xmin = X10,
                      xmax = X90,
                      color = type),
                  orientation = "y", size = 3, fatten = 2) +
  geom_linerange(aes(xmin = X2.5,
                     xmax = X97.5,
                     color = type),
                 orientation = "y", size = 1.5) +
  scale_y_discrete(limits = rev) +
  scale_color_brewer(palette = "Dark2") +
  theme_bw() +
  theme(legend.key.size = unit(2, "cm"),
        plot.title = element_text(hjust = 0.5, vjust = 1.5),
        text = element_text(size = 28),
        axis.text.y = element_text(angle = 15)) +
  labs(x = "log(Minimum Distance Travelled)",
```

```
       y = "Species",
       title = "Credible Intervals of Model Slope (b) and Intercept (mu)")

print(posteriors_plot)
dev.off()
```

```
## pdf
##   2
```

Second, we look at the posterior regressions for the empirical data.

```
#Figure for Manuscript

Trial <- 1:max(DT_data$Trial)
pal <- brewer.pal(n = 8, name = "Dark2")
species <- c("Aye Aye", "Dwarf Lemur", "Mouse Lemur", "Vervet")
sample_set <- sample(1:4000, size = 100)
tiff("../Docs/Figure 2_Posterior_Predictions.tif", width = 1600, height = 1200)
layout(matrix(c(1,1,2,2,3,3,4,4,0,5,5,0,6,6,7,7), ncol = 8, byrow = TRUE))
par(mar = c(10,10,8,4))

for (i in 1:4) { #loop over each species (column in posterior estimates)
  plot(1, type = "n", xlim = c(0, max(DT_data$Trial_scaled)), ylim = c(4,6.5), ylab = NA, xlab = NA, ce
  title(ylab = "log(Distance)", xlab = "log(Trial)", cex.lab = 3, line = 4)
  title(main = species[i], line = -2, cex.main = 3)
  Distance_mean <- mean(posteriors_DT$mu_species[,i]) + mean(posteriors_DT$b_species[,i]) * log(Trial)
  lines(x = log(Trial), y = Distance_mean, col = pal[i], lwd = 3)
  for (j in sample_set) {
    lines(x = log(Trial), y = posteriors_DT$mu_species[j,i] + posteriors_DT$b_species[j,i] * log(Trial)
  }
  species_data <- filter(DT_data, Species == gsub(" ", "_", species[i]))
  points(x = species_data$Trial_scaled, y = species_data$Distance_scaled, col = pal[i], pch = 19, cex =
}

Trial <- 1:max(Pentagon_data$Trial)
pal <- brewer.pal(n = 8, name = "Dark2")
species <- c("Vervet")
sample_set <- sample(1:4000, size = 100)

#par(mar = c(10,10,10,4))

for (i in 1:1) { #loop over each species (column in posterior estimates)
  plot(1, type = "n", xlim = c(0, max(Pentagon_data$Trial_scaled)), ylim = c(3.5,4), ylab = NA, xlab = 
  title(ylab = "log(Distance)", xlab = "log(Trial)", cex.lab = 3, line = 4)
  title(main = species[i], line = -2, cex.main = 3)
  Distance_mean <- mean(posteriors_Pentagon$mu_species[,i]) + mean(posteriors_Pentagon$b_species[,i]) *
  lines(x = log(Trial), y = Distance_mean, col = pal[4], lwd = 3)
  for (j in sample_set) {
    lines(x = log(Trial), y = posteriors_Pentagon$mu_species[j,i] + posteriors_Pentagon$b_species[j,i] 
  }
  species_data <- filter(Pentagon_data, Species == gsub(" ", "_", species[i]))
  points(x = species_data$Trial_scaled, y = species_data$Distance_scaled, col = pal[4], pch = 19, cex =
}
```

```
Trial <- 1:max(Zed_data$Trial)
pal <- brewer.pal(n = 8, name = "Dark2")
species <- c("Japanese Macaque", "Vervet")
sample_set <- sample(1:4000, size = 100)



for (i in 1:2) { #loop over each species (column in posterior estimates)
  plot(1, type = "n", xlim = c(0, max(Zed_data$Trial_scaled)), ylim = c(3.8,4.5), ylab = NA, xlab = NA,
  title(ylab = "log(Distance)", xlab = "log(Trial)", cex.lab = 3, line = 4)
  title(main = species[i], line = -2, cex.main = 3)
  Distance_mean <- mean(posteriors_Zed$mu_species[,i]) + mean(posteriors_Zed$b_species[,i]) * log(Trial
  lines(x = log(Trial), y = Distance_mean, col = pal[6-i], lwd = 3)
  for (j in sample_set) {
    lines(x = log(Trial), y = posteriors_Zed$mu_species[j,i] + posteriors_Zed$b_species[j,i] * log(Tria
  }
  species_data <- filter(Zed_data, Species == gsub(" ", "_", species[i]))
  points(x = species_data$Trial_scaled, y = species_data$Distance_scaled, col = pal[6-i], pch = 19, cex
}

par(xpd = NA) #removes gridding of layout for placing geometries across whole plot
#box(which = "figure", col = "red", lwd = 3)
#DT Section
rect(-27,4.7,5.85,5.71, border = "grey30", lwd = 8)
text(x = -10, y = 5.65, labels = "Double Trapezoid", col = "grey30", cex = 6)

#Pentagon Section
rect(-27, 3.62, -10.35, 4.68, border = "grey30", lwd = 8)
text(x = -18.3, y = 4.6, labels = "Pentagon", cex = 6, col = "grey30")

#Zed Section
rect(-10.1, 3.62, 5.85, 4.68, border = "grey30", lwd = 8)
text(x = -1.7, y = 4.6, labels = "Z-Array", cex = 6, col = "grey30")

dev.off()
```

```
## pdf
##   2
```

And then the regression plots for the simulations

```
tiff("../Docs/Figure 4_Posterior_Regressions_Simulations.tif", width = 1600, height = 1200)
par(mfrow = c(3,3),
    mar = c(6,7,10,2))

Trial <- 1:max(DT_data_sim$Trial)
pal <- brewer.pal(n = 8, name = "Dark2")

sample_set <- sample(1:4000, size = 100)

for (i in 1:3) { #loop over each species (column in posterior estimates)
  plot(1, type = "n", xlim = c(0, max(DT_data_sim$Trial_scaled)), ylim = c(3.5,7), ylab = NA, xlab = NA
```

```
  title(ylab = "log(Distance)", xlab = "log(Trial)", cex.lab = 3.5, line = 4)
    title(main = gsub("_", " ", levels(Pentagon_data_sim$Species)[i]), line = -2.8, cex.main = 4, col =
  Distance_mean <- mean(posteriors_sim_DT$mu_species[,i]) + mean(posteriors_sim_DT$b_species[,i]) * log
  lines(x = log(Trial), y = Distance_mean, col = "red", lwd = 3)
  points(x = filter(DT_data_sim, Species == levels(DT_data_sim$Species)[i])$Trial_scaled,
         y = filter(DT_data_sim, Species == levels(DT_data_sim$Species)[i])$Distance_scaled,
         pch = 19, cex = 1, col = alpha(pal[i], alpha = 0.1))
  for (j in sample_set) {
    lines(x = log(Trial), y = posteriors_sim_DT$mu_species[j,i] + posteriors_sim_DT$b_species[j,i] * log
          col = alpha("red", alpha = 0.05), lwd = 1)
  }
}

Trial <- 1:max(Pentagon_data_sim$Trial)
pal <- brewer.pal(n = 8, name = "Dark2")

for (i in 1:3) { #loop over each species (column in posterior estimates)
  plot(1, type = "n", xlim = c(0, max(Pentagon_data_sim$Trial_scaled)), ylim = c(3.5,7), ylab = NA, xlab
  title(ylab = "log(Distance)", xlab = "log(Trial)", cex.lab = 3.5, line = 4)
    title(main = gsub("_", " ", levels(Pentagon_data_sim$Species)[i]), line = -2.8, cex.main = 4, col =
  Distance_mean <- mean(posteriors_sim_Pentagon$mu_species[,i]) + mean(posteriors_sim_Pentagon$b_species
  lines(x = log(Trial), y = Distance_mean, col = "red", lwd = 3)
  points(x = filter(Pentagon_data_sim, Species == levels(Pentagon_data_sim$Species)[i])$Trial_scaled,
         y = filter(Pentagon_data_sim, Species == levels(Pentagon_data_sim$Species)[i])$Distance_scaled
         pch = 19, cex = 1, col = alpha(pal[i], alpha = 0.1))
  for (j in sample_set) {
    lines(x = log(Trial), y = posteriors_sim_Pentagon$mu_species[j,i] + posteriors_sim_Pentagon$b_specie
          col = alpha("red", alpha = 0.05), lwd = 1)
  }
}



Trial <- 1:max(Zed_data_sim$Trial)
pal <- brewer.pal(n = 8, name = "Dark2")

for (i in 1:3) { #loop over each species (column in posterior estimates)
  plot(1, type = "n", xlim = c(0, max(Zed_data_sim$Trial_scaled)), ylim = c(3.5,7), ylab = NA, xlab = NA
  title(ylab = "log(Distance)", xlab = "log(Trial)", cex.lab = 3.5, line = 4)
  title(main = gsub("_", " ", levels(Zed_data_sim$Species)[i]), line = -2.8, cex.main = 4, col = "grey30
  Distance_mean <- mean(posteriors_sim_Zed$mu_species[,i]) + mean(posteriors_sim_Zed$b_species[,i]) * lo
  lines(x = log(Trial), y = Distance_mean, col = "red", lwd = 3)
  points(x = filter(Zed_data_sim, Species == levels(Zed_data_sim$Species)[i])$Trial_scaled,
         y = filter(Zed_data_sim, Species == levels(Zed_data_sim$Species)[i])$Distance_scaled,
         pch = 19, cex = 1, col = alpha(pal[i], alpha = 0.1))
  for (j in sample_set) {
    lines(x = log(Trial), y = posteriors_sim_Zed$mu_species[j,i] + posteriors_sim_Zed$b_species[j,i] * l
          col = alpha("red", alpha = 0.05), lwd = 1)
  }
}

par(xpd = NA)
text(x = -10.8, y = 20, labels = "Double Trapezoid", col = "grey30", cex = 6)
```

```r
text(x = -11.8, y = 13.9, labels = "Pentagon", cex = 6, col = "grey30")
text(x = -12, y = 7.9, labels = "Z-Array", cex = 6, col = "grey30")
dev.off()
```

```
## pdf
##   2
```
