# Supplementary material for: Mild movement sequence repetition in five primate species and evidence for a taxonomic divide in cognitive mechanisms
Source: Sci Rep. 2022 Aug 25;12:14503. doi: 10.1038/s41598-022-18633-7 (PMC9411198; doi:10.1038/s41598-022-18633-7)
Supplement: Supplementary file 4 — Supplementary Information 4. [file 41598_2022_18633_MOESM4_ESM.html]

Lemur Path Recursion: DET Analysis


# Lemur Path Recursion: DET Analysis

#### Alexander Vining

#### 6/6/2022

# Setup

```
library(readr)
library(tidyr)
library(dplyr)
library(lme4)
library(rstan)
library(bayesplot)
library(ggplot2)
library(ggridges)

DETs <- read_csv("../Results/data_full.csv")
```

# Modelling determinism (DET) of lemur navigation through multi-destination arrays

The full data set contains the number of recursions in each trial for
all empirical and simulated data. A recursion is a match between a visit
to a station and ANY previous visit by the same individual. Thus, an
individual’s 10th visit to a station will have 9 associated recursions.
The DET score for each trial is the proportion of recursions for that
trial that are repeats, meaning the associated pair of visits to a given
location occurred within an identical sequence of three location visits.
Because this metric is sensitive to the length of a sequence (both the
segment under analysis, and the sequence preceding that segment), we do
our best to standardize comparisons by only analyzing the first ten
trials for all individuals, and only including individual that completed
ten trials.

```
DET_subset <- DETs %>% group_by(ID, Array) %>% filter(max(Trial) >=10, Trial <= 10)
summarize(DET_subset, len = n()) %>% summary() #check all groups have length of 10
```

```
## `summarise()` has grouped output by 'ID'. You can override using the `.groups`
## argument.
```

```
##       ID               Array                len    
##  Length:3040        Length:3040        Min.   :10  
##  Class :character   Class :character   1st Qu.:10  
##  Mode  :character   Mode  :character   Median :10  
##                                        Mean   :10  
##                                        3rd Qu.:10  
##                                        Max.   :10
```

## Bayesian Models

### Double Trapezoid

```
DETs_DT <- DET_subset %>% 
  filter(Array == "DT" & ! Species == "None") %>% 
  mutate(ID = factor(ID), Species = factor(Species), Source = factor(Source)) %>% #if rerun, re-order Source factors so experimental is first
  select(!Session) %>% 
  na.omit()

STAN_DETdata_DT <- list(N = nrow(DETs_DT),
                        I = length(levels(DETs_DT$ID)),
                        J = length(levels(DETs_DT$Species)),
                        K = length(levels(DETs_DT$Source)),
                        Rep = as.integer(DETs_DT$DET * DETs_DT$Recursions),
                        w = DETs_DT$Recursions,
                        ID = as.numeric(DETs_DT$ID),
                        Sp = as.numeric(DETs_DT$Species),
                        Src = as.numeric(DETs_DT$Source))
```

We tested this data on a simpler model

```
options(mc.cores = parallel::detectCores())
fit_DET_binom_DT <- stan(file = "../CODE/DET_Binom_model.stan",
                          data = STAN_DETdata_DT,
                          chains = 4,
                          iter = 2000,
                          warmup = 1000)

save(fit_DET_binom_DT, file = "../Results/DET_DT_bimonmodel.Rdata") #This currently does not include individual effects in estimating likelihood.
```

And the model appears healthy . . .

```
load("../Results/DET_DT_bimonmodel.Rdata")
print(fit_DET_binom_DT, pars = c("a_0", "a_Sp", "a_Src"))
```

```
## Inference for Stan model: DET_Binom_model.
## 4 chains, each with iter=2000; warmup=1000; thin=1; 
## post-warmup draws per chain=1000, total post-warmup draws=4000.
## 
##           mean se_mean   sd  2.5%   25%   50%   75% 97.5% n_eff Rhat
## a_0      -0.24    0.03 0.58 -1.38 -0.63 -0.22  0.15  0.93   369 1.01
## a_Sp[1]  -0.05    0.02 0.46 -0.98 -0.35 -0.05  0.27  0.87   393 1.00
## a_Sp[2]  -0.14    0.02 0.46 -1.07 -0.44 -0.14  0.18  0.79   393 1.00
## a_Sp[3]  -0.10    0.02 0.46 -1.03 -0.41 -0.11  0.21  0.81   393 1.00
## a_Sp[4]  -0.07    0.02 0.46 -0.99 -0.38 -0.07  0.25  0.85   393 1.00
## a_Src[1] -0.41    0.02 0.45 -1.27 -0.71 -0.42 -0.09  0.48   348 1.01
## a_Src[2] -0.27    0.02 0.45 -1.13 -0.57 -0.28  0.04  0.62   348 1.01
## a_Src[3]  0.36    0.02 0.45 -0.50  0.06  0.36  0.68  1.25   348 1.01
## a_Src[4]  0.12    0.02 0.45 -0.75 -0.18  0.11  0.43  1.00   349 1.01
## 
## Samples were drawn using NUTS(diag_e) at Mon Jun 13 00:22:46 2022.
## For each parameter, n_eff is a crude measure of effective sample size,
## and Rhat is the potential scale reduction factor on split chains (at 
## convergence, Rhat=1).
```

```
pairs(fit_DET_binom_DT, pars = c("a_0", "a_Sp", "a_Src"), las = 1)
```

Next, we fit a more complex model allowing interactions between
species and source effects, and individual effects.

```
options(mc.cores = parallel::detectCores())
fit_DET_binom_DT_Interact <- stan(file = "../CODE/DET_Binom_model_interactions.stan",
                          data = STAN_DETdata_DT,
                          chains = 4,
                          iter = 2000,
                          warmup = 1000)

save(fit_DET_binom_DT_Interact, file = "../Results/DET_DT_bimonmodel_interactions.Rdata") #This currently does not include individual effects.
```

This model yielded some warning about max-tree depth, which is an
efficiency issue (indeed, it took about 10 hours to run), but should not
impact our inferences. It also warned of some high rhat values, which
could indicate a failure to converge. Looking at the rhat values of the
important parameters, though, they all seem healthy. This indicates poor
convergence occurred only in the individual effects, which are not
relevant to our model interpretations and will be integrated over in the
posterior.

```
load("../Results/DET_DT_bimonmodel_interactions.Rdata")
print(fit_DET_binom_DT_Interact, pars = c("a_0", "a_Sp", "a_Src", "a_Interact"))
```

```
## Inference for Stan model: DET_Binom_model_interactions.
## 4 chains, each with iter=2000; warmup=1000; thin=1; 
## post-warmup draws per chain=1000, total post-warmup draws=4000.
## 
##                 mean se_mean   sd  2.5%   25%   50%  75% 97.5% n_eff Rhat
## a_0            -0.34    0.02 0.59 -1.48 -0.74 -0.34 0.04  0.84   593 1.00
## a_Sp[1]        -0.03    0.03 0.60 -1.21 -0.43 -0.02 0.39  1.13   395 1.01
## a_Sp[2]        -0.05    0.03 0.60 -1.22 -0.47 -0.05 0.34  1.13   500 1.01
## a_Sp[3]        -0.13    0.03 0.59 -1.24 -0.54 -0.14 0.28  1.04   441 1.01
## a_Sp[4]         0.03    0.03 0.62 -1.15 -0.39  0.03 0.44  1.26   490 1.01
## a_Src[1]       -0.34    0.03 0.62 -1.62 -0.76 -0.33 0.11  0.78   505 1.00
## a_Src[2]       -0.25    0.03 0.59 -1.41 -0.66 -0.25 0.16  0.87   511 1.01
## a_Src[3]        0.26    0.03 0.59 -0.90 -0.15  0.26 0.67  1.39   483 1.00
## a_Src[4]        0.04    0.03 0.61 -1.17 -0.36  0.06 0.45  1.19   527 1.00
## a_Interact[1]  -0.02    0.02 0.58 -1.13 -0.43 -0.02 0.38  1.15   560 1.00
## a_Interact[2]   0.02    0.03 0.60 -1.15 -0.37  0.03 0.43  1.17   458 1.01
## a_Interact[3]   0.11    0.03 0.57 -0.99 -0.27  0.10 0.49  1.21   408 1.00
## a_Interact[4]  -0.08    0.03 0.60 -1.17 -0.51 -0.10 0.33  1.14   441 1.00
## a_Interact[5]  -0.08    0.02 0.57 -1.24 -0.46 -0.07 0.30  0.96   545 1.00
## a_Interact[6]  -0.09    0.03 0.60 -1.26 -0.48 -0.10 0.31  1.14   444 1.01
## a_Interact[7]   0.01    0.03 0.60 -1.12 -0.40  0.00 0.41  1.22   414 1.00
## a_Interact[8]  -0.04    0.02 0.58 -1.15 -0.42 -0.05 0.35  1.10   590 1.00
## a_Interact[9]  -0.04    0.03 0.59 -1.18 -0.43 -0.05 0.33  1.18   516 1.00
## a_Interact[10]  0.03    0.03 0.60 -1.19 -0.36  0.05 0.43  1.16   496 1.01
## a_Interact[11]  0.19    0.03 0.60 -0.95 -0.23  0.18 0.59  1.37   455 1.00
## a_Interact[12] -0.18    0.03 0.63 -1.39 -0.61 -0.19 0.23  1.10   499 1.01
## a_Interact[13] -0.14    0.03 0.60 -1.30 -0.54 -0.15 0.27  1.05   495 1.01
## a_Interact[14] -0.08    0.03 0.61 -1.22 -0.51 -0.11 0.34  1.11   507 1.01
## a_Interact[15] -0.11    0.03 0.61 -1.33 -0.51 -0.09 0.30  1.09   435 1.01
## a_Interact[16]  0.32    0.03 0.63 -0.85 -0.13  0.31 0.75  1.55   433 1.01
## 
## Samples were drawn using NUTS(diag_e) at Tue Jun 14 12:29:22 2022.
## For each parameter, n_eff is a crude measure of effective sample size,
## and Rhat is the potential scale reduction factor on split chains (at 
## convergence, Rhat=1).
```

```
pairs(fit_DET_binom_DT_Interact, pars = c("a_0", "a_Sp", "a_Src"), las = 1)
```

```
#print(fit_DET_binom_DT, pars = c("a_id"))
```

We also look at some pairs plots and ensure that there are no
worrisome correlation ridges.

Because we set up the stan model to automatically generate
factor-specific estimates of alpha, we can extract those values directly
and then label them using the same iterative process as the STAN model
does.

```
a_pred <- rstan::extract(fit_DET_binom_DT_Interact, "a_pred")[[1]] #a (short for alpha) was used to represent to probability of success in our binomial model. It is equivalent to DET
#Assign conditions to predictions by iterating through species and sources, as in the STAN model
i <- 0
interaction_factors <- vector(length = ncol(a_pred))
for(j in levels(DETs_DT$Species)){
  for(k in levels(DETs_DT$Source)){
    i <- i+1
    interaction_factors[i] <- paste(j,k, sep = "X") #sep value used for parsing into factors later
  }
}
colnames(a_pred) <- interaction_factors
```

Finally, we can plot the posterior distributions of the alpha
estimates, allowing us to make inferences as described in the results
section of the associated manuscript “Mild movement sequence repetition
in five primate species and evidence for a taxonomic divide in cognitive
mechanisms”

```
pal <- c("#E69F00", "#56B4E9", "#009E73", "#F0E442")
plot_colors <- rep(pal, each = 4)
names(plot_colors) <- colnames(a_pred)

base_plot <- a_pred %>% 
  data.frame() %>%
  pivot_longer(cols = everything(), names_to = c("Species", "Source"), names_sep = "X", values_to = "alpha") %>%
  mutate(Source = factor(Source, levels = c("Learning_Factor1", "Learning_Factor1.2", "Learning_Factor2", "Experimental"))) %>% 
  ggplot()

base_plot <- base_plot + 
  geom_density_ridges(aes(x = alpha, y = interaction(Source, Species), fill = Species), rel_min_height = 0.01) +
  theme_classic() +
  theme(panel.grid.major.y = element_line(color = "grey", linetype = "dotted")) +
  geom_vline(aes(xintercept = 0),color = "grey", linetype = "dotted")  +
  scale_y_discrete(labels = c("Learning Factor 1", "Learning Factor 1.2", "Learning Factor 2", "Empirical", "Learning Factor 1", "Learning Factor 1.2", "Learning Factor 2", "Empirical", "Learning Factor 1", "Learning Factor 1.2", "Learning Factor 2", "Empirical", "Learning Factor 1", "Learning Factor 1.2", "Learning Factor 2", "Empirical")) +
  scale_fill_manual(values = pal, labels = c("Aye Aye", "Dwarf Lemur", "Mouse Lemur", "Vervet")) +
  labs(y = "Source", x = "logit(DET)")

ggsave(file = "../Results/Credible Intervals of alpha.tiff",
       plot = base_plot,
       units = "px",
       dpi = 300)
```

```
## Saving 2100 x 1500 px image
```

```
## Picking joint bandwidth of 0.0125
```

```
base_plot
```

```
## Picking joint bandwidth of 0.0125
```

### Zed\_Array

```
DETs_Zed_Array <- DET_subset %>% 
  filter(Array == "Zarray" & ! Species == "None") %>% 
  mutate(ID = factor(ID), Species = factor(Species), Source = factor(Source)) %>% #if rerun, re-order Source factors so experimental is first
  select(!Session) %>% 
  na.omit()

STAN_DETdata_Zed_Array <- list(N = nrow(DETs_Zed_Array),
                        I = length(levels(DETs_Zed_Array$ID)),
                        J = length(levels(DETs_Zed_Array$Species)),
                        K = length(levels(DETs_Zed_Array$Source)),
                        Rep = as.integer(DETs_Zed_Array$DET * DETs_Zed_Array$Recursions),
                        w = DETs_Zed_Array$Recursions,
                        ID = as.numeric(DETs_Zed_Array$ID),
                        Sp = as.numeric(DETs_Zed_Array$Species),
                        Src = as.numeric(DETs_Zed_Array$Source))
```

We fit a model allowing interactions between species and source
effects, and individual effects.

```
options(mc.cores = parallel::detectCores())
fit_DET_binom_Zed_Array_Interact <- stan(file = "../CODE/DET_Binom_model_interactions.stan",
                          data = STAN_DETdata_Zed_Array,
                          chains = 4,
                          iter = 2000,
                          warmup = 1000)

save(fit_DET_binom_Zed_Array_Interact, file = "../Results/DET_Zed_Array_bimonmodel_interactions.Rdata")
```

This model yielded some warning about max-tree depth, which should
not impact our inferences. It also warned of low Bulk Effective Sample
size and high Rhats, which could indicate poor mixing and lack of
convergence. Looking at the ESS values and Rhats of the important
parameters, though, they all seem more than sufficient. This indicates
the problem is in the estimation of individual effects, which are not
relevant to our model interpretations and will be integrated over in the
posterior.

```
load("../Results/DET_Zed_Array_bimonmodel_interactions.Rdata")
print(fit_DET_binom_Zed_Array_Interact, pars = c("a_0", "a_Sp", "a_Src", "a_Interact"))
```

```
## Inference for Stan model: anon_model.
## 4 chains, each with iter=2000; warmup=1000; thin=1; 
## post-warmup draws per chain=1000, total post-warmup draws=4000.
## 
##                mean se_mean   sd  2.5%   25%   50%  75% 97.5% n_eff Rhat
## a_0           -0.20    0.02 0.67 -1.47 -0.65 -0.22 0.23  1.12  1015    1
## a_Sp[1]       -0.20    0.02 0.67 -1.52 -0.65 -0.20 0.23  1.09  1013    1
## a_Sp[2]       -0.09    0.02 0.67 -1.38 -0.55 -0.10 0.36  1.23   998    1
## a_Src[1]      -0.38    0.02 0.68 -1.73 -0.84 -0.37 0.08  0.95  1013    1
## a_Src[2]      -0.33    0.02 0.67 -1.70 -0.78 -0.32 0.12  1.01   984    1
## a_Src[3]       0.09    0.02 0.68 -1.25 -0.37  0.09 0.55  1.40   963    1
## a_Src[4]       0.31    0.02 0.70 -1.02 -0.16  0.30 0.78  1.72  1000    1
## a_Interact[1] -0.20    0.02 0.68 -1.50 -0.67 -0.19 0.26  1.13   967    1
## a_Interact[2] -0.16    0.02 0.69 -1.51 -0.64 -0.16 0.30  1.16   999    1
## a_Interact[3]  0.11    0.02 0.69 -1.23 -0.37  0.12 0.56  1.42   977    1
## a_Interact[4] -0.01    0.02 0.70 -1.36 -0.49  0.00 0.48  1.35   992    1
## a_Interact[5] -0.29    0.02 0.69 -1.59 -0.75 -0.30 0.15  1.12  1042    1
## a_Interact[6] -0.24    0.02 0.68 -1.62 -0.70 -0.23 0.21  1.07  1104    1
## a_Interact[7] -0.03    0.02 0.69 -1.40 -0.50 -0.02 0.44  1.29  1022    1
## a_Interact[8]  0.34    0.02 0.69 -0.98 -0.13  0.32 0.80  1.74  1029    1
## 
## Samples were drawn using NUTS(diag_e) at Thu Aug 11 13:42:03 2022.
## For each parameter, n_eff is a crude measure of effective sample size,
## and Rhat is the potential scale reduction factor on split chains (at 
## convergence, Rhat=1).
```

```
pairs(fit_DET_binom_Zed_Array_Interact, pars = c("a_0", "a_Sp", "a_Src"), las = 1)
```

```
#print(fit_DET_binom_Zed_Array, pars = c("a_id"))
```

We also look at some pairs plots and ensure that there are no
worrisome correlation ridges.

Because we set up the stan model to automatically generate
factor-specific estimates of alpha, we can extract those values directly
and then label them using the same iterative process as the STAN model
does.

```
a_pred <- rstan::extract(fit_DET_binom_Zed_Array_Interact, "a_pred")[[1]] #a (short for alpha) was used to represent to probability of success in our binomial model. It is equivalent to DET
#Assign conditions to predictions by iterating through species and sources, as in the STAN model
i <- 0
interaction_factors <- vector(length = ncol(a_pred))
for(j in levels(DETs_Zed_Array$Species)){
  for(k in levels(DETs_Zed_Array$Source)){
    i <- i+1
    interaction_factors[i] <- paste(j,k, sep = "X") #sep value used for parsing into factors later
  }
}
colnames(a_pred) <- interaction_factors
```

Finally, we can plot the posterior distributions of the alpha
estimates, allowing us to make inferences as described in the results
section of the associated manuscript “Mild movement sequence repetition
in five primate species and evidence for a taxonomic divide in cognitive
mechanisms”

```
pal <- c("#E69F00", "#56B4E9", "#009E73", "#F0E442")
plot_colors <- rep(pal, each = 4)
names(plot_colors) <- colnames(a_pred)

base_plot <- a_pred %>% 
  data.frame() %>%
  pivot_longer(cols = everything(), names_to = c("Species", "Source"), names_sep = "X", values_to = "alpha") %>%
  mutate(Source = factor(Source, levels = c("Learning_Factor1", "Learning_Factor1.2", "Learning_Factor2", "Experimental"))) %>% 
  ggplot()

base_plot <- base_plot + 
  geom_density_ridges(aes(x = alpha, y = interaction(Source, Species), fill = Species), rel_min_height = 0.01) +
  theme_classic() +
  theme(panel.grid.major.y = element_line(color = "grey", linetype = "dotted")) +
  geom_vline(aes(xintercept = 0),color = "grey", linetype = "dotted")  +
  scale_y_discrete(labels = c("Learning Factor 1", "Learning Factor 1.2", "Learning Factor 2", "Empirical", "Learning Factor 1", "Learning Factor 1.2", "Learning Factor 2", "Empirical", "Learning Factor 1", "Learning Factor 1.2", "Learning Factor 2", "Empirical", "Learning Factor 1", "Learning Factor 1.2", "Learning Factor 2", "Empirical")) +
  scale_fill_manual(values = pal, labels = c("Japanese Macaque", "Vervet")) +
  labs(y = "Source", x = "logit(DET)")

ggsave(file = "../Results/Credible Intervals of alpha_Zed_Array.tiff",
       plot = base_plot,
       units = "px",
       dpi = 300)
```

```
## Saving 2100 x 1500 px image
```

```
## Picking joint bandwidth of 0.00973
```

```
base_plot
```

```
## Picking joint bandwidth of 0.00973
```

### Pentagon

```
DETs_Pentagon <- DET_subset %>% 
  filter(Array == "Pentagon" & ! Species == "None") %>% 
  mutate(ID = factor(ID), Species = factor(Species), Source = factor(Source)) %>% #if rerun, re-order Source factors so experimental is first
  select(!Session) %>% 
  na.omit()

STAN_DETdata_Pentagon <- list(N = nrow(DETs_Pentagon),
                        I = length(levels(DETs_Pentagon$ID)),
                        J = length(levels(DETs_Pentagon$Species)),
                        K = length(levels(DETs_Pentagon$Source)),
                        Rep = as.integer(DETs_Pentagon$DET * DETs_Pentagon$Recursions),
                        w = DETs_Pentagon$Recursions,
                        ID = as.numeric(DETs_Pentagon$ID),
                        Sp = as.numeric(DETs_Pentagon$Species),
                        Src = as.numeric(DETs_Pentagon$Source))
```

We tested this data on a simpler model

```
options(mc.cores = parallel::detectCores())
fit_DET_binom_Pentagon <- stan(file = "../CODE/DET_Binom_model.stan",
                          data = STAN_DETdata_Pentagon,
                          chains = 4,
                          iter = 2000,
                          warmup = 1000)

save(fit_DET_binom_Pentagon, file = "../Results/DET_Pentagon_bimonmodel.Rdata")
```

As with the Double Trapezoid analysis, there is some failure to mix,
however the Rhats of important variables remain near 1, suggesting
estimation problems occur only in individual variation where it is of
less concern. Otherwise, the model appears healthy . . .

```
load("../Results/DET_Pentagon_bimonmodel.Rdata")
print(fit_DET_binom_Pentagon, pars = c("a_0", "a_Sp", "a_Src"))
```

```
## Inference for Stan model: anon_model.
## 4 chains, each with iter=2000; warmup=1000; thin=1; 
## post-warmup draws per chain=1000, total post-warmup draws=4000.
## 
##           mean se_mean   sd  2.5%   25%   50%   75% 97.5% n_eff Rhat
## a_0      -0.09    0.03 0.75 -1.49 -0.61 -0.09  0.43  1.37   482 1.02
## a_Sp[1]  -0.07    0.03 0.74 -1.45 -0.57 -0.08  0.45  1.37   683 1.01
## a_Src[1] -0.72    0.02 0.45 -1.62 -1.01 -0.71 -0.44  0.20   555 1.01
## a_Src[2] -0.67    0.02 0.45 -1.56 -0.95 -0.66 -0.38  0.24   554 1.01
## a_Src[3]  0.12    0.02 0.45 -0.79 -0.17  0.12  0.40  1.03   555 1.01
## a_Src[4]  1.20    0.02 0.45  0.29  0.90  1.20  1.48  2.10   561 1.01
## 
## Samples were drawn using NUTS(diag_e) at Wed Aug 10 17:13:04 2022.
## For each parameter, n_eff is a crude measure of effective sample size,
## and Rhat is the potential scale reduction factor on split chains (at 
## convergence, Rhat=1).
```

```
pairs(fit_DET_binom_Pentagon, pars = c("a_0", "a_Sp", "a_Src"), las = 1)
```

Next, we fit a more complex model allowing interactions between
species and source effects, and individual effects.

```
options(mc.cores = parallel::detectCores())
fit_DET_binom_Pentagon_Interact <- stan(file = "../CODE/DET_Binom_model_interactions.stan",
                          data = STAN_DETdata_Pentagon,
                          chains = 4,
                          iter = 2000,
                          warmup = 1000)

save(fit_DET_binom_Pentagon_Interact, file = "../Results/DET_Pentagon_bimonmodel_interactions.Rdata")
```

This model yielded some warning about max-tree depth, which is an
efficiency issue (indeed, it took about 10 hours to run), but should not
impact our inferences. It also warned of low Bulk Effective Sample size,
which could indicate poor mixing. Looking at the ESS values of the
important parameters, though, they all seem more than sufficient. This
indicatesthe problem is in the estimation of individual effects, which
are not relevant to our model interpretations and will be integrated
over in the posterior.

```
load("../Results/DET_Pentagon_bimonmodel_interactions.Rdata")
print(fit_DET_binom_Pentagon_Interact, pars = c("a_0", "a_Sp", "a_Src", "a_Interact"))
```

```
## Inference for Stan model: anon_model.
## 4 chains, each with iter=2000; warmup=1000; thin=1; 
## post-warmup draws per chain=1000, total post-warmup draws=4000.
## 
##                mean se_mean   sd  2.5%   25%   50%  75% 97.5% n_eff Rhat
## a_0           -0.06    0.01 0.79 -1.60 -0.59 -0.07 0.47  1.51  4665    1
## a_Sp[1]       -0.07    0.01 0.79 -1.58 -0.61 -0.06 0.45  1.47  5042    1
## a_Src[1]      -0.40    0.01 0.77 -1.91 -0.92 -0.41 0.13  1.04  5087    1
## a_Src[2]      -0.33    0.01 0.78 -1.88 -0.84 -0.34 0.19  1.18  5273    1
## a_Src[3]       0.05    0.01 0.75 -1.37 -0.46  0.05 0.56  1.51  5041    1
## a_Src[4]       0.62    0.01 0.76 -0.84  0.10  0.62 1.13  2.07  5305    1
## a_Interact[1] -0.37    0.01 0.76 -1.88 -0.90 -0.36 0.15  1.12  5003    1
## a_Interact[2] -0.38    0.01 0.75 -1.83 -0.90 -0.38 0.12  1.12  4392    1
## a_Interact[3]  0.04    0.01 0.76 -1.44 -0.47  0.04 0.55  1.53  3873    1
## a_Interact[4]  0.62    0.01 0.75 -0.81  0.12  0.60 1.13  2.16  5086    1
## 
## Samples were drawn using NUTS(diag_e) at Wed Aug 10 18:27:34 2022.
## For each parameter, n_eff is a crude measure of effective sample size,
## and Rhat is the potential scale reduction factor on split chains (at 
## convergence, Rhat=1).
```

```
pairs(fit_DET_binom_Pentagon_Interact, pars = c("a_0", "a_Sp", "a_Src"), las = 1)
```

```
## Warning in par(usr): argument 1 does not name a graphical parameter

## Warning in par(usr): argument 1 does not name a graphical parameter

## Warning in par(usr): argument 1 does not name a graphical parameter

## Warning in par(usr): argument 1 does not name a graphical parameter

## Warning in par(usr): argument 1 does not name a graphical parameter

## Warning in par(usr): argument 1 does not name a graphical parameter
```

```
#print(fit_DET_binom_Pentagon, pars = c("a_id"))
```

We also look at some pairs plots and ensure that there are no
worrisome correlation ridges.

Because we set up the stan model to automatically generate
factor-specific estimates of alpha, we can extract those values directly
and then label them using the same iterative process as the STAN model
does.

```
a_pred <- rstan::extract(fit_DET_binom_Pentagon_Interact, "a_pred")[[1]] #a (short for alpha) was used to represent to probability of success in our binomial model. It is equivalent to DET
#Assign conditions to predictions by iterating through species and sources, as in the STAN model
i <- 0
interaction_factors <- vector(length = ncol(a_pred))
for(j in levels(DETs_Pentagon$Species)){
  for(k in levels(DETs_Pentagon$Source)){
    i <- i+1
    interaction_factors[i] <- paste(j,k, sep = "X") #sep value used for parsing into factors later
  }
}
colnames(a_pred) <- interaction_factors
```

Finally, we can plot the posterior distributions of the alpha
estimates, allowing us to make inferences as described in the results
section of the associated manuscript “Mild movement sequence repetition
in five primate species and evidence for a taxonomic divide in cognitive
mechanisms”

```
pal <- c("#E69F00", "#56B4E9", "#009E73", "#F0E442")
plot_colors <- rep(pal, each = 4)
names(plot_colors) <- colnames(a_pred)

base_plot <- a_pred %>% 
  data.frame() %>%
  pivot_longer(cols = everything(), names_to = c("Species", "Source"), names_sep = "X", values_to = "alpha") %>%
  mutate(Source = factor(Source, levels = c("Learning_Factor1", "Learning_Factor1.2", "Learning_Factor2", "Experimental"))) %>% 
  ggplot()

base_plot <- base_plot + 
  geom_density_ridges(aes(x = alpha, y = interaction(Source, Species), fill = Species), rel_min_height = 0.01) +
  theme_classic() +
  theme(panel.grid.major.y = element_line(color = "grey", linetype = "dotted")) +
  geom_vline(aes(xintercept = 0),color = "grey", linetype = "dotted")  +
  scale_y_discrete(labels = c("Learning Factor 1", "Learning Factor 1.2", "Learning Factor 2", "Empirical", "Learning Factor 1", "Learning Factor 1.2", "Learning Factor 2", "Empirical", "Learning Factor 1", "Learning Factor 1.2", "Learning Factor 2", "Empirical", "Learning Factor 1", "Learning Factor 1.2", "Learning Factor 2", "Empirical")) +
  scale_fill_manual(values = pal, labels = c("Vervet")) +
  labs(y = "Source", x = "logit(DET)")

ggsave(file = "../Results/Credible Intervals of alpha_Pentagon.tiff",
       plot = base_plot,
       units = "px",
       dpi = 300)
```

```
## Saving 2100 x 1500 px image
```

```
## Picking joint bandwidth of 0.0131
```

```
base_plot
```

```
## Picking joint bandwidth of 0.0131
```
